# Occupational endotoxin exposure, DNA methylation changes, and immune-cell remodeling: an epigenome-wide association study in two cohorts

**DOI:** 10.64898/2026.09.29.26364351

**Published:** 2026-10-01

**Authors:** Hantao Wang, Mohammad L. Rahman, Charles E. Breeze, Gabriel Goodney, Jungeun Lim, Wei Hu, Batel Blechter, Jianxin Shi, Fengying Zhang, Qiuyin Cai, Gong Yang, Jirong Long, Li Su, Yu-Tang Gao, Xiao-Ou Shu, Nathaniel Rothman, Wei Zheng, Qing Lan, David C. Christiani, Jason Y.Y. Wong

**Affiliations:** 1 Population Sciences and Epidemiology Branch, National Heart, Lung, and Blood Institute, National Institutes of Health, 10 Center Drive, Bethesda, MD, USA.; 2 Division of Cancer Epidemiology and Genetics, National Cancer Institute, National Institutes of Health, 9609 Medical Center Drive, Rockville, MD, USA.; 3 Department of Clinical Sciences, Colorado State University, 1678 Campus Delivery, Fort Collins, CO, USA; 4 Shanghai Putuo District People s Hospital, No. 1291, Jiangning Rd, Putuo District, Shanghai, China; 5 Division of Epidemiology, Department of Medicine, Vanderbilt University Medical Center and Vanderbilt-Ingram Cancer Center, Nashville, Tennessee, USA.; 6 Department of Environmental Health, Harvard T.H. Chan School of Public Health, 665 Huntington Avenue, Boston, MA, USA.; 7 Shanghai Cancer Institute, Shanghai, China.; 8 Harvard Medical School, 25 Shattuck St, Boston, MA, USA.; 9 Massachusetts General Hospital, Division of Pulmonary and Critical Care Medicine, 55 Fruit Street, Boston, MA, USA.

## Abstract

**Background:**

Millions of textile and agricultural workers inhale organic dust containing endotoxin, an exposure associated with impaired respiratory function. However, the underlying biological pathways remain unclear. We conducted a two-cohort epigenome-wide association study (EWAS) to identify airborne endotoxin-associated DNA methylation (DNAm) alterations and pathways.

**Methods:**

In the discovery phase, we compared blood DNAm in 281 endotoxin-exposed cotton workers and 253 unexposed silk-workers from the Shanghai Textile Worker Study (STWS) measured using Illumina MethylationEPICv2 arrays. STWS endotoxin assessment combined detailed work histories and onsite measurements. For targeted parallel validation, we tested STWS signals in 686 incident lung cancer cases and 683 matched controls from the Shanghai Women’s Health Study (SWHS), using occupation-derived low, medium, or high exposure-intensity ranks based on published evidence. To gain biological insight, we analyzed pathway enrichment, immune-cell composition, DNAm inflammation risk scores (IRSs), and respiratory phenotype associations in STWS.

**Results:**

In STWS, we identified 36 epigenome-wide significant DNAm sites (P<5×10^−8^), of which, 35 were hypomethylated. Among 30 sites testable in SWHS, 18 had concordant effect directions, and four were nominally significant: cg11869499 (*POLG*; P=0.041), cg16290931 (*TSKS*; P=0.044), cg05019530 (*EPIC1*; P=0.042), and cg26033526 (*PSMB9/TAP1*; P=0.013). Enrichment implicated immune and signal-transduction pathways, including IgSF cell-adhesion-molecule signaling (FDR=8.2×10^−6^). Neutrophils were higher in cotton workers than in silk workers (63.5% vs 58.9%, p=7.52×10^−10^). Cumulative endotoxin was associated with higher CRP-based IRSs, which showed the most consistent associations with lower lung function and airflow obstruction.

**Conclusions:**

Our EWAS findings suggest that occupational endotoxin may operate partly through immune-cell composition and inflammatory pathways.

## Introduction

Endotoxin, or lipopolysaccharide (LPS), is a biologically active component of the outer membrane of Gram-negative bacteria released during bacterial growth and lysis. Endotoxin is common in occupational environments, with particularly high concentrations in the textile and agricultural industries. Workers who handle raw plant materials, livestock, or organic dusts are exposed mainly through inhalation. Among cotton textile workers and other exposed groups, endotoxin has been associated with acute and chronic respiratory effects (1–9).

Endotoxin affects the respiratory system largely through immune activation and inflammation. The CD14/MD-2/Toll-like receptor 4 (TLR4) complex recognizes LPS and activates MyD88- and TRIF-dependent signaling, leading to the production of proinflammatory cytokines (10–12). Previous analyses in cotton textile workers suggested that adaptive immunologic proteins may contribute to endotoxin-related lung function decline (9). However, the molecular pathways linking cumulative exposure to immune remodeling and subsequent respiratory impairment remain unclear.

Blood DNA methylation (DNAm) patterns can reflect environmental exposures (13, 14) and cellular states relevant to chronic lung disease (15). Many cytosine-phosphate-guanine (CpG) sites have immune-cell-specific methylation profiles, allowing immune-cell composition and exposure-related immune remodeling to be characterized from whole-blood DNAm (16). Furthermore, DNAm-derived inflammation risk scores (IRSs) can capture broader systemic inflammatory processes (17–20). Although experimental studies have shown that LPS can alter epigenetic regulation (21), little is known about the blood methylome response to long-term endotoxin exposure in humans. Examining this response may help identify specific biological pathways linking cumulative exposure with respiratory impairment.

To investigate molecular responses to occupational endotoxin exposure, we analyzed blood methylome data from two independent prospective cohorts. In the discovery phase, we conducted an untargeted epigenome-wide association study (EWAS) in the Shanghai Textile Worker Study (STWS) to identify differentially methylated positions (DMPs) associated with occupational exposure to endotoxin. Subsequently, we conducted targeted parallel validation of the identified signals (referred to as “validation” for brevity) in the population-based Shanghai Women's Health Study (SWHS) (22–25) using occupation-derived ordinal exposure-intensity rank. We also examined pathway enrichment, immune-cell composition, and DNAm-derived IRSs to characterize endotoxin-related immune remodeling. Finally, we explored associations between endotoxin-related DNAm biomarkers and respiratory phenotypes in the STWS.

## Methods

### Study populations

#### Discovery EWAS Phase:

The study design, questionnaires, endotoxin exposure assessment and outcome assessment in STWS have been described previously (1–9). Briefly, the STWS is a longitudinal occupational cohort that prospectively followed 447 endotoxin-exposed cotton workers and 472 unexposed control silk workers in Shanghai, China from 1981 to 2016, with follow-up surveys approximately every 5 years. Blood samples collected in 1996 were available for 284 cotton and 255 silk workers. The Institutional Review Boards (IRBs) of the Harvard T.H. Chan School of Public Health and Putuo District Hospital approved the STWS.

#### Targeted Parallel Validation:

The SWHS is a prospective cohort that enrolled 74,941 women aged 40–70 years from Shanghai, China between 1996 and 2000 (22, 23). Participants completed in-person interviews at enrollment and follow-up that collected demographic, lifestyle, and occupational information (24). We used a nested case-control sample of incident lung cancer cases and individually matched controls (25). Controls were alive and at risk on each case’s index date and were matched by date of birth (±2 years) and date of blood collection (±3 months). All participants in this subset were never-smokers and had provided pre-diagnostic blood samples at enrollment. The SWHS was approved by IRBs at the Vanderbilt University Medical Center and Shanghai Cancer Institute.

### Endotoxin Exposure Assessment

#### Discovery EWAS Phase:

The primary STWS exposure contrasted cotton workers with silk-worker controls. Full-shift area samples of cotton dust were collected onsite at every survey prior to 2001 using vertical elutriators. Endotoxin concentrations were measured using the Limulus Amebocyte Lysate assay. Previous validation showed that area measurements correlated closely with personal measurements. Samples from silk mills contained nearly undetectable endotoxin concentrations (<0.001 EU/m^3^) (26, 27).

Cumulative endotoxin concentration (EU/m^3^-year) and cotton dust concentration (mg/m^3^-year) were estimated using onsite samplings, job titles, and work history. Average 8-hour endotoxin and dust concentrations were calculated from cumulative exposure and years worked. Years since cessation of endotoxin exposure were derived from work history and were set to zero for silk workers. To examine the exposure-response pattern, we defined a four-level ordinal exposure variable with silk workers as the reference group and cotton workers classified by average 8-hour endotoxin concentration as <90, 90 to <1,700, or ≥1,700 EU/m^3^.

#### Targeted Parallel Validation:

Each participant’s occupation was collected in questionnaires and coded according to the Chinese National Standard Occupation and Industry Codes Manual. We used each occupation code to assign an occupation-derived exposure-intensity rank. This rank represented relative occupational exposure potential rather than individually measured endotoxin concentrations. Occupations were first grouped into broad occupational categories and assigned low, medium, or high ordinal exposure-intensity rank based on published exposure literature (Detailed in Supplemental Table 1 and 2). An occupation's rank was raised above its broad-group assignment when its usual tasks were expected to involve more frequent or direct contact with the principal carriers of endotoxin, and lowered when such contact was expected to be less (28).

### Epigenome-wide DNA methylation profiling

#### Discovery EWAS Phase:

DNAm was profiled in bisulfite-converted genomic DNA extracted from banked peripheral blood using the Illumina Infinium MethylationEPIC v2.0 BeadChip (EPIC v2; 930K) (29). DNAm data pre-processing and quality control (QC) were performed using SeSAMe (30), including restriction to standard CpG probes, Infinium I channel inference, nonlinear dye-bias correction, pOOBAH-based detection assessment, noob background correction, and Infinium I/II design-bias correction. We then excluded CpG probes with ≥50% missing values and samples with >10% missing values, applied the array-specific EPIC v2 general mask, and removed sex-chromosome probes and beta-distribution outliers. We imputed remaining missing values using k-nearest neighbors (k=10). We then used ComBat (31) to adjust for batch defined by the interaction of Sample Well and Sentrix ID while preserving occupation group, sex, and smoking status. After mapping EPIC v2 probes to EPIC-compatible probes, we estimated immune-cell fractions with EpiDISH (16). Before the EWAS, we removed samples with duplicated participant ID.

#### Targeted Parallel Validation:

The National Cancer Institute Cancer Genomics Research Laboratory profiled DNAm in peripheral-blood DNA using the Infinium MethylationEPIC BeadChip (EPIC v1; 850K) (32). QC has been detailed previously (25). ComBat was used for batch-effect correction (31), with Illumina slide as the batch variable. We tested only signals identified in the STWS discovery phase. Probe identifiers were harmonized between EPIC v1 and v2 arrays using the mLiftover function in SeSAMe (33).

### Respiratory outcome assessment

In the STWS, forced expiratory volume in 1 second (FEV-1) and forced vital capacity (FVC) were measured approximately every 5 years following American Thoracic Society spirometry standards (34). Percent-predicted FEV-1 and FVC and the lower limit of normal (LLN) for FEV-1/FVC were calculated using Global Lung Function Initiative (GLI) race-neutral reference equations (35, 36). Annual change in FEV-1 and FVC were calculated between consecutive surveys. Respiratory symptoms were collected using self-reported physician-diagnosis questionnaires at each survey.

### Statistical Analysis

Figure 1 summarizes the study design and analysis workflow.

### Discovery Phase in STWS

In the discovery EWAS, we used linear mixed models to regress DNAm beta values on occupational group, comparing endotoxin-exposed cotton workers with silk-worker controls. Models adjusted for age, sex, body mass index (BMI), smoking pack-years, years since exposure cessation, and EpiDISH-estimated immune-cell fractions. Nested random intercepts for sample plate and Sentrix ID accounted for residual technical effects. We used robust sandwich variance estimators to address correlated observations and heteroskedasticity.

Epigenome-wide significance was defined as p<5×10^−8^. DMPs were excluded if P>0.05 or inconsistent effect directions in sensitivity analyses using simple linear regression. To assess possible nonlinearity, we repeated the analysis using a four-level ordinal endotoxin-exposure variable. Additional sensitivity analyses used cumulative endotoxin and cumulative dust as alternative exposure metrics. We classified DMPs as potentially reversible when years since exposure cessation was associated with methylation at P<0.05 in the direction opposite to the endotoxin effect.

We also examined published DNAm-derived IRSs(17–20), and epigenetic age acceleration (EAA) estimated with dnaMethyAge (37). The construction of IRS_(Ligthart)_, IRS_(Wielscher)_, IRS_(Hillary)_, and IRS_(Stevenson)_ has been previously reported (25). We regressed each standardized IRS and EAA on log-transformed cumulative endotoxin in separate models adjusted for age, sex, BMI, smoking pack-years, years since exposure cessation, and EpiDISH-estimated immune-cell fractions.

### Targeted Parallel Validation in SWHS

DMPs identified in STWS were tested in SWHS using occupation-derived ordinal exposure-intensity ranks. The main validation model compared high vs. low and medium ordinal exposure-intensity rank, with age, BMI, education, case-control status, immune cell fractions, sample well, and a case-control pair random intercept. Sensitivity analyses included inverse probability weighting (IPW) for case-control status and alternative exposure metrics. Associations of ordinal exposure-intensity rank with EAA and IRS were evaluated using linear mixed models adjusted for the same covariates.

### Pathway enrichment and immune-cell remodeling

Pathway enrichment was evaluated using gene set enrichment analysis (GSEA) of ranked gene-level significance (38) against Gene Ontology (GO) terms, Kyoto Encyclopedia of Genes and Genomes (KEGG) pathways, and Reactome pathways. We considered gene sets with a false discovery rate (FDR) <0.05 significant and collapsed related sets into representative pathways. We then summarized leading-edge genes that contributed to multiple enriched pathways. In parallel, we used over-representation analysis (ORA) to identify pathways represented among genes mapped to the endotoxin-associated DMPs (39).

To characterize immune-cell remodeling associated with occupational endotoxin exposure, we examined immune-cell composition, cell-type-specific methylation, and regulatory enrichment. Immune-cell fractions between cotton and silk workers were compared using t-tests. We used CellDMC to identify cell-type-specific differential methylation associated with occupations (40) and eFORGE v2.0 (41) to test whether the endotoxin-associated DMPs overlapped tissue- or cell-type-specific regulatory annotations.

### Targeted DNAm biomarker and respiratory phenotype analyses

To evaluate whether endotoxin-related DNAm biomarkers were associated with respiratory phenotypes, we evaluated associations of endotoxin-related DMPs and IRSs with selected concurrent and prospective lung-function and airflow phenotypes. Models adjusted for log cumulative endotoxin, baseline age, sex, height, years since exposure cessation, smoking pack-years, BMI, immune-cell fractions, and sample plate. Age, sex, and height were omitted for percent predicted FEV-1 and FVC. DMP associations were expressed per 1-SD higher methylation beta value. To assess longitudinal lung function trajectories, we used linear mixed models to estimate associations of 1996 IRSs with FEV-1 and FVC from 1996 through 2016, measured at approximately 5-year intervals, with IPW accounting for loss to follow-up and random intercepts for individual variability. Additional IRS-associated lung function change over follow-up was assessed separately using interactions of IRS and time (*P*trend).

## Results

### Participant characteristics

#### Discovery EWAS Phase:

After sample-level QC and removal of duplicate records, the final STWS analytic sample included 281 cotton workers and 253 silk workers (Supplemental Figure 1). Cotton and silk workers were quantitatively similar in age, sex distribution, smoking patterns, and BMI (Table 1). Cotton workers had more respiratory symptoms, slightly lower percent-predicted FEV-1, and greater annual FEV-1 decline.

#### Targeted Parallel Validation:

The SWHS subset included 1,369 participants, with 686 incident lung cancer cases and 683 matched controls (Supplemental Table 3). Overall, 214 participants (15.6%) worked in textile occupations. The ordinal exposure-intensity rank was high for 213 participants (15.6%), medium for 113 (8.3%), and low for 1,043 (76.2%). Exposure-intensity rank did not differ by case-control status (chi-square P=0.54)

### DNAm biomarkers associated with occupational endotoxin exposure

#### Discovery in STWS:

We identified 36 DMPs associated with occupational endotoxin exposure when comparing cotton and silk workers (Table 2). The three strongest signals were cg22740441 (*PPP3CA*; P=4.76 × 10^−11^), cg11248361 (*AQP4-AS1*; P=1.75 × 10^−10^), and cg08009902 (*MTA3*; P=6.05 × 10^−10^). Thirty-five of the 36 DMPs were hypomethylated. Supplemental Figure 2 shows their genomic locations.

For 15 DMPs, including cg22740441, years since exposure cessation was associated with methylation at P<0.05 in the direction opposite to the endotoxin effect. These results suggest partial reversal of endotoxin-associated hypomethylation after exposure cessation. Supplemental Figure 3 shows adjusted DNAm beta values by cumulative endotoxin exposure and years since exposure cessation.

Compared with silk workers, the low-exposure cotton group showed nominal associations (P<0.05) for 35 of 36 DMPs; the medium- and high-exposure groups showed nominal associations for all 36. Ordered trend tests reached P<0.05 for 34 DMPs. Across all three cotton-exposure groups, the effect direction matched the primary EWAS for each DMP. Much of the exposure-category contrast separated silk workers from workers with any cotton exposure. For 21 DMPs, the low-exposure group had the largest absolute difference from silk workers (Supplemental Table 4).

Cumulative endotoxin was positively associated with all CRP-based IRSs. For each 2.72-fold increase in cumulative endotoxin, associations across the four CRP-based scores ranged from 0.011 SD for IRS_(Ligthart)_ (95% CI, 0.002 to 0.021) to 0.021 SD for IRS_(Hillary-elastic-net)_ (95% CI, 0.011 to 0.031). In contrast, the IL-6-based IRS_(Stevenson)_ was not significantly associated with cumulative endotoxin (Figure 2D). EAA estimates were generally positive, ranging from approximately 1 to 49 additional days per 2.72-fold increase in cumulative endotoxin (Supplemental Figure 4C).

#### Targeted Parallel Validation in SWHS:

Of the 36 STWS DMPs, 30 were available on the EPIC v1 array used in SWHS. In the primary model comparing high with low or medium exposure-intensity rank, 18 CpGs had concordant effect directions in STWS and SWHS. Four were nominally significant: cg11869499 (*POLG*; P=0.041), cg16290931 (*TSKS*; P=0.044), cg05019530 (*EPIC1*; P=0.042), and cg26033526 (*PSMB9/TAP1*; P=0.013) (Table 2, column B). Supplemental Table 5 reports the sensitivity analyses.

EAA measures showed no clear associations with exposure-intensity rank in SWHS. The CRP-based IRS_(Ligthart)_, IRS_(Wielscher)_, and IRS_(Hillary)_ showed positive associations with high exposure-intensity rank, consistent with the STWS results (Supplemental Figure 4).

### Pathway Enrichment and Immune-Cell Remodeling

#### Pathway enrichment

GSEA identified 152 significant representative gene sets, including 128 GO terms, 10 KEGG pathways, and 14 Reactome pathways (FDR<0.05; Supplemental Figure 5). KEGG and Reactome results centered on immune-system and signal-transduction pathways. The top KEGG pathway was IgSF CAM signaling (hsa04517; 285 genes; FDR=8.20 × 10^−6^). The top Reactome pathway was Diseases of signal transduction by growth factor receptors and second messengers (R-HSA-5663202; 450 genes; FDR=3.66 × 10^−7^).

Leading-edge analysis identified *ITGB1* and *PIK3CA* as the most recurrent contributors, appearing in 34 and 32 collapsed representative pathways, respectively (Supplemental Table 6). Among genes annotated to endotoxin-associated DMPs, *PPP3CA*, the gene annotated to the most significant DMP (cg22740441), appeared in the most leading-edge pathways. It contributed to six GO biological-process pathways, with the strongest enrichment for positive regulation of cell adhesion (GO:0045785; FDR=0.00248). ORA also highlighted immune and antigen-processing pathways among DMP-mapped genes.

#### Immune-cell remodeling related to endotoxin exposure:

EpiDISH, CellDMC, and eFORGE analyses provided complementary evidence of immune-cell remodeling associated with occupational endotoxin exposure. EpiDISH-estimated neutrophil proportions were significantly higher in cotton workers compared with silk workers (63.5% vs. 58.9%; t-test P = 7.52×10^−10^; Figure 2A). Cotton workers also had lower estimated proportions of several lymphoid cell types, including NK cells (5.95% vs. 6.92%; P=1.47 × 10^−5^).

CellDMC identified 204 cell-type-specific DNAm signals at FDR<0.05. These signals were observed in neutrophils (n = 203) and regulatory T cells (Treg; n = 1) (Figure 2B). Seven of the 36 DMPs had nominal neutrophil-specific associations at P<0.05.

eFORGE (Figure 2C) found enrichment of the 36 DMPs in blood regulatory regions, including DNase-hypersensitivity sites in primary NK cells from peripheral blood (FDR=0.028, 15 overlapping DMPs). Primary T cells from peripheral blood showed suggestive enrichment (FDR=0.07, 14 DMPs). Collectively, these analyses indicate that cotton workers differed from silk workers in estimated immune-cell composition and cell-type-specific DNAm patterns.

### Targeted respiratory phenotype associations of endotoxin-related DNAm biomarkers

We evaluated 36 endotoxin-associated DMPs and five IRSs in relation to 11 concurrent respiratory phenotypes assessed in 1996 and 40 prospective respiratory phenotypes (Figure 3A and Supplemental Table 7). These targeted associations should be interpreted as exploratory.

Nominal DMP associations occurred most often for airflow and lung-function phenotypes. cg08009902 (*MTA3*) was associated with at least one FEV-1/FVC-based phenotype at every survey after 1996. The top STWS signal, cg22740441 (*PPP3CA*), was associated with percent-predicted FEV-1 in 2016 (3.03% per SD; 95% CI, 0.04 to 6.03). Among the four DMPs nominally validated in SWHS, cg16290931 (*TSKS*) was associated with annual FEV-1 change from 1996 to 2001 (4.46 mL/year per SD; 95% CI, 0.74 to 8.19), and cg05019530 (*EPIC1*) was associated with annual FVC change from 1996 to 2011 (−3.46 mL/year per SD; 95% CI, −6.17 to −0.76).

The CRP-based IRSs showed a more consistent pattern, particularly IRS_(Hillary-BayesPR)_ and IRS_(Hillary-elastic-net)_. Higher scores were associated with lower FEV-1 and FVC at several surveys. The other CRP-based scores had fewer associations. Associations with the IL-6-based IRS_(Stevenson)_ occurred mainly for airflow-obstruction phenotypes in 2011 and 2016.

In the longitudinal analysis, a 1-SD higher 1996 IRS_(Hillary-BayesPR)_ score was associated with 37.9 mL lower FEV-1 in 1996 (95% CI, −72.0 to −3.8). The corresponding estimate for IRS_(Hillary-elastic-net)_ was −33.2 mL (95% CI, −68.1 to 1.6). FEV-1 differences remained negative at later surveys but did not become more negative over follow-up (Ptrend=0.450 and 0.159, respectively). Baseline association between IRS and FVC were insignificant. However, the IRS-associated FVC difference became progressively more negative over time. The additional annual FVC difference was −1.83 mL/year for IRS_(Hillary-BayesPR)_ (95% CI, −3.24 to −0.41; Ptrend=0.011) and −2.15 mL/year for IRS_(Hillary-elastic-net)_ (95% CI, −3.58 to −0.73; Ptrend=0.003). (Figure 3B)

## Discussion

### Overall Summary:

In this EWAS of occupational endotoxin exposure, we identified 36 previously unreported endotoxin-associated DNAm sites in STWS, most of which were hypomethylated. Of the 30 signals testable in SWHS, 18 showed directionally concordant associations with occupational exposure-intensity rank, including four that were nominally significant. Analyses of immune-cell composition and cell-type-specific methylation linked cotton work with higher estimated neutrophil proportions and DNAm signals concentrated in neutrophils, regulatory T cells, and lymphoid regulatory regions. Pathway analyses likewise highlighted immune signaling, antigen processing, cell adhesion, and receptor-mediated signaling. Endotoxin was also associated with higher CRP-based DNAm IRSs. In exploratory respiratory analyses, several endotoxin-related DMPs and, more consistently, CRP-based IRSs were associated with lung function and airflow obstruction phenotypes. Higher baseline IRSs were associated with lower FEV-1, while their associations with FVC became progressively more negative over follow-up. Together, these findings suggest that cumulative endotoxin exposure is associated with coordinated DNAm changes reflecting immune remodeling, inflammation, and respiratory impairment.

### Biological relevance of endotoxin-associated DMPs:

The top STWS signal, cg22740441 (*PPP3CA*), although not significant in targeted parallel SWHS validation, may still represent a biologically informative endotoxin-associated locus. *PPP3CA* encodes the catalytic subunit of calcineurin, a key component of calcineurin/NFAT signaling in immune cells. Experimental studies of another occupational inhalational exposure, methylene diphenyl diisocyanate, implicate increased *PPP3CA*/calcineurin signaling in inflammatory activation (42), whereas calcineurin signaling is also required for normal fetal lung maturation and surfactant production in mice (43). In STWS, cg22740441 was a potentially reversible DMP, and was associated with current dyspnea and prospective percent predicted FEV-1.

In addition, cg26033526 (*PSMB9/TAP1*), one of the four directionally concordant, nominally significant SWHS loci, has prior associations with HIV infection, inflammatory bowel disease, and eosinophilia (44–46). These observations identify immune-related loci that warrant mechanistic study in the context of occupational endotoxin exposure.

### Endotoxin-related immune remodeling, inflammation risk, and respiratory phenotypes:

We observed higher EpiDISH-estimated neutrophil proportions and lower proportions of several lymphoid cell types in cotton workers. CellDMC signals were also concentrated in neutrophils, supporting the cell-composition findings. Elevated blood neutrophil proportions may reflect inflammatory activity linked to airway injury and respiratory impairment (47–49). eFORGE enrichment in NK- and T-cell regulatory regions provided evidence of lymphoid involvement, consistent with studies linking cytotoxic lymphocytes and regulatory T-cell imbalance to lung-function decline (50–52). Together, these findings suggest that long-term endotoxin exposure is associated with coordinated innate inflammatory activation and adaptive immune regulation.

The IRS results provided a complementary measure of inflammation-related DNAm. Cumulative endotoxin was associated with CRP-based DNAm IRSs, whereas the IL-6 score was not significantly associated with endotoxin. This distinction is biologically plausible because IL-6 is an upstream inflammatory cytokine involved in CRP production (53), whereas circulating CRP is a downstream acute-phase protein with distinct production and clearance kinetics (54). In targeted respiratory-phenotype analyses, the CRP-based scores were associated with lower FEV-1 and airflow phenotypes, which is consistent with other population-based cohorts (20).

The convergence of our methylome and prior proteomic findings (9) on immune-related biology supports a role for immune processes in the association between endotoxin exposure and respiratory impairment, with the two omics layers capturing complementary biological time windows. Blood DNAm can provide a relatively stable epigenetic record of past exposure, which is supported by previous EWAS of smoking-associated marks that remained differentially methylated up to decades after cessation and reverted only partially (55). Consistent with this pattern, our endotoxin-associated DMPs likewise tracked years since cessation, suggesting partial reversibility. In contrast, circulating proteins may reflect more proximal inflammatory activity. Within the “meet-in-the-middle” framework (56), analyses integrating exposure, DNAm, proteins, and repeated respiratory measurements could test how these molecular signals relate across time.

### Occupational prevention implications:

Despite decades of study, the respiratory literature has not converged on a consensus exposure limit or no-observed-adverse-effect level (NOAEL) for endotoxin. In the Netherlands, an earlier health-based recommendation of 50 EU/m^3^ was later revised by the Dutch Expert Committee on Occupational Safety to 90 EU/m^3^ as an 8-hour time-weighted occupational exposure limit (57). In contrast, experimental cotton-cardroom work suggested no-FEV1-reaction thresholds of approximately 800–1700 EU/m^3^, depending on participant group (58). Previous studies in textile and agricultural workers further suggest that endotoxin effects on respiratory outcomes may be non-linear (6, 59).

The category-specific EWAS results were consistent with a threshold-like pattern at the identified DMPs. Cotton workers in the lowest exposure category (<90 EU/m^3^) differed from silk workers, while estimates were broadly similar across the low-, medium-, and high-exposure cotton groups. Although they do not establish a biological threshold, these findings reinforce the importance of minimizing endotoxin exposure even at relatively low occupational levels.

### Strengths and Limitations:

Our study also had notable strengths. STWS provided detailed historical exposure assessment, decades of follow-up, and repeated respiratory measurements. The EPICv2 array provided broad CpG coverage. Testing STWS signals in an independent cohort reduced reliance on potentially inflated discovery estimates, although differences in population and exposure assessment limited direct replications.

Despite these strengths, several limitations should be considered when interpreting our findings. First, DNAm was measured at a single time point rather than longitudinally. As such, we could not directly track within-person DNAm changes over time. Second, although SWHS provided an independent cohort for targeted parallel validation, endotoxin exposure was assessed using an occupation-derived ordinal exposure-intensity rank. The SWHS rank captured relative occupational exposure potential but not differences in specific tasks, exposure duration, or exposure variability within occupations. Such misclassification may have reduced statistical power and limited direct comparability between the two cohorts. Therefore, the SWHS analysis represents targeted parallel validation using a related exposure proxy rather than an exact replication of the STWS exposure metric. Additionally, SWHS differed from STWS in the distribution of sex, smoking status, and study design. Despite cohort heterogeneity, four loci still showed directionally concordant nominal associations. Finally, healthy-worker survivor effects and differential participation over long follow-up may have influenced observed associations. However, having a healthier study population would likely attenuate findings towards the null and lead to conservative estimation of the true effects.

### Conclusions:

Long-term occupational endotoxin exposure was associated with blood DNAm differences, estimated immune-cell remodeling, and enrichment of immune and inflammatory pathways. Endotoxin-related DMPs and CRP-based IRSs also showed exploratory associations with respiratory phenotypes. Independent replication and mechanistic studies are needed to establish the function and clinical relevance of these signals.

## Supplementary Material

1

## Figures and Tables

**Figure 1.**
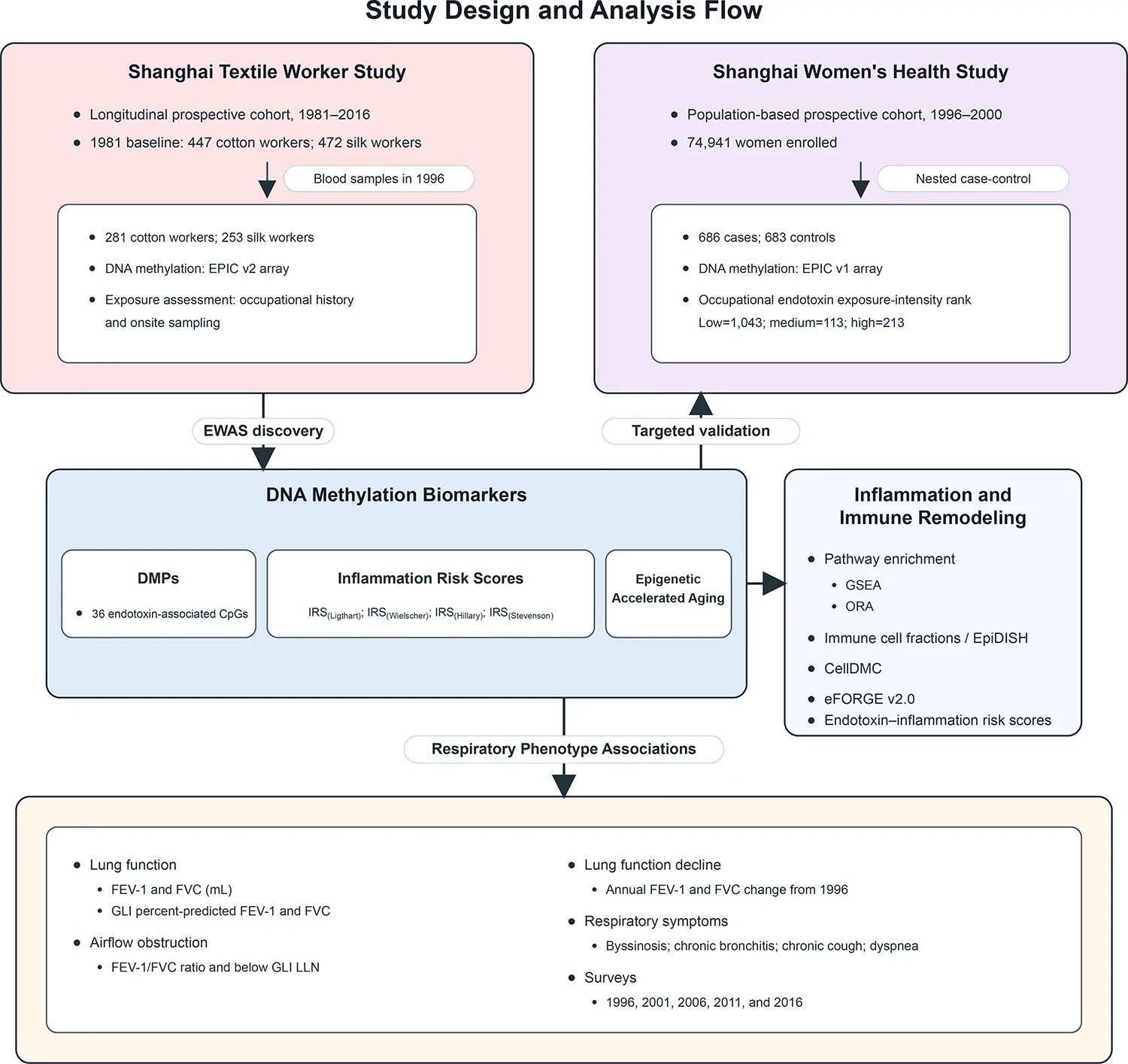
Study design and statistical analysis. This study aimed to identify DNA methylation changes associated with occupational exposure to endotoxin. STWS participants with blood samples collected in 1996 formed the EWAS discovery sample; a nested case-control sample from SWHS provided targeted validation. Endotoxin-associated DNA methylation biomarkers were evaluated for epigenetic accelerated aging, inflammation risk, immune remodeling, and respiratory phenotype associations. **Abbreviations:** CellDMC=cell-type-specific differential methylation; CpG=cytosine-phosphate-guanine; DMP=differentially methylated position; eFORGE v2.0=experimentally derived Functional element Overlap analysis of ReGions from EWAS, version 2.0; EPIC=Infinium MethylationEPIC array; EpiDISH=Epigenetic Dissection of Intra-Sample Heterogeneity; EWAS=epigenome-wide association study; FEV-1=forced expiratory volume in 1 second; FVC=forced vital capacity; GLI=Global Lung Function Initiative; GSEA=Gene Set Enrichment Analysis; IRS=inflammation risk score; LLN=lower limit of normal; ORA=Over-Representation Analysis; STWS=Shanghai Textile Worker Study; SWHS=Shanghai Women's Health Study.

**Figure 2.**
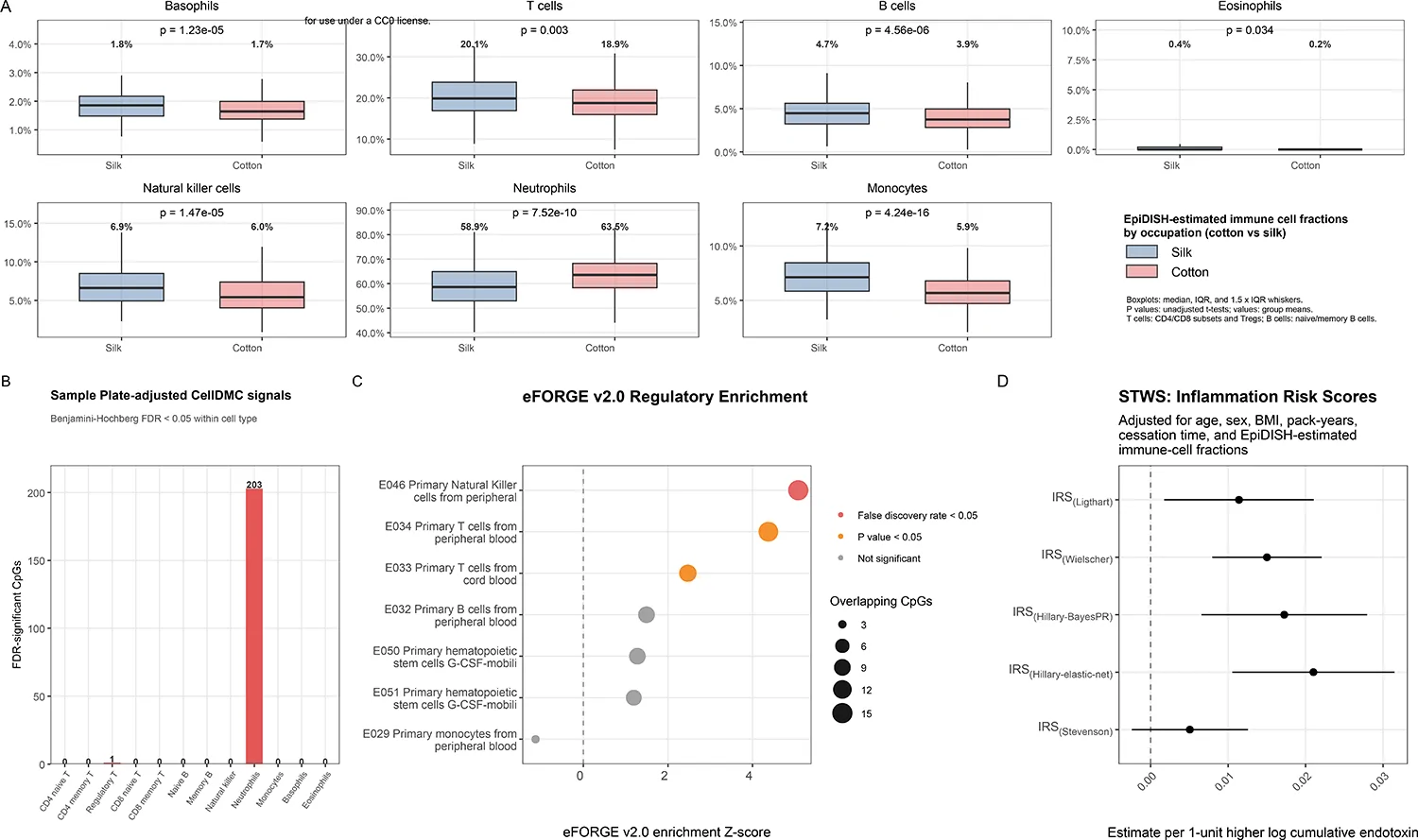
Immune remodeling and inflammation risk in relation to occupational endotoxin exposure. A) Boxplots of EpiDISH-estimated immune cell fractions by occupation (cotton versus silk); P values are from t-tests and numbers above boxes show group means. B) Number of CellDMC signals meeting Benjamini-Hochberg FDR<0.05 within each of 12 immune cell types. CellDMC modeled cotton-versus-silk occupation by EpiDISH-estimated immune-cell fractions and adjusted for age, sex, height, body mass index, smoking pack-years, years since exposure cessation, and Sample Plate. C) eFORGE v2.0 tests whether endotoxin-associated differentially methylated CpGs are enriched in tissue- or cell-type-specific regulatory annotations. D) Associations of cumulative endotoxin with inflammation risk scores in the Shanghai Textile Worker Study from linear models of each standardized score on log cumulative endotoxin, adjusted for age, sex, body mass index, smoking pack-years, years since exposure cessation, and EpiDISH-estimated immune-cell fractions. IRS_(Stevenson)_ is an interleukin-6-based score; the other inflammation risk scores are C-reactive protein-based scores. Abbreviations: BayesPR=Bayesian penalized regression; CellDMC=cell-type-specific differential methylation; CpG=cytosine-phosphate-guanine; EpiDISH=Epigenetic Dissection of Intra-Sample Heterogeneity; eFORGE v2.0=experimentally derived Functional element Overlap analysis of ReGions from Epigenome-Wide Association Studies, version 2.0; FDR=false discovery rate; IRS=inflammation risk score.

**Figure 3.**
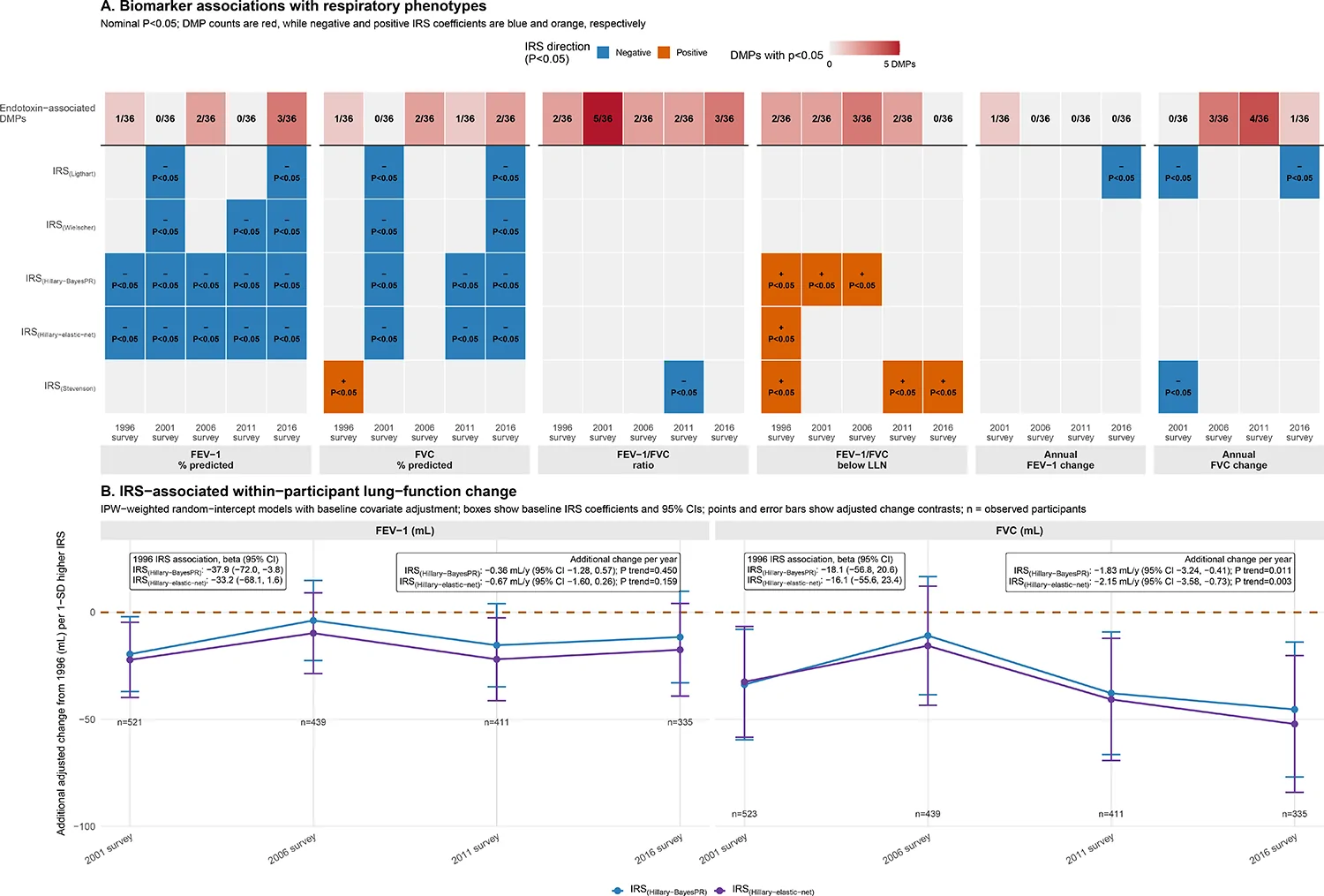
Respiratory phenotype associations with endotoxin–related DNA methylation biomarkers. (A) Heatmap of nominal associations for the 36 endotoxin–associated differentially methylated positions (DMPs) and individual inflammation risk scores (IRSs) with concurrent and prospective respiratory phenotypes; the DMP row gives the number of DMPs with P<0.05. In IRS rows, blue and orange indicate negative and positive model coefficients, respectively; only P<0.05 cells are colored. Colors encode coefficient sign rather than favorable or adverse interpretation, which depends on outcome coding. Separate multivariable linear or logistic regression models adjusted for log cumulative endotoxin, baseline age, sex, height, years since exposure cessation, pack–years, BMI, EpiDISH–estimated immune–cell fractions, and Sample Plate; GLI percent–predicted outcomes omitted age, sex, and height. (B) IPW–weighted random–intercept mixed models, adjusted for the same baseline covariates, estimated the association of 1–SD higher baseline IRS_(Hillary–BayesPR)_ and IRS_(Hillary–elastic–net)_ with FEV–1 and FVC at the 1996 survey and with additional change from 1996 at each subsequent survey; participation weights incorporated survey, prior attendance, baseline IRS, age, sex, pack–years, and cotton work. Boxes give the adjusted 1996 IRS beta coefficients and 95% confidence intervals; error bars are 95% confidence intervals, n denotes observed participants, and labels give the annual IRS–by–time slope, 95% confidence interval, and P trend. Abbreviations: BayesPR, Bayesian penalized regression; DMP, differentially methylated position; FEV–1, forced expiratory volume in 1 second; FVC, forced vital capacity; GLI, Global Lung Function Initiative; IPW, inverse–probability weighting; IRS, inflammation risk score; LLN, lower limit of normal.

**Table 1.** Participant Characteristics of Shanghai Textile Worker Study (STWS) in 1996

Variable	Cotton	Silk
**Sex**		
Female, %	165 (58.7%)	151 (59.7%)
Male, %	116 (41.3%)	102 (40.3%)
**Age, year**		
Mean (SD)	51.6 (10.2)	51.6 (10.1)
**Years worked, year**		
Mean (SD)	26.0 (8.0)	27.4 (9.2)
**Cumulative endotoxin, 1000 EU/m** ^3^ **-year**		
Mean (SD)	50.0 (45.0)	/
**Cumulative dust, mg/m** ^3^ **-year**		
Mean (SD)	18.6 (12.7)	/
**Smoking status**		
Never, %	183 (65.1%)	172 (68.0%)
Former, %	84 (29.9%)	67 (26.5%)
Current, %	14 (5.0%)	14 (5.5%)
**Smoking pack-years, pack-year**		
Mean (SD)	5.4 (10.5)	4.9 (10.2)
**Body mass index, kg/m** ^**2**^		
Mean (SD)	23.7 (3.2)	23.6 (2.9)
**Symptoms**		
Byssinosis, %	37 (13.2%)	0 (0.0%)
Chronic bronchitis, %	12 (4.3%)	4 (1.6%)
Chronic cough, %	9 (3.2%)	4 (1.6%)
Dyspnea, %	31 (11.0%)	18 (7.1%)
**FEV-1, ml**		
Mean (SD)	2438 (675)	2413 (593)
**% predicted FEV-1, %**		
Mean (SD)	86.2 (14.4)	87.3 (13.3)
**FVC, ml**		
Mean (SD)	3191 (780)	3157 (725)
**% predicted FVC, %**		
Mean (SD)	91.6 (12.1)	92.6 (10.8)
**Annual change in FEV-1 between 1992 and 1996, ml/year**		
Mean (SD)	−41.5 (39.6)	−34.4 (34.1)
**Annual change in FVC between 1992 and 1996, ml/year**		
Mean (SD)	−28.7 (57.6)	−15.1 (41.7)
**FEV-1/FVC ratio**		
Mean (SD)	0.762 (0.089)	0.765 (0.087)

Abbreviations: EU=endotoxin unit; FEV-1=forced expiratory volume in 1 second; FVC=forced vital capacity; SD=standard deviation; STWS=Shanghai Textile Worker Study. / indicates not available or not applicable.

**Table 2.** Summary of differentially methylated positions associated with occupational exposure to endotoxin in the Shanghai Textile Worker Study (STWS) and validation in the Shanghai Women's Health Study (SWHS).

CpG information				A: EWAS Discovery in STWS		B: Validation in SWHS	
CpG	Chromosome	Position	Gene Name(s)	Beta coefficient	P value	Beta coefficient	P value
**cg22740441**	**4**	**101328494**	**PPP3CA**	−**0.023**	**4.76e-11**	−**0.000**	**0.973**
**cg11248361**	**18**	**26742825**	**AQP4-AS1**	−**0.013**	**1.75e-10**	−**0.002**	**0.551**
cg08009902	2	42566376	MTA3	−0.013	6.05e-10	0.004	0.226
cg26277709	9	94726657	AOPEP; ENSG00000288023	−0.013	1.12e-09	0.001	0.106
cg26491213	2	44941679	SIX3; SIX3-AS1	−0.006	1.8e-09	0.001	0.245
**cg11869499** *	**15**	**89329752**	**POLG**	−**0.015**	**1.87e-09**	−**0.004**	**0.041**
**cg09660738**	**21**	**34560300**	**ENSG00000288711; RCAN1**	−**0.015**	**4.15e-09**	−**0.003**	**0.456**
**cg10793507**	**4**	**10656673**	**CLNK**	−**0.009**	**5.54e-09**	−**0.000**	**0.926**
**cg01778030**	**2**	**60053890**	**ENSG00000271955**	−**0.02**	**5.57e-09**	−**0.003**	**0.196**
cg04155673	10	63599202	REEP3	−0.004	5.63e-09	0.006	0.032
cg08129102	19	40014203	ZNF546	−0.006	6.53e-09	0.000	0.831
cg16039157	15	44715137	B2M	−0.022	8.38e-09	0.001	0.859
cg11800040	6	113065663	ENSG00000288560	−0.01	1.01e-08	/	/
cg19925755	10	103155793	NT5C2	−0.003	1.07e-08	0.001	0.356
cg22766862	17	60373383	USP32	−0.008	1.15e-08	/	/
cg05408620	3	120395583	FSTL1; LRRC58-DT	−0.019	1.32e-08	/	/
cg26947969	20	63201696	YTHDF1	−0.005	1.34e-08	0.001	0.719
cg01030629	5	142425830	SPRY4-AS1	−0.006	1.51e-08	/	/
cg19777190	14	49635703	DNAAF2	−0.022	1.52e-08	0.000	0.928
cg11795976	11	122116556	BLID; MIR100HG	−0.016	1.92e-08	0.000	0.909
**cg04522575**	**17**	**62965536**	**ENSG00000283538; TANC2**	−**0.024**	**1.95e-08**	−**0.002**	**0.527**
cg27039003	12	70050170	PRANCR	−0.025	2.1e-08	/	/
**cg10243623**	**11**	**128302353**	**ENSG00000289093**	−**0.025**	**2.23e-08**	−**0.007**	**0.083**
**cg26033526** *	**6**	**32852080**	**PSMB9; TAP1**	−**0.028**	**2.55e-08**	−**0.011**	**0.013**
**cg05801388**	**7**	**66087242**	**ASL; ENSG00000249319**	−**0.008**	**2.63e-08**	−**0.001**	**0.217**
**cg10264003**	**11**	**110328239**	**ENSG00000290078**	−**0.018**	**2.77e-08**	−**0.002**	**0.368**
**cg16290931** *	**19**	**49747296**	**TSKS**	−**0.007**	**2.79e-08**	−**0.002**	**0.044**
**cg15499393**	**2**	**7748812**	**ENSG00000290050**	−**0.017**	**3.05e-08**	−**0.003**	**0.457**
**cg00495557**	**16**	**86347319**	**ENSG00000269826; LINC00917**	−**0.023**	**3.6e-08**	−**0.005**	**0.226**
**cg19055984**	**6**	**15402639**	**JARID2**	**0.023**	**3.6e-08**	**0.001**	**0.716**
**cg01833052**	**9**	**78133908**	**/**	−**0.019**	**3.63e-08**	−**0.005**	**0.153**
cg12318285	4	53132749	SCFD2	−0.022	3.8e-08	/	/
**cg05019530** *	**22**	**47944045**	**EPIC1**	−**0.022**	**3.9e-08**	−**0.007**	**0.042**
**cg00916310**	**10**	**3810254**	**ENSG00000287175**	−**0.019**	**4.47e-08**	−**0.004**	**0.097**
cg25022932	20	33490001	CBFA2T2	−0.006	4.49e-08	0.000	0.622
cg16371865	8	140287706	TRAPPC9	−0.003	4.96e-08	0.000	0.770

A presents the STWS discovery analysis comparing cotton workers (n=281) with silk workers (n=253). ComBat-corrected DNAm beta values were analyzed using linear mixed models adjusted for age, sex, body mass index, smoking pack-years, years since cessation of endotoxin exposure, and EpiDISH-estimated immune-cell fractions, with nested random intercepts for Sample Plate and Sentrix ID. DMPs met epigenome-wide significance in the primary model (P<5 × 10^-8), had a directionally concordant robust-sandwich association at P<0.05, and had a directionally concordant association in the raw-beta sensitivity model. B presents the SWHS targeted-validation analysis comparing high with combined low/medium ordinal exposure-intensity rank. DNAm beta values were analyzed using linear mixed models adjusted for age, body mass index, education, case-control status, EpiDISH-estimated immune-cell fractions, and Sample Well, with a random intercept for matched case-control set. Rows in bold indicate directionally concordant STWS and SWHS beta coefficients (18 of 30 CpGs available in SWHS).

* denotes directionally concordant CpGs with P<0.05 in the main SWHS validation model (n=4). / indicates not available. Abbreviations: BMI=body mass index; CpG=cytosine-phosphate-guanine site; DMP=differentially methylated position; DNAm=DNA methylation; EpiDISH=Epigenetic Dissection of Intra-Sample Heterogeneity; STWS=Shanghai Textile Worker Study; SWHS=Shanghai Women's Health Study.

## Data Availability

STWS data used in this study are available upon reasonable request and subject to approval by the Institutional Review Board of the Harvard T.H. Chan School of Public Health and the Putuo Hospital Institutional Review Board. A data use agreement must be in place prior to data transfer. For inquiries, please contact Dr. David Christiani at: dchris@hsph.harvard.edu. Access to the SWHS datasets and biospecimens is granted on a case-by-case basis through the SWHS Research Committee via a formalized research application at: https://swhs-smhs.app.vumc.org/forResearchers.php. Programming code used in the study is available upon request.
