# Supplementary material for: Occupational endotoxin exposure, DNA methylation changes, and immune-cell remodeling: an epigenome-wide association study in two cohorts
Source: medRxiv. 2026 Oct 1:2026.09.29.26364351. Preprint. [Version 1] doi: 10.64898/2026.09.29.26364351 (PMC13648807; doi:10.64898/2026.09.29.26364351)
Supplement: Supplementary file 1 [file NIHPP2026.09.29.26364351V1-supplement-1.pdf]

medRxiv preprint doi: https://doi.org/10.64898/2026.09.29.26364351; this version posted October 1, 2026. The copyright holder for this preprint
(which was not certified by peer review) is the author/funder, who has granted medRxiv a license to display the preprint in perpetuity.
This article is a US Government work. It is not subject to copyright under 17 USC 105 and is also made available
for use under a CC0 license.

Supplemental Tables and Figures Index

- Supplemental Table 1. Shanghai Women's Health Study occupation-code endotoxin risk dictionary (standalone XLSX; not reproduced in this PDF)
- Supplemental Table 2. Occupational risk of endotoxin in broad occupation groups from the occupation list of Shanghai Women's Health Study
- Supplemental Table 3. Participant characteristics of the nested case-control cohort of Shanghai Women's Health Study (SWHS)
- Supplemental Table 4. Annotation and summary of endotoxin-related differentially methylated positions with sensitivity analyses
- Supplemental Table 5. STWS primary and SWHS validation results for 36 differentially methylated positions
- Supplemental Table 6. Gene Set Enrichment Analysis leading-edge genes from collapsed representative pathways
- Supplemental Table 7. Nominally significant biomarker-respiratory outcome associations
- Supplemental Figure 1. Data quality-control workflow
- Supplemental Figure 2. Miami plot
- Supplemental Figure 3. Reversible CpG contour plots
- Supplemental Figure 4. Endotoxin associations with inflammation risk scores and epigenetic accelerated aging
- Supplemental Figure 5. Pathway enrichment

medRxiv preprint doi: https://doi.org/10.64898/2026.09.29.26364351; this version posted October 1, 2026. The copyright holder for this preprint
(which was not certified by peer review) is the author/funder, who has granted medRxiv a license to display the preprint in perpetuity.
This article is a US Government work. It is not subject to copyright under 17 USC 105 and is also made available
for use under a CC0 license.

Supplemental Table 2. Occupational risk of endotoxin in broad occupation groups from the occupation list of Shanghai Women's Health Study

Broad occupation group	Code range	General risk assignment	Count: low risk occupations	Count: medium risk occupations	Count: high risk occupations	Concentration reference from literature	Reference
Agriculture / forestry / animal husbandry (broad header)	EN600-EN600	High	0	0	1	Broad agricultural prior; dairy/livestock, seed/grain, and many farm settings exceed 50 EU/m3, with several studies reporting levels above 90 EU/m3.	[1,2,8-12]
Cleaning / waste disposal	EN522-EN522	High	0	0	1	Wastewater/sewage studies reported personal GM 27 EU/m3, task-based GM 64 EU/m3, and a personal range of 0.6-2093 EU/m3; domestic-waste transfer/sorting and composting can reach approximately 300-1000 EU/m3.	[4-7]
Crop agriculture	EN611-EN619	High	0	0	5	Agricultural seed handling reported GM 1800 EU/m3 and levels up to 274,000 EU/m3; grain/feed industry workers had GM 628 EU/m3.	[2,11,12]
Livestock / animal husbandry	EN631-EN639	High	0	0	5	California dairy workers had arithmetic mean 453 EU/m3; Danish livestock farmers had overall GM 988 EU/m3 with range below LOD to 374,000 EU/m3.	[2,8-10]
Raw food / grain / animal-food processing	EN781-EN785	High	0	3	2	Norwegian grain/feed industry workers had GM 628 EU/m3; agricultural seed processing reported GM 1800 EU/m3; potato-processing literature also supports endotoxin-rich raw food processing.	[11-14]
Textile / fiber processing	EN751-EN759	High	0	0	7	Textile/cotton studies reported high levels: overall GM 831 EU/m3 in an integrated cotton factory, overall GM 2160 EU/m3 in Nepal textile work, and recycling/carding GMs of 5110-6381 EU/m3; garment work was lower but still about 157 EU/m3.	[15-19,28]
Waste material recovery	EN433-EN433	High	0	0	1	Indoor domestic-waste transfer/sorting and composting can reach approximately 300-1000 EU/m3; outdoor or better-controlled recycling settings may be lower, so task setting is important.	[6,7]
Farm machinery / field operations	EN661-EN669	Medium	0	0	2	Agricultural seed, grain/feed, and livestock settings can substantially exceed 50 EU/m3; enclosed cabs or lower organic-dust tasks may reduce exposure.	[2,11,12]
Fishery / aquatic products	EN641-EN649	Medium	0	5	0	Seafood-processing studies document bioaerosol/endotoxin exposure and work-related respiratory effects, but quantitative EU/m3 levels are heterogeneous and task-dependent; broad-group baseline kept medium.	[24,25]
Forestry	EN621-EN629	Medium	1	4	0	New Zealand sawmills reported endotoxin range 7-588 EU/m3 with half of measurements above 50 EU/m3; Croatian wood-processing airborne samples ranged 166.8-671.6 EU/m3. Outdoor forestry and modern sawmill tasks can be lower, so broad baseline kept medium.	[20-23]
Leather / fur / feather processing	EN761-EN769	Medium	0	3	2	Direct EU/m3 data remain sparse; raw animal materials plausibly carry biologic contamination, but quantitative evidence is weaker than for cotton, grain, or livestock.	[2,7]
Other agriculture / forestry / animal husbandry	EN699-EN699	Medium	0	0	1	Heterogeneous mixed group; livestock, seed/grain, and wood-processing subsettings can exceed 50 EU/m3, but exact exposure depends strongly on task and process.	[2,8-12,20-23]
Other food / beverage / tobacco processing	EN786-EN790	Medium	0	3	2	Quantitative endotoxin evidence is heterogeneous. Grain/feed and seed or plant-material processing examples exceed 50 EU/m3, while other food or beverage tasks may be lower; medium baseline reflects possible raw biological dust.	[11-14]
Tailoring / sewing	EN771-EN779	Medium	0	5	0	Garment-sector work had GM about 157 EU/m3 in a textile study; broader cotton/textile settings can be substantially higher.	[18,19,28]
Wood / paper processing	EN801-EN819	Medium	2	5	2	New Zealand sawmills reported range 7-588 EU/m3 and Croatian wood-processing airborne samples ranged 166.8-671.6 EU/m3; exposure is task-dependent and can be below or above 50 EU/m3.	[20-23]
Administrative / managerial	EN211-EN242	Low	11	0	0	Limited direct evidence for occupational endotoxin exposure; no routine organic-dust source identified. Office/general-indoor comparator GM 0.24 EU/m3, well below 50 EU/m3.	[2,3]
Clerical / security / postal / telecom	EN311-EN399	Low	16	0	0	Limited direct evidence for occupational endotoxin exposure; no routine organic-dust source identified, so broad-group prior was considered below 50 EU/m3.	[2,3,7]
Commerce / sales / purchasing	EN411-EN499	Low	6	1	0	Limited direct evidence for occupational endotoxin exposure; no routine organic-dust source identified, so broad-group prior was considered below 50 EU/m3.	[2,3,7]
Construction	EN921-EN929	Low	6	0	0	Limited direct evidence for occupational endotoxin exposure; no routine organic-dust source identified, so broad-group prior was considered below 50 EU/m3.	[2,3,7]
Equipment operation / media / tape production	EN871-EN874	Low	4	0	0	Limited direct evidence for occupational endotoxin exposure; no routine organic-dust source identified, so broad-group prior was considered below 50 EU/m3.	[2,3,7]
General service work	EN511-EN599	Low	18	1	0	Limited direct evidence for occupational endotoxin exposure; no routine organic-dust source identified, so broad-group prior was considered below 50 EU/m3.	[2,3,7]
Glass / pottery / enamel	EN891-EN893	Low	3	0	0	Limited direct evidence for occupational endotoxin exposure; no routine organic-dust source identified, so broad-group prior was considered below 50 EU/m3.	[2,3,7]
Hunting	EN650-EN650	Low	1	0	0	Limited direct quantitative evidence; no evidence found for sustained endotoxin-rich organic-dust exposure in this broad group.	[2,7]
Inspection / testing / laboratory analysis	EN961-EN967	Low	4	3	0	Limited direct quantitative evidence; broad-group prior considered low unless occupation-specific work involved raw biologic materials, animals, or contaminated wet processes.	[2,7]
Loading / transport / shipping	EN941-EN959	Low	11	1	0	Limited direct evidence for occupational endotoxin exposure; no routine organic-dust source identified, so broad-group prior was considered below 50 EU/m3.	[2,3,7]
Machinery / electrical / electronic installation & maintenance	EN841-EN869	Low	15	0	0	Limited direct evidence for routine high exposure in this broad group; metalworking-fluid aerosols and contaminated water-based systems are possible respiratory-hazard exceptions.	[2,26,27]
Metal fabrication / welding / ductwork	EN881-EN884	Low	4	0	0	Limited direct evidence for routine high exposure in this broad group; contaminated-fluid or metalworking-fluid tasks can create exceptions, but this is not a routine organic-dust sector.	[2,26,27]
Mining / metals / chemicals / rubber / plastics	EN711-EN749	Low	26	0	0	Limited direct evidence for routine high exposure in this broad group; non-biologic industrial processes have no intrinsic endotoxin source, with contaminated-fluid exceptions.	[2,26,27]
Miscellaneous / warehouse / underwater	EN991-EN998	Low	6	0	0	Limited direct evidence for occupational endotoxin exposure; no routine organic-dust source identified, so broad-group prior was considered below 50 EU/m3.	[2,3,7]
Painting / non-metal mineral / other production	EN901-EN919	Low	11	0	0	Limited direct evidence for occupational endotoxin exposure; no routine organic-dust source identified, so broad-group prior was considered below 50 EU/m3.	[2,3,7]

medRxiv preprint doi: https://doi.org/10.64898/2026.09.29.26364351; this version posted October 1, 2026. The copyright holder for this preprint (which was not certified by peer review) is the author/funder, who has granted medRxiv a license to display the preprint in perpetuity.
This article is a US Government work. It is not subject to copyright under 17 USC 105 and is also made available for use under a CC0 license.

Broad occupation group	Code range	General risk assignment	Count: low risk occupations	Count: medium risk occupations	Count: high risk occupations	Concentration reference from literature	Reference
Power generation / boiler operations	EN931-EN939	Low	4	0	0	Limited direct evidence for occupational endotoxin exposure; no routine organic-dust source identified, so broad-group prior was considered below 50 EU/m3.	[2,3,7]
Printing / plate making	EN821-EN829	Low	6	0	0	Limited direct evidence for occupational endotoxin exposure; no routine organic-dust source identified, so broad-group prior was considered below 50 EU/m3.	[2,3,7]
Professional / technical / medical / education / arts	EN011-EN150	Low	78	8	1	Limited direct evidence for occupational endotoxin exposure; no routine organic-dust source identified for the broad group. Office/general-indoor comparator GM 0.24 EU/m3, well below 50 EU/m3.	[2,3]
Stone / glass / ceramics	EN831-EN839	Low	3	0	0	Limited direct evidence for occupational endotoxin exposure; no routine organic-dust source identified, so broad-group prior was considered below 50 EU/m3.	[2,3,7]

Broad-group ordinal exposure-intensity rank assignment used 50 EU/m3 as a conservative screening threshold for evidence of at least medium occupational endotoxin exposure intensity.

Supplemental Table 2 references

1. Health Council of the Netherlands, Dutch Expert Committee on Occupational Safety. Endotoxins: health-based recommended occupational exposure limit. The Hague: Health Council of the Netherlands; 2010. Report No.: 2010/04OSH.

2. Liebers V, Bruning T, Raulf-Heimsoth M. Occupational endotoxin-exposure and possible health effects on humans. Am J Ind Med. 2006;49(6):474-491. doi:10.1002/ajim.20310.

3. Hines CJ, Milton DK, Larsson L, Petersen MR, Fisk WJ, Mendell MJ. Characterization and variability of endotoxin and 3-hydroxy fatty acids in an office building during a particle intervention study. Indoor Air. 2000;10(1):2-12. doi:10.1034/j.1600-0668.2000.010001002.x.

4. Smit LAM, Spaan S, Heederik D. Endotoxin exposure and symptoms in wastewater treatment workers. Am J Ind Med. 2005;48(1):30-39. doi:10.1002/ajim.20176.

5. Spaan S, Smit LAM, Eduard W, Larsson L, Arts HJJM, Wouters IM, Heederik DJJ. Endotoxin exposure in sewage treatment workers: investigation of exposure variability and comparison of analytical techniques. Ann Agric Environ Med. 2008;15(2):251-261. PMID:19118445.

6. Wouters IM, Spaan S, Douwes J, Doekes G, Heederik D. Overview of personal occupational exposure levels to inhalable dust, endotoxin, beta(1-3)-glucan and fungal extracellular polysaccharides in the waste management chain. Ann Occup Hyg. 2006;50(1):39-53. doi:10.1093/annhyg/mei047.

7. Duquenne P, Marchand G, Duchaine C. Measurement of endotoxins in bioaerosols at workplace: a critical review of literature and a standardization issue. Ann Occup Hyg. 2013;57(2):137-172. doi:10.1093/annhyg/mes051.

8. Garcia J, Bennett DH, Tancredi D, Schenker MB, Mitchell D, Reynolds SJ, Mitloehner FM. Occupational exposure to particulate matter and endotoxin for California dairy workers. Int J Hyg Environ Health. 2013;216(1):56-62. doi:10.1016/j.ijheh.2012.04.001.

9. Basinas I, Sigsgaard T, Heederik D, Takai H, Omland O, Andersen NT, Wouters IM, Bonlokke JH, Kromhout H, Schlunssen V. Exposure to inhalable dust and endotoxin among Danish livestock farmers: results from the SUS cohort study. J Environ Monit. 2012;14(2):604-614. doi:10.1039/C1EM10576K.

10. Basinas I, Sigsgaard T, Kromhout H, Heederik D, Wouters IM, Schlunssen V. A comprehensive review of levels and determinants of personal exposure to dust and endotoxin in livestock farming. J Expo Sci Environ Epidemiol. 2015;25(2):123-137. doi:10.1038/jes.2013.83.

11. Straumfors A, Heldal KK, Wouters IM, Eduard W. Exposure to grain dust and microbial components in the Norwegian grain and compound feed industry. Ann Occup Hyg. 2013;57(9):1105-1114. doi:10.1093/annhyg/met036.

12. Smit LAM, Wouters IM, Hobo MM, Eduard W, Doekes G, Heederik D. Agricultural seed dust as a potential cause of organic dust toxic syndrome. Occup Environ Med. 2006;63(1):59-67. doi:10.1136/oem.2005.021527.

13. Zock JP, Heederik D, Kromhout H. Exposure to dust, endotoxin and microorganisms in the potato processing industry. Ann Occup Hyg. 1995;39(6):841-854. doi:10.1093/annhyg/39.6.841.

14. Health and Safety Executive. Flour dust and enzymes: advice for managers and employees in bakeries. Bootle: Health and Safety Executive. INDG429.

15. Kennedy SM, Christiani DC, Eisen EA, Wegman DH, Greaves IA, Olenchock SA, Ye TT, Lu PL. Cotton dust and endotoxin exposure-response relationships in cotton textile workers. Am Rev Respir Dis. 1987;135(1):194-200. doi:10.1164/arrd.1987.135.1.194.

16. Astrakianakis G, Seixas NS, Camp JE, Christiani DC, Feng Z, Thomas DB, Checkoway H. Modeling, estimation and validation of cotton dust and endotoxin exposures in Chinese textile operations. Ann Occup Hyg. 2006;50(6):573-582. doi:10.1093/annhyg/mel018.

17. Mehta AJ, Wang XR, Eisen EA, Dai HL, Astrakianakis G, Seixas N, Camp J, Checkoway H, Christiani DC. Work area measurements as predictors of personal exposure to endotoxin and cotton dust in the cotton textile industry. Ann Occup Hyg. 2008;52(1):45-54. doi:10.1093/annhyg/mem061.

18. Paudyal P, Semple S, Niven R, Tavernier G, Ayres JG. Exposure to dust and endotoxin in textile processing workers. Ann Occup Hyg. 2011;55(4):403-409. doi:10.1093/annhyg/meq084.

19. Lenters V, Basinas I, Beane-Freeman L, Boffetta P, Checkoway H, Coggon D, Portengen L, et al. Endotoxin exposure and lung cancer risk: a systematic review and meta-analysis of the published literature on agriculture and cotton textile workers. Cancer Causes Control. 2010;21(4):523-555. doi:10.1007/s10552-009-9483-z.

20. Oppliger A, Rusca S, Charriere N, Duc TV, Droz PO. Assessment of bioaerosols and inhalable dust exposure in Swiss sawmills. Ann Occup Hyg. 2005;49(5):385-391. doi:10.1093/annhyg/meh105.

medRxiv preprint doi: https://doi.org/10.64898/2026.09.29.26364351; this version posted October 1, 2026. The copyright holder for this preprint
(which was not certified by peer review) is the author/funder, who has granted medRxiv a license to display the preprint in perpetuity.
This article is a US Government work. It is not subject to copyright under 17 USC 105 and is also made available
for use under a CC0 license.

21. Douwes J, McLean D, van der Maarl E, Heederik D, Pearce N. Worker exposures to airborne dust, endotoxin and beta(1,3)-glucan in two New Zealand sawmills. Am J Ind Med. 2000;38(4):426-430. doi:10.1002/1097-0274(200010)38:4<426::AID-AJIM8>3.0.CO;2-R.

22. Dennekamp M, Demers PA, Bartlett K, Davies HW, Teschke K. Endotoxin exposure among softwood lumber mill workers in the Canadian province of British Columbia. Ann Agric Environ Med. 1999;6(2):141-146. PMID:10607995.

23. Pipinic IS, Varnai VM, Lucic AT, Prester L, Macan J. Endotoxin exposure assessment in wood-processing industry: airborne versus settled dust levels. Arh Hig Rada Toksikol. 2010;61(2):161-166. doi:10.2478/10004-1254-61-2010-1996.

24. Jeebhay MF, Robins TG, Miller ME, Bateman E, Smuts M, Baatjies R, Lopata AL. Occupational allergy and asthma among salt water fish processing workers. Am J Ind Med. 2008;51(12):899-910. doi:10.1002/ajim.20635.

25. Bang BE, Aasmoe L, Aamodt BH, Aardal L, Andorsen GS, Bjornbakk AK, Egeness C, Espejord I, Kramvik E. Exposure and airway effects of seafood industry workers in northern Norway. J Occup Environ Med. 2005;47(5):482-492. doi:10.1097/01.jom.0000161732.96555.2b.

26. Lillienberg L, Burdorf A, Mathiasson L, Thorneby L. Exposure to metalworking fluid aerosols and determinants of exposure. Ann Occup Hyg. 2008;52(7):597-605. doi:10.1093/annhyg/men043.

27. National Institute for Occupational Safety and Health. Criteria for a recommended standard: occupational exposure to metalworking fluids. Cincinnati: NIOSH; 1998. DHHS (NIOSH) Publication No. 98-102. doi:10.26616/NIOSHPUB98102.

28. Tefera Y, Schlunssen V, Kumie A, Deressa W, Moen BE, Bratveit M. Personal inhalable dust and endotoxin exposure among workers in an integrated textile factory. Arch Environ Occup Health. 2020;75(7):415-421. doi:10.1080/19338244.2020.1743958.

medRxiv preprint doi: https://doi.org/10.64898/2026.09.29.26364351; this version posted October 1, 2026. The copyright holder for this preprint (which was not certified by peer review) is the author/funder, who has granted medRxiv a license to display the preprint in perpetuity.
This article is a US Government work. It is not subject to copyright under 17 USC 105 and is also made available for use under a CC0 license.

Supplemental Table 3. Participant characteristics of the nested case-control cohort of Shanghai Women's Health Study (SWHS)

Variable	Control (N=683)	Case (N=686)
Age, year		
Mean (SD)	56.4 (8.9)	56.2 (8.8)
Body mass index, kg/m^2		
Mean (SD)	24.6 (3.5)	24.3 (3.5)
Education		
Less than middle school, %	91 (13.3%)	99 (14.4%)
Middle school, %	149 (21.8%)	160 (23.3%)
High school, %	205 (30.0%)	206 (30.0%)
College or higher, %	238 (34.8%)	221 (32.2%)
Annual family income		
Low, %	232 (34.0%)	222 (32.4%)
Middle, %	262 (38.4%)	263 (38.3%)
High, %	189 (27.7%)	201 (29.3%)
Sample type		
Blood, %	246 (36.0%)	247 (36.0%)
White blood cells, %	437 (64.0%)	439 (64.0%)
Occupational endotoxin risk		
Low, %	528 (77.3%)	515 (75.1%)
Medium, %	56 (8.2%)	57 (8.3%)
High, %	99 (14.5%)	114 (16.6%)
Textile occupation		
Yes, %	105 (15.4%)	109 (15.9%)

Characteristics are stratified by case-control status. Continuous variables are shown as mean (SD), categorical rows show counts and percentages, and binary variables show the Yes category only. / indicates not available or not applicable. Education and income labels were inferred from published Shanghai Women's Health Study baseline descriptions; confirm income1 coding against the analysis codebook if available. Abbreviations: BMI=body mass index; SD=standard deviation; SWHS=Shanghai Women's Health Study.

medRxiv preprint doi: https://doi.org/10.64898/2026.09.29.26364351; this version posted October 1, 2026. The copyright holder for this preprint (which was not certified by peer review) is the author/funder, who has granted medRxiv a license to display the preprint in perpetuity.
This article is a US Government work. It is not subject to copyright under 17 USC 105 and is also made available for use under a CC0 license.

Supplemental Table 4. Annotation and summary of endotoxin-related differentially methylated positions with sensitivity analyses

CpG annotation							CellDMC sensitivity analysis	Primary STWS EWAS		Categorical exposure sensitivity analysis							Subgroup sensitivity analyses				Alternative cumulative-exposure models	
CpG	Chromosome	Position	Gene Name(s)	CpG context	CpG type	Reversible	Neutrophils	Cotton vs Silk		Cotton Low vs Silk		Cotton Mid vs Silk		Cotton High vs Silk		Ordered trend	Never smokers	Ever smokers	Men	Women	ln cumulative endotoxin	ln cumulative dust
							P<0.05	Beta	P	Beta	P	Beta	P	Beta	P							
cg22740441	4	101328494	PPP3CA	open sea	Gene Body	Yes	Yes	-0.02	4.76e-11	-0.03	1.16e-09	-0.02	1.28e-08	-0.02	9e-10	0.00317	-5.5	-6.05	-5.91	-5.35	-6.72	-7.19
cg11248361	18	26742825	AQP4-AS1	open sea	Gene Body	Yes	No	-0.01	1.75e-10	-0.01	2.67e-05	-0.01	1.66e-06	-0.01	6.95e-10	3.68e-06	-3.86	-5.05	-5.68	-3.42	-6.87	-6.98
cg08009902	2	42566376	MTA3	shelf	Non-island Promoter	No	No	-0.01	6.05e-10	-0.01	0.000456	-0.01	7e-09	-0.01	2.39e-08	8.43e-05	-5.02	-3.71	-4.38	-4.23	-6.69	-6.72
cg26277709	9	94726657	AOPEP;ENSG00000288023	island	CpG island Promoter	No	Yes	-0.01	1.12e-09	-0.01	1.85e-06	-0.01	1.45e-08	-0.01	5.09e-07	0.00294	-4.96	-4	-3.78	-5.03	-6.26	-6.41
cg26491213	2	44941679	SIX3;SIX3-AS1	shore	Non-island Promoter	No	No	-0.01	1.8e-09	-0.01	2.52e-05	-0.01	5.76e-07	-0.01	9.45e-07	0.0275	-4.48	-4.48	-4.98	-3.99	-5.97	-6.04
cg11869499	15	89329752	POLG	shelf	Non-island Promoter	Yes	No	-0.01	1.87e-09	-0.02	1.59e-06	-0.01	0.000579	-0.02	2.75e-07	2.3e-05	-5.2	-4.33	-4.43	-4.94	-6.1	-6.4
cg09660738	21	34560300	ENSG00000288711;RCAN1	open sea	Non-island Promoter	Yes	No	-0.02	4.15e-09	-0.02	6.14e-07	-0.01	2.97e-06	-0.01	1.52e-07	0.0276	-4.91	-4.08	-5.74	-3.54	-4.02	-6.24
cg10793507	4	10656673	CLNK	open sea	Non-island Promoter	No	Yes	-0.01	5.54e-09	-0.01	1.49e-05	-0.01	9.98e-08	-0.01	1.25e-06	0.0203	-4.69	-3.25	-3.77	-3.15	-4.96	-5.85
cg01778030	2	60053890	ENSG00000271955	open sea	Non-island Promoter	No	No	-0.02	5.57e-09	-0.02	8.06e-07	-0.02	8.44e-08	-0.02	1.3e-07	0.00415	-4.62	-4.68	-5.15	-4.21	-6.02	-6.25
cg04155673	10	63599202	REEP3	open sea	Gene Body	No	No	0	5.63e-09	0	6.7e-06	0	8.77e-08	0	6.38e-08	0.00492	-6.16	-3.53	-3.43	-6.36	-6.05	-6.28
cg08129102	19	40014203	ZNF546	open sea	Gene Body	No	No	-0.01	6.53e-09	0	0.00136	-0.01	4.16e-10	-0.01	5.89e-07	0.00128	-4.91	-3.19	-3.66	-4.93	-6.26	-6.27
cg16039157	15	44715137	B2M	shelf	Non-island Promoter	No	No	-0.02	8.38e-09	-0.02	0.00224	-0.02	1.42e-07	-0.02	1.6e-07	1.63e-07	-4.04	-4.46	-5.27	-3.72	-6.17	-6.11
cg11800040	6	113065663	ENSG00000288560	open sea	Gene Body	No	No	-0.01	1.01e-08	-0.01	9.01e-06	-0.01	1.85e-05	-0.01	2.99e-08	0.000821	-4.56	-4.47	-5.1	-3.65	-6.02	-6.07
cg19925755	10	103155793	NT5C2	open sea	Non-island Promoter	No	No	0	1.07e-08	0	0.000203	0	1.09e-05	0	4.62e-07	6.63e-05	-4.94	-3.17	-3.12	-3.95	-5.73	-5.75
cg22766862	17	60373383	USP32	open sea	Gene Body	No	No	-0.01	1.15e-08	-0.01	2.31e-08	-0.01	6.83e-05	-0.01	1.46e-07	0.00271	-4.9	-4.33	-4.49	-4.43	-5.39	-6.05
cg05408620	3	120395583	FSTL1;LRRC58-DT	open sea	Gene Body	Yes	No	-0.02	1.32e-08	-0.01	0.0646	-0.02	0.00162	-0.02	1.47e-06	7.35e-07	-2.46	-4.71	-4.87	-2.89	-5.83	-5.95
cg26947969	20	63201696	YTHDF1	open sea	Gene Body	No	No	0	1.34e-08	-0.01	1.52e-06	-0.01	5.87e-08	0	2.45e-06	0.118	-5.03	-4.12	-4.84	-2.46	-4.61	-6.06
cg01030629	5	142425830	SPRY4-AS1	open sea	Gene Body	No	No	-0.01	1.51e-08	-0.01	3.1e-07	-0.01	8.2e-07	-0.01	1.79e-06	0.0126	-5.62	-3.18	-2.56	-5.79	-5.63	-5.9
cg19777190	14	49635703	DNAAF2	shore	Non-island Promoter	No	No	-0.02	1.52e-08	-0.02	0.000617	-0.02	5.95e-06	-0.02	6.38e-07	8.97e-05	-4.89	-3.26	-4.73	-3.4	-5.72	-5.74
cg11795976	11	122116556	BLID;MIR100HG	open sea	Non-island Promoter	Yes	No	-0.02	1.92e-08	-0.02	0.000806	-0.01	6.56e-05	-0.02	9.81e-08	1.72e-07	-4.33	-3.08	-4.05	-3.7	-5.73	-5.76
cg04522575	17	62965536	ENSG00000283538;TANC2	shore	Non-island Promoter	No	No	-0.02	1.95e-08	-0.03	7.99e-08	-0.02	4.92e-06	-0.02	6.58e-07	0.101	-4.75	-5.24	-5.37	-3.99	-4.45	-5.96
cg27039003	12	70050170	PRANCR	open sea	Gene Body	No	No	-0.03	2.1e-08	-0.03	7.21e-06	-0.02	2.15e-05	-0.03	2.11e-07	0.000843	-4.73	-3.66	-4.39	-4.47	-5.7	-6.01
cg10243623	11	128302353	ENSG00000289093	open sea	Gene Body	Yes	No	-0.02	2.23e-08	-0.03	2.48e-05	-0.02	3.79e-06	-0.02	8.99e-07	0.000174	-4.59	-4.31	-4.47	-4.41	-5.75	-5.89
cg26033526	6	32852080	PSMB9;TAP1	shore	Non-island Promoter	No	No	-0.03	2.55e-08	-0.02	0.00256	-0.03	1.53e-06	-0.03	7.96e-08	0.00108	-5.05	-4.02	-4.23	-4.53	-5.93	-5.85
cg05801388	7	66087242	ASL;ENSG00000249319	open sea	Non-island Promoter	Yes	No	-0.01	2.63e-08	-0.01	4.3e-05	-0.01	2.78e-06	-0.01	8.92e-07	4.57e-06	-4.23	-4.42	-4.77	-3.51	-5.81	-5.89
cg10264003	11	110328239	ENSG00000290078	open sea	Non-island Promoter	Yes	Yes	-0.02	2.77e-08	-0.02	3.13e-05	-0.02	7.14e-08	-0.02	1.75e-06	0.00775	-4.25	-4.65	-5.25	-3.89	-5.67	-5.79
cg16290931	19	49747296	TSKS	shore	Gene Body	Yes	No	-0.01	2.79e-08	-0.01	3.03e-05	-0.01	3.62e-08	-0.01	6.56e-08	2.78e-05	-3.95	-4.72	-5.46	-3.92	-6.15	-6.19
cg15499393	2	7748812	ENSG00000290050	open sea	Gene Body	No	No	-0.02	3.05e-08	-0.02	3.37e-06	-0.02	8e-05	-0.02	3.78e-06	0.0012	-4.03	-3.53	-4	-3.64	-5.42	-5.68
cg00495557	16	86347319	ENSG00000269826;LINC00917	open sea	Non-island Promoter	No	No	-0.02	3.6e-08	-0.02	0.00138	-0.02	4.25e-05	-0.02	2.83e-07	2.91e-05	-4.5	-2.44	-3.44	-3.65	-5.58	-5.63
cg19055984	6	15402639	JARID2	open sea	Gene Body	Yes	Yes	0.02	3.6e-08	0.02	7.07e-05	0.02	2.27e-06	0.02	4.92e-08	7.86e-07	4.03	4.3	4.4	4.41	6	6.05
cg01833052	9	78133908		open sea	Intergenic	Yes	No	-0.02	3.63e-08	-0.02	0.000144	-0.02	2.16e-06	-0.02	4.61e-07	0.000469	-2.69	-4.54	-5.37	-2.46	-5.54	-5.88
cg12318285	4	53132749	SCFD2	open sea	Gene Body	No	Yes	-0.02	3.8e-08	-0.02	3.63e-05	-0.02	2.37e-06	-0.02	1.46e-06	0.00685	-5.14	-3.38	-3.42	-4.83	-5.71	-5.77
cg05019530	22	47944045	EPIC1	open sea	Gene Body	Yes	No	-0.02	3.9e-08	-0.02	0.00438	-0.02	6.24e-07	-0.02	2.52e-07	3.07e-05	-4.3	-3.23	-3.85	-3.74	-5.91	-5.82
cg00916310	10	3810254	ENSG00000287175	open sea	Gene Body	No	No	-0.02	4.47e-08	-0.02	1.31e-07	-0.01	7.4e-05	-0.02	1.24e-07	0.00898	-3.89	-4.21	-4.67	-3.73	-5.56	-5.82
cg25022932	20	33490001	CBFA2T2	island	CpG island Promoter	Yes	No	-0.01	4.49e-08	-0.01	3.24e-06	-0.01	4.11e-06	-0.01	5.97e-06	0.0197	-4.75	-1.9	-2.33	-5.62	-5.47	-5.74
cg16371865	8	140287706	TRAPPC9	open sea	Gene Body	Yes	Yes	0	4.96e-08	0	4.77e-06	0	1.52e-05	0	5.87e-07	0.0206	-5.2	-3.27	-3.63	-3.72	-4.88	-5.81

Primary STWS EWAS: all 534 Shanghai Textile Worker Study participants (281 cotton and 253 silk workers); cotton versus silk was modeled using ComBat-corrected methylation beta values in a linear mixed model adjusted for age, sex, body mass index, smoking pack-years, years since cessation of endotoxin exposure, and EpiDISH-estimated immune-cell fractions, with nested random intercepts for Sample Plate and Sentrix ID. Categorical exposure sensitivity analysis: silk (n=253; reference), Cotton Low (n=41; average endotoxin concentration <90 EU/m3), Cotton Mid (n=115; 90 to <1700 EU/m3), and Cotton High (n=125; >=1700 EU/m3); average endotoxin concentration was cumulative endotoxin divided by years worked. Categorical and ordered-trend models used the same covariates and nested random-effects structure as the primary STWS EWAS. P trend used ordered categories silk=0, low=1, mid=2, and high=3. Subgroup sensitivity analyses show signed Z scores for the cotton-versus-silk association from linear mixed models using the primary covariates and random-effects structure; smoking pack-years were omitted in never smokers, and sex was omitted in sex-stratified models. Alternative cumulative-exposure models show signed Z scores from full-cohort ComBat-corrected linear mixed models using natural-log cumulative endotoxin or natural-log cumulative dust and the primary covariates and random-effects structure. The CellDMC neutrophil column indicates whether the cotton-versus-silk neutrophil-specific interaction met nominal P<0.05 in the Sample Plate-adjusted CellDMC model, which included EpiDISH-estimated immune-cell fractions and adjusted for age, sex, height, body mass index, smoking pack-years, years since exposure cessation, and Sample Plate. Abbreviations: CpG=cytosine-phosphate-guanine; EpiDISH=Epigenetic Dissection of Intra-Sample Heterogeneity; EU=endotoxin unit; STWS=Shanghai Textile Worker Study; Z=standard score.

medRxiv preprint doi: https://doi.org/10.64898/2026.09.29.26364351; this version posted October 1, 2026. The copyright holder for this preprint (which was not certified by peer review) is the author/funder, who has granted medRxiv a license to display the preprint in perpetuity. This article is a US Government work. It is not subject to copyright under 17 USC 105 and is also made available for use under a CC0 license.

Supplemental Table 5. STWS Primary and SWHS Validation Results for 36 Differentially Methylated Positions

CpG	STWS primary			SWHS primary validation			SWHS sensitivity validation		
	Beta	SE	P value	Beta	SE	P value	Beta	SE	P value
cg00495557	-0.0234	0.0042	3.60E-08	-0.0052	0.0043	0.226	-0.0063	0.0043	0.141
cg00916310	-0.0187	0.0032	4.47E-08	-0.0044	0.0026	0.097	-0.0020	0.0027	0.441
cg01030629	-0.0058	0.0010	1.51E-08	/	/	/	/	/	/
cg01778030	-0.0201	0.0032	5.57E-09	-0.0031	0.0024	0.196	-0.0023	0.0024	0.351
cg01833052	-0.0191	0.0033	3.63E-08	-0.0046	0.0032	0.153	-0.0024	0.0034	0.479
cg04155673	-0.0040	0.0006	5.63E-09	0.0060	0.0028	0.032	0.0065	0.0029	0.023
cg04522575	-0.0239	0.0040	1.95E-08	-0.0022	0.0035	0.527	0.0028	0.0035	0.415
cg05019530	-0.0223	0.0038	3.90E-08	-0.0074	0.0036	0.042	-0.0053	0.0037	0.148
cg05408620	-0.0194	0.0034	1.32E-08	/	/	/	/	/	/
cg05801388	-0.0078	0.0013	2.63E-08	-0.0013	0.0010	0.217	-0.0004	0.0011	0.726
cg08009902	-0.0130	0.0020	6.05E-10	0.0038	0.0031	0.226	0.0059	0.0032	0.065
cg08129102	-0.0059	0.0010	6.53E-09	0.0005	0.0023	0.831	-0.0001	0.0024	0.978
cg09660738	-0.0152	0.0024	4.15E-09	-0.0030	0.0041	0.456	-0.0037	0.0041	0.374
cg10243623	-0.0249	0.0042	2.23E-08	-0.0066	0.0038	0.084	-0.0033	0.0038	0.382
cg10264003	-0.0184	0.0031	2.77E-08	-0.0022	0.0024	0.368	-0.0014	0.0025	0.564
cg10793507	-0.0095	0.0015	5.54E-09	-0.0004	0.0042	0.926	0.0019	0.0044	0.654
cg11248361	-0.0131	0.0019	1.75E-10	-0.0018	0.0029	0.551	-0.0022	0.0030	0.456
cg11795976	-0.0160	0.0028	1.92E-08	0.0005	0.0040	0.909	0.0018	0.0042	0.662
cg11800040	-0.0101	0.0017	1.01E-08	/	/	/	/	/	/
cg11869499	-0.0146	0.0023	1.87E-09	-0.0044	0.0022	0.041	-0.0002	0.0022	0.929
cg12318285	-0.0220	0.0038	3.80E-08	/	/	/	/	/	/
cg15499393	-0.0172	0.0031	3.05E-08	-0.0028	0.0038	0.457	0.0006	0.0039	0.878
cg16039157	-0.0215	0.0035	8.38E-09	0.0006	0.0037	0.859	0.0048	0.0036	0.184
cg16290931	-0.0074	0.0012	2.79E-08	-0.0016	0.0008	0.044	-0.0015	0.0008	0.062
cg16371865	-0.0033	0.0006	4.96E-08	0.0005	0.0016	0.770	0.0006	0.0016	0.696
cg19055984	0.0227	0.0038	3.60E-08	0.0012	0.0034	0.716	0.0013	0.0034	0.707
cg19777190	-0.0219	0.0038	1.52E-08	0.0003	0.0032	0.928	-0.0008	0.0032	0.810
cg19925755	-0.0033	0.0006	1.07E-08	0.0011	0.0012	0.356	0.0004	0.0012	0.741
cg22740441	-0.0225	0.0031	4.76E-11	-0.0001	0.0033	0.973	0.0058	0.0034	0.090
cg22766862	-0.0077	0.0013	1.15E-08	/	/	/	/	/	/
cg25022932	-0.0059	0.0010	4.49E-08	0.0004	0.0009	0.622	0.0009	0.0009	0.310
cg26033526	-0.0277	0.0047	2.55E-08	-0.0110	0.0044	0.013	-0.0049	0.0045	0.273
cg26277709	-0.0129	0.0020	1.12E-09	0.0007	0.0004	0.106	0.0005	0.0005	0.276
cg26491213	-0.0064	0.0010	1.80E-09	0.0007	0.0006	0.245	0.0007	0.0006	0.213
cg26947969	-0.0049	0.0008	1.34E-08	0.0010	0.0027	0.719	0.0019	0.0027	0.483
cg27039003	-0.0251	0.0042	2.10E-08	/	/	/	/	/	/

STWS primary: ComBat-corrected DNAm beta values were compared between cotton and silk workers using linear mixed regression adjusted for age, sex, body mass index, smoking pack-years, years since cessation of endotoxin exposure, and EpiDISH-estimated immune-cell fractions, with nested random intercepts for Sample Plate and Sentrix ID.

SWHS primary validation: women with high ordinal exposure-intensity rank were compared with women in the combined low/medium group using linear mixed regression adjusted for age, body mass index, education, case-control status, EpiDISH-estimated immune-cell fractions, and Sample Well, with a random intercept for matched case-control set.

SWHS sensitivity validation: the same high versus combined low/medium comparison used the same covariates and random intercept and was weighted using inverse-probability weights for case-control status.

Abbreviations: BMI=body mass index; CpG=cytosine-phosphate-guanine; DMP=differentially methylated position; EpiDISH=Epigenetic Dissection of Intra-Sample Heterogeneity; IPW=inverse-probability weighting; SE=standard error; STWS=Shanghai Textile Worker Study; SWHS=Shanghai Women's Health Study. / indicates a CpG unavailable in SWHS.

medRxiv preprint doi: https://doi.org/10.64898/2026.09.29.26364351; this version posted October 1, 2026. The copyright holder for this preprint
(which was not certified by peer review) is the author/funder, who has granted medRxiv a license to display the preprint in perpetuity.
This article is a US Government work. It is not subject to copyright under 17 USC 105 and is also made available
for use under a CC0 license.

Supplemental Table 6. Gene Set Enrichment Analysis leading-edge genes from collapsed representative pathways

Gene label	Entrez ID	Gene name	Number of pathways	Sources	Average rank	Average tags (%)	Average list (%)	Average signal (%)	Maximum normalized enrichment score	Minimum q-value
ITGB1	3688	integrin subunit beta 1	34	GO:BP; GO:CC; GO:MF; KEGG; REAC	6321.4	34.4	20.8	27.4	1.58	2.98e-07
PIK3CA	5290	phosphatidylinositol-4,5-bisphosphate 3-kinase catalytic subunit alpha	32	GO:BP; GO:CC; GO:MF; KEGG; REAC	6069.8	35.3	20	28.4	1.56	3.66e-07
CAV1	857	caveolin 1	31	GO:BP; GO:CC; GO:MF; KEGG; REAC	6379.6	34.9	21	27.7	1.56	1.13e-07
DLG1	1739	discs large MAGUK scaffold protein 1	29	GO:BP; GO:CC; KEGG; REAC	5816.8	34.1	19.2	27.7	1.56	2.98e-07
MAPK1	5594	mitogen-activated protein kinase 1	28	GO:BP; GO:CC; GO:MF; KEGG; REAC	6005.1	34	19.8	27.4	1.55	3.66e-07
TNF	7124	tumor necrosis factor	28	GO:BP; KEGG	6917	34.5	22.8	26.6	1.43	1.17e-05
MTOR	2475	mechanistic target of rapamycin kinase	27	GO:BP; GO:CC; GO:MF; KEGG; REAC	7046.6	35.2	23.2	27.1	1.49	3.66e-07
PSEN1	5663	presenilin 1	25	GO:BP; GO:CC; KEGG; REAC	6750	33.4	22.2	26	1.41	1.13e-07
PTK2B	2185	protein tyrosine kinase 2 beta	25	GO:BP; GO:CC; GO:MF; KEGG; REAC	6760.1	34.3	22.2	26.6	1.43	1.13e-07
RDX	5962	radixin	25	GO:BP; GO:CC; GO:MF; KEGG; REAC	5721.3	34.6	18.8	28.3	1.59	1.13e-07
CYFIP1	23191	cytoplasmic FMR1 interacting protein 1	25	GO:BP; GO:CC; GO:MF; REAC	6555.8	34.8	21.5	27.4	1.51	7.84e-07
EZR	7430	ezrin	24	GO:BP; GO:CC; GO:MF; KEGG; REAC	5535	34.5	18.2	28.4	1.59	1.13e-07
PAFAH1B1	5048	platelet activating factor acetylhydrolase 1b regulatory subunit 1	24	GO:BP; GO:CC; GO:MF	6266.4	33.5	20.5	26.8	1.54	1.13e-07
ITGB3	3690	integrin subunit beta 3	24	GO:BP; GO:CC; KEGG; REAC	6860.8	33.2	22.6	25.8	1.36	3.66e-07
MIR21	406991	microRNA 21	24	GO:BP; KEGG	6478.8	33.9	21.3	26.7	1.61	1.62e-06
BRAF	673	B-Raf proto-oncogene, serine/threonine kinase	23	GO:BP; GO:MF; KEGG; REAC	6670.8	35.3	21.9	27.7	1.63	3.66e-07
RAC1	5879	Rac family small GTPase 1	23	GO:BP; GO:CC; KEGG; REAC	6996.5	36	23	28	1.49	3.66e-07
PAK1	5058	p21 (RAC1) activated kinase 1	23	GO:BP; GO:CC; GO:MF; KEGG; REAC	6306.7	34.3	20.7	27.3	1.56	7.84e-07
CLASP2	23122	cytoplasmic linker associated protein 2	22	GO:BP; GO:CC; GO:MF	6844.4	33.7	22.5	26.2	1.41	1.13e-07
EPS8	2059	EGFR pathway substrate 8, signaling adaptor	22	GO:BP; GO:CC; GO:MF; REAC	6337.9	34.8	20.9	27.6	1.51	1.13e-07
FYN	2534	FYN proto-oncogene, Src family tyrosine kinase	22	GO:BP; GO:CC; GO:MF; KEGG; REAC	6423.7	35.7	21.1	28.3	1.49	2.98e-07
PIK3CB	5291	phosphatidylinositol-4,5-bisphosphate 3-kinase catalytic subunit beta	22	GO:BP; KEGG; REAC	6084.1	34.5	20	27.6	1.47	3.66e-07
DAG1	1605	dystroglycan 1	22	GO:BP; GO:CC; GO:MF	6855.1	34.5	22.5	26.8	1.41	4.89e-06
ANK2	287	ankyrin 2	22	GO:BP; GO:CC; KEGG	5411.1	33.1	17.9	27.2	1.56	8.20e-06
ACTB	60	actin beta	21	GO:BP; GO:CC; KEGG; REAC	7029.7	35.4	23	27.5	1.39	3.66e-07
LYN	4067	LYN proto-oncogene, Src family tyrosine kinase	21	GO:BP; GO:CC; GO:MF; KEGG; REAC	7337.3	36	24.1	27.6	1.49	3.66e-07
BCL2	596	BCL2 apoptosis regulator	21	GO:BP; KEGG	6648.4	32.6	21.9	25.3	1.39	1.51e-04
GATA3	2625	GATA binding protein 3	21	GO:BP	6493.9	34	21.3	26.7	1.69	1.51e-04
CDH2	1000	cadherin 2	20	GO:BP; GO:CC; KEGG	6259.6	34	20.6	27	1.56	1.13e-07
KRAS	3845	KRAS proto-oncogene, GTPase	20	GO:BP; GO:CC; KEGG; REAC	6385.9	33.2	21	26.2	1.47	3.66e-07
PIK3R1	5295	phosphoinositide-3-kinase regulatory subunit 1	20	GO:BP; GO:CC; KEGG; REAC	7147.8	36.8	23.6	28.5	1.49	3.66e-07
KCNQ1	3784	potassium voltage-gated channel subfamily Q member 1	20	GO:BP; GO:CC; GO:MF; KEGG; REAC	6243.9	35	20.6	27.7	1.56	1.62e-05
KANK1	23189	KN motif and ankyrin repeat domains 1	19	GO:BP; GO:CC; REAC	6165.4	35.8	20.3	28.7	1.69	1.13e-07
SOS1	6654	SOS Ras/Rac guanine nucleotide exchange factor 1	19	GO:BP; GO:CC; GO:MF; KEGG; REAC	6153.2	33.3	20.3	26.5	1.43	1.21e-07
TIAM1	7074	TIAM Rac1 associated GEF 1	19	GO:BP; GO:CC; GO:MF; KEGG; REAC	6442.4	33.4	21.2	26.4	1.38	1.21e-07
MDM2	4193	MDM2 proto-oncogene	19	GO:BP; GO:CC; GO:MF; KEGG; REAC	6596.2	32.6	21.7	25.4	1.59	2.98e-07
ROCK2	9475	Rho associated coiled-coil containing protein kinase 2	19	GO:BP; GO:MF; KEGG; REAC	6026.2	35.2	19.8	28.3	1.69	7.84e-07
IQGAP1	8826	IQ motif containing GTPase activating protein 1	18	GO:BP; GO:CC; GO:MF; KEGG; REAC	6356.4	34	20.9	27.1	1.43	1.13e-07
BAIAP2	10458	BAR/IMD domain containing adaptor protein 2	18	GO:BP; GO:CC; KEGG; REAC	6789	34.7	22.3	27.1	1.38	2.98e-07
NLGN1	22871	neuroligin 1	18	GO:BP; GO:CC; KEGG; REAC	6600.6	36.5	21.7	28.7	1.51	2.98e-07
PIK3R3	8503	phosphoinositide-3-kinase regulatory subunit 3	18	GO:BP; KEGG; REAC	6186.7	34.8	20.4	27.8	1.49	3.66e-07
CTNNB1	1499	catenin beta 1	18	GO:BP; GO:CC; KEGG	7562.1	35.9	24.9	27.2	1.36	8.20e-06
CORO1A	11151	coronin 1A	17	GO:BP; GO:CC; GO:MF	6562.8	32.8	21.6	25.8	1.41	1.13e-07
PTK2	5747	protein tyrosine kinase 2	17	GO:BP; GO:CC; GO:MF; KEGG; REAC	6310.5	33.4	20.7	26.4	1.41	1.13e-07
WDPCP	51057	WD repeat containing planar cell polarity effector	17	GO:BP; GO:CC; GO:MF	6253.4	34.8	20.5	27.8	1.69	1.13e-07
RAP1A	5906	RAP1A, member of RAS oncogene family	17	GO:BP; GO:CC; GO:MF; KEGG; REAC	6516.5	33.9	21.4	26.7	1.42	1.21e-07
PDE4B	5142	phosphodiesterase 4B	17	GO:BP; GO:CC; GO:MF; KEGG	5626.8	34.3	18.5	28	1.61	2.98e-07
RAF1	5894	Raf-1 proto-oncogene, serine/threonine kinase	17	GO:BP; GO:MF; KEGG; REAC	5940.6	32.8	19.6	26.4	1.47	3.66e-07
LRRK2	120892	leucine rich repeat kinase 2	17	GO:BP; GO:CC; GO:MF	7700.2	36.5	25.4	27.4	1.36	2.37e-06
NRXN1	9378	neurexin 1	17	GO:BP; GO:CC; REAC	5957.1	33.1	19.5	26.6	1.56	5.11e-05
CLASP1	23332	cytoplasmic linker associated protein 1	16	GO:BP; GO:CC; GO:MF	7227.2	35.2	23.8	27	1.41	1.13e-07
NF1	4763	neurofibromin 1	16	GO:BP; GO:MF; REAC	6786.3	33.4	22.4	26.1	1.38	1.21e-07
GPER1	2852	G protein-coupled estrogen receptor 1	16	GO:BP; GO:CC	6561.6	31.8	21.6	24.9	1.38	2.98e-07

medRxiv preprint doi: https://doi.org/10.64898/2026.09.29.26364351; this version posted October 1, 2026. The copyright holder for this preprint (which was not certified by peer review) is the author/funder, who has granted medRxiv a license to display the preprint in perpetuity.
This article is a US Government work. It is not subject to copyright under 17 USC 105 and is also made available for use under a CC0 license.

Gene label	Entrez ID	Gene name	Number of pathways	Sources	Average rank	Average tags (%)	Average list (%)	Average signal (%)	Maximum normalized enrichment score	Minimum q-value
PTPN6	5777	protein tyrosine phosphatase non-receptor type 6	16	GO:BP; GO:CC; KEGG; REAC	6895.8	36.5	22.8	28.3	1.49	3.66e-07
JUP	3728	junction plakoglobin	16	GO:BP; GO:CC; REAC	6469.1	35.1	21.3	27.9	1.56	7.84e-07
ADCY10	55811	adenylate cyclase 10	16	GO:BP; GO:CC; KEGG	6199.4	33.6	20.3	26.9	1.51	2.71e-05
SRF	6722	serum response factor	16	GO:BP	7305.4	34.2	24.1	26	1.37	1.20e-04
PRKCB	5579	protein kinase C beta	15	GO:BP; GO:CC; GO:MF; KEGG; REAC	6447.5	34.9	21.2	27.5	1.41	1.13e-07
TRAF6	7189	TNF receptor associated factor 6	15	GO:BP; GO:CC; GO:MF; KEGG; REAC	7489	36.5	24.6	27.7	1.41	1.13e-07
CACNA1C	775	calcium voltage-gated channel subunit alpha1 C	15	GO:BP; GO:CC; KEGG	5058.1	32.1	16.7	26.7	1.61	2.98e-07
CAMK2A	815	calcium/calmodulin dependent protein kinase II alpha	15	GO:BP; GO:CC; GO:MF; KEGG; REAC	6552.9	33.7	21.5	26.3	1.38	2.98e-07
CAMK2B	816	calcium/calmodulin dependent protein kinase II beta	15	GO:BP; GO:CC; GO:MF; KEGG; REAC	6495.9	33.8	21.3	26.5	1.36	2.98e-07
EPHA4	2043	EPH receptor A4	15	GO:BP; GO:CC	6925.5	34.1	22.7	26.5	1.38	2.98e-07
AKT3	10000	AKT serine/threonine kinase 3	15	GO:BP; GO:MF; KEGG; REAC	5508.9	33.4	18.3	27.3	1.59	3.66e-07
APC	324	APC regulator of WNT signaling pathway	15	GO:BP; GO:CC; GO:MF; KEGG; REAC	6971.4	34.9	22.9	27	1.39	3.66e-07
CREB1	1385	cAMP responsive element binding protein 1	15	GO:BP; KEGG; REAC	6232.1	32.3	20.4	25.6	1.59	3.66e-07
FZD5	7855	frizzled class receptor 5	15	GO:BP; GO:CC; KEGG; REAC	6854.7	33.9	22.7	26.4	1.36	3.66e-07
PTEN	5728	phosphatase and tensin homolog	15	GO:BP; GO:CC; KEGG; REAC	7422.7	36.2	24.3	27.6	1.38	3.66e-07
CYRIB	51571	CYFIP related Rac1 interactor B	15	GO:BP; GO:MF	6815.3	36	22.5	28.2	1.69	1.62e-06
PTPRC	5788	protein tyrosine phosphatase receptor type C	15	GO:BP; GO:CC; KEGG	7015.1	34.3	23.1	26.3	1.43	1.17e-05
PDE4D	5144	phosphodiesterase 4D	15	GO:BP; GO:CC; KEGG	4824.1	32.5	15.9	27.3	1.61	2.71e-05
DLC1	10395	DLC1 Rho GTPase activating protein	14	GO:BP; GO:CC; GO:MF; REAC	6299.2	32.9	20.6	26.1	1.41	1.13e-07
SH3BP1	23616	SH3 domain binding protein 1	14	GO:BP; GO:CC; GO:MF; REAC	6384.1	34.4	21	27.4	1.51	1.13e-07
EGF	1950	epidermal growth factor	14	GO:BP; GO:CC; GO:MF; KEGG; REAC	6824.2	36.1	22.4	28.4	1.59	1.21e-07
TSC2	7249	TSC complex subunit 2	14	GO:BP; GO:CC; GO:MF; KEGG; REAC	6702.6	34.1	22.1	26.5	1.59	1.21e-07
AKAP9	10142	A-kinase anchoring protein 9	14	GO:BP; GO:CC; REAC	5582.5	35.4	18.4	28.9	1.56	2.98e-07
DISC1	27185	DISC1 scaffold protein	14	GO:BP; GO:CC	7305.6	36.1	24	27.5	1.37	2.98e-07
HSPA8	3312	heat shock protein family A (Hsp70) member 8	14	GO:BP; GO:CC; REAC	7328	36.1	24	27.6	1.39	2.98e-07
PRKCG	5582	protein kinase C gamma	14	GO:BP; GO:CC; GO:MF; KEGG; REAC	6471.6	33.9	21.4	26.6	1.36	2.98e-07
AP3B1	8546	adaptor related protein complex 3 subunit beta 1	14	GO:BP; GO:CC; KEGG; REAC	7538.7	36.6	24.8	27.9	1.50	3.66e-07
LCK	3932	LCK proto-oncogene, Src family tyrosine kinase	14	GO:BP; KEGG; REAC	6399.8	36.4	21.1	28.7	1.49	3.66e-07
PLD1	5337	phospholipase D1	14	GO:BP; GO:CC; GO:MF; KEGG; REAC	6581.2	34.6	21.6	27.4	1.47	7.84e-07
SMAD7	4092	SMAD family member 7	14	GO:BP; GO:CC; REAC	7358.5	35.3	24.1	26.8	1.32	2.71e-05
DST	667	dystonin	13	GO:BP; GO:CC; GO:MF; REAC	6630.7	34.4	21.8	27.2	1.41	1.13e-07
PHLDB2	90102	pleckstrin homology like domain family B member 2	13	GO:BP; GO:CC	6815.5	33.7	22.4	26.3	1.41	1.13e-07
PRKD1	5587	protein kinase D1	13	GO:BP; GO:CC; GO:MF	7066.8	34	23.2	26.2	1.41	1.13e-07
RIMS1	22999	regulating synaptic membrane exocytosis 1	13	GO:BP; GO:CC; GO:MF; KEGG; REAC	6426.7	33.8	21	26.6	1.41	1.13e-07
STXBP1	6812	syntaxin binding protein 1	13	GO:BP; GO:CC; REAC	6711.2	34.3	22	26.8	1.47	1.13e-07
WASL	8976	WASP like actin nucleation promoting factor	13	GO:BP; GO:CC; GO:MF; KEGG; REAC	6353.6	34.5	20.9	27.5	1.41	1.13e-07
CCDC88A	55704	coiled-coil domain containing 88A	13	GO:BP; GO:CC; GO:MF; REAC	6706.8	33.5	22.2	26.2	1.34	1.21e-07
PLCB1	23236	phospholipase C beta 1	13	GO:BP; GO:MF; KEGG; REAC	7042.8	33.9	23.1	26.1	1.34	1.21e-07
SLIT2	9353	slit guidance ligand 2	13	GO:BP; GO:MF; KEGG	6334.9	32.7	20.8	25.8	1.39	1.21e-07
VAV3	10451	vav guanine nucleotide exchange factor 3	13	GO:BP; GO:MF; KEGG; REAC	5980.5	34.2	19.7	27.5	1.43	1.21e-07
SYT1	6857	synaptotagmin 1	13	GO:BP; GO:CC; GO:MF; REAC	6610.2	34.9	21.6	27.3	1.47	2.98e-07
ADAM17	6868	ADAM metallopeptidase domain 17	13	GO:BP; GO:CC; REAC	6923.8	31.7	22.8	24.8	1.30	3.66e-07
FN1	2335	fibronectin 1	13	GO:BP; GO:CC; KEGG; REAC	6554.5	33.9	21.5	26.5	1.41	3.66e-07
RB1	5925	RB transcriptional corepressor 1	13	GO:BP; KEGG; REAC	6592.5	32.3	21.7	25.2	1.47	3.66e-07
ABI2	10152	abl interactor 2	13	GO:BP; GO:CC; GO:MF; REAC	6906.3	35.3	22.8	27.5	1.38	7.84e-07
CD2AP	23607	CD2 associated protein	13	GO:BP; GO:CC; GO:MF; KEGG	6922.5	34.4	22.8	26.8	1.38	1.62e-06
RIPOR2	9750	RHO family interacting cell polarization regulator 2	13	GO:BP; GO:CC; REAC	6277.6	34.5	20.8	27.6	1.69	1.62e-06
CD44	960	CD44 molecule (IN blood group)	13	GO:BP; GO:CC; KEGG	6637.5	31.7	21.8	24.7	1.36	4.89e-06
EPB41L3	23136	erythrocyte membrane protein band 4.1 like 3	13	GO:BP; GO:CC; GO:MF; KEGG; REAC	5789.3	33.4	19.2	27.1	1.47	4.89e-06
MACF1	23499	microtubule actin crosslinking factor 1	13	GO:BP; GO:CC; GO:MF	6350.5	32.4	20.8	25.5	1.41	4.89e-06
PRKCI	5584	protein kinase C iota	13	GO:BP; GO:CC; GO:MF; KEGG	6427.2	33.9	21.2	26.8	1.51	4.89e-06
MAPK14	1432	mitogen-activated protein kinase 14	13	GO:BP; GO:MF; KEGG	6654.5	34.4	21.9	26.8	1.43	8.20e-06
MALT1	10892	MALT1 paracaspase	13	GO:BP; GO:MF; KEGG; REAC	7345.9	37.3	24.2	28.3	1.43	1.17e-05
PFN2	5217	profilin 2	13	GO:BP; GO:MF	6870.8	36	22.5	28.2	1.51	1.62e-05
CD3E	916	CD3 epsilon subunit of T-cell receptor complex	13	GO:BP; GO:CC; REAC	7224.6	38.5	23.8	29.6	1.49	2.71e-05

medRxiv preprint doi: https://doi.org/10.64898/2026.09.29.26364351; this version posted October 1, 2026. The copyright holder for this preprint
(which was not certified by peer review) is the author/funder, who has granted medRxiv a license to display the preprint in perpetuity.
This article is a US Government work. It is not subject to copyright under 17 USC 105 and is also made available
for use under a CC0 license.

Gene label	Entrez ID	Gene name	Number of pathways	Sources	Average rank	Average tags (%)	Average list (%)	Average signal (%)	Maximum normalized enrichment score	Minimum q-value
AP1G1	164	adaptor related protein complex 1 subunit gamma 1	13	GO:BP; GO:CC; GO:MF; KEGG	7456.5	37.3	24.5	28.4	1.50	5.11e-05
OPA1	4976	OPA1 mitochondrial dynamin like GTPase	13	GO:BP; GO:CC; GO:MF	7677.5	35.9	25.2	27	1.30	1.96e-04
INHBA	3624	inhibin subunit beta A	13	GO:BP; REAC	7230.5	33.5	23.8	25.6	1.37	6.59e-04
SIRT1	23411	sirtuin 1	13	GO:BP	7216	32.8	23.7	24.8	1.28	4.74e-03
ADD3	120	adducin 3	12	GO:BP; GO:CC; GO:MF; REAC	6696.2	35.2	22	27.7	1.41	1.13e-07
MYO6	4646	myosin VI	12	GO:BP; GO:CC; GO:MF; REAC	6392.9	32.8	21	26	1.41	1.13e-07
PARD3	56288	par-3 family cell polarity regulator	12	GO:BP; GO:CC; GO:MF; KEGG; REAC	5650	31.8	18.6	26	1.51	1.13e-07
SPTBN2	6712	spectrin beta, non-erythrocytic 2	12	GO:BP; GO:CC; GO:MF; KEGG; REAC	6301	32.8	20.7	26.2	1.41	1.13e-07
DOCK10	55619	dedicator of cytokinesis 10	12	GO:BP; GO:CC; GO:MF; REAC	7005.1	35.8	23	27.6	1.38	1.21e-07
RAPGEF2	9693	Rap guanine nucleotide exchange factor 2	12	GO:BP; GO:CC; GO:MF; KEGG; REAC	6408.3	33.8	21.1	26.8	1.42	1.21e-07
DLG2	1740	discs large MAGUK scaffold protein 2	12	GO:BP; GO:CC; KEGG; REAC	6463.3	35.2	21.2	27.9	1.51	2.98e-07
P2RY1	5028	purinergic receptor P2Y1	12	GO:BP; GO:CC	6338.4	32.2	20.8	25.5	1.36	2.98e-07
PPFIA2	8499	PTPRF interacting protein alpha 2	12	GO:BP; GO:CC; KEGG; REAC	7191.2	35.9	23.6	27.5	1.38	2.98e-07
SHANK2	22941	SH3 and multiple ankyrin repeat domains 2	12	GO:BP; GO:CC; REAC	6044.2	33.6	19.8	26.9	1.47	2.98e-07
VPS35	55737	VPS35 retromer complex component	12	GO:BP; GO:CC; KEGG	7455.9	35.1	24.5	26.8	1.36	2.98e-07
FGF10	2255	fibroblast growth factor 10	12	GO:BP; REAC	7266	33.9	23.9	26.1	1.38	3.66e-07
KIF5B	3799	kinesin family member 5B	12	GO:BP; GO:CC; GO:MF; REAC	6778.4	34.8	22.2	27.3	1.53	3.66e-07
MET	4233	MET proto-oncogene, receptor tyrosine kinase	12	GO:BP; GO:CC; KEGG; REAC	6948	34.9	22.9	27.1	1.39	3.66e-07
NRG1	3084	neuregulin 1	12	GO:BP; GO:CC; REAC	7137.7	34	23.3	26.2	1.36	3.66e-07
TMOD3	29766	tropomodulin 3	12	GO:BP; GO:CC; GO:MF; REAC	6721.2	33.4	22.1	26.4	1.39	7.84e-07
WASF2	10163	WASP family member 2	12	GO:BP; GO:CC; GO:MF; REAC	6578.2	34.4	21.8	27.2	1.39	7.84e-07
ADORA1	134	adenosine A1 receptor	12	GO:BP; GO:CC; KEGG	7010.9	35.6	23.1	27.6	1.38	4.89e-06
ARPC2	10109	actin related protein 2/3 complex subunit 2	12	GO:BP; GO:CC; GO:MF; KEGG; REAC	7223	34.8	23.8	26.8	1.37	4.89e-06
RAB5A	5868	RAB5A, member RAS oncogene family	12	GO:BP; GO:CC; REAC	6984.1	34.1	23	26.5	1.36	4.89e-06
VIL1	7429	villin 1	12	GO:BP; GO:CC; GO:MF	6215.9	35	20.5	28.1	1.43	4.89e-06
ANK3	288	ankyrin 3	12	GO:BP; GO:CC; KEGG	5755.9	33.6	19	27.2	1.56	8.20e-06
AKAP6	9472	A-kinase anchoring protein 6	12	GO:BP; GO:CC	4660.1	32.4	15.3	27.4	1.56	2.71e-05
CACNB4	785	calcium voltage-gated channel auxiliary subunit beta 4	12	GO:BP; GO:CC; REAC	6640.7	32.9	21.8	25.8	1.36	5.11e-05
RIPK2	8767	receptor interacting serine/threonine kinase 2	12	GO:BP; GO:MF; REAC	7355.8	37.2	24.2	28.2	1.36	2.04e-04
CD74	972	CD74 molecule	12	GO:BP; GO:CC	8385.5	37.2	27.6	27.2	1.33	8.19e-04
FGFR2	2263	fibroblast growth factor receptor 2	11	GO:BP; GO:CC; REAC	6719.3	32.5	22	25.5	1.41	1.13e-07
DOCK7	85440	dedicator of cytokinesis 7	11	GO:BP; GO:CC; GO:MF; REAC	6655	33.6	21.8	26.4	1.38	1.21e-07
MYO9A	4649	myosin IXA	11	GO:BP; GO:CC; GO:MF; REAC	6355.3	33.8	20.9	26.9	1.51	1.21e-07
ABI3	51225	ABI family member 3	11	GO:BP; GO:CC; GO:MF	6120.1	34.9	20.2	27.8	1.51	2.98e-07
ATP2B2	491	ATPase plasma membrane Ca2+ transporting 2	11	GO:BP; GO:CC; KEGG; REAC	6321.9	33.4	20.8	26.4	1.39	2.98e-07
SHANK3	85358	SH3 and multiple ankyrin repeat domains 3	11	GO:BP; GO:CC; GO:MF	6801.3	34.5	22.3	26.8	1.39	2.98e-07
SLC8A1	6546	solute carrier family 8 member A1	11	GO:BP; GO:CC	5534.9	31.1	18.3	25.6	1.56	2.98e-07
CBL	867	Cbl proto-oncogene	11	GO:BP; GO:CC; GO:MF; KEGG; REAC	6485.9	33.2	21.5	26.1	1.38	3.66e-07
CD86	942	CD86 molecule	11	GO:BP; KEGG; REAC	7324.5	38.6	24.1	29.5	1.49	3.66e-07
KITLG	4254	KIT ligand	11	GO:BP; GO:CC; REAC	7095.7	34.8	23.4	26.9	1.38	3.66e-07
NCSTN	23385	nicastrin	11	GO:BP; GO:CC; REAC	6557.2	32.5	21.5	25.7	1.34	3.66e-07
PPP1CC	5501	protein phosphatase 1 catalytic subunit gamma	11	GO:BP; GO:CC; KEGG; REAC	6749.7	34.5	22.1	26.6	1.38	3.66e-07
RICTOR	253260	RPTOR independent companion of MTOR complex 2	11	GO:BP; REAC	6113.6	35.5	20.2	28.6	1.69	3.66e-07
CFTR	1080	CF transmembrane conductance regulator	11	GO:BP; GO:CC; KEGG; REAC	6579.1	33.2	21.5	26.2	1.42	7.84e-07
LPAR1	1902	lysophosphatidic acid receptor 1	11	GO:BP; GO:CC	5953.5	34.1	19.5	27.3	1.42	1.62e-06
NTRK1	4914	neurotrophic receptor tyrosine kinase 1	11	GO:BP; GO:CC	7311.2	35.3	24.2	27	1.38	1.62e-06
SHTN1	57698	shootin 1	11	GO:BP; GO:CC; GO:MF	6328.9	32.7	20.8	26	1.34	2.37e-06
MYO5A	4644	myosin VA	11	GO:BP; GO:CC; GO:MF; REAC	6351.3	33.7	21	26.8	1.41	4.89e-06
PRKCA	5578	protein kinase C alpha	11	GO:BP; GO:MF; KEGG; REAC	7622.2	36.2	25.1	27.3	1.33	8.20e-06
ATM	472	ATM serine/threonine kinase	11	GO:BP; GO:CC; GO:MF; KEGG	7503	36.1	24.6	27.1	1.29	1.17e-05
ATP6V0A4	50617	ATPase H+ transporting V0 subunit a4	11	GO:BP; GO:CC; KEGG	6482.4	33.6	21.4	26.5	1.39	1.17e-05
FASLG	356	Fas ligand	11	GO:BP; GO:CC; KEGG	4839	28.2	16	23.5	1.53	1.17e-05
NFKB1	4790	nuclear factor kappa B subunit 1	11	GO:BP; KEGG; REAC	6386.4	34.9	20.9	27.5	1.47	1.17e-05
FMN1	342184	formin 1	11	GO:BP; GO:CC; GO:MF	7083.5	33.4	23.4	25.9	1.27	2.71e-05
SPN	6693	sialophorin	11	GO:BP; GO:CC	7358.1	38.6	24.2	29.4	1.42	8.38e-05

medRxiv preprint doi: https://doi.org/10.64898/2026.09.29.26364351; this version posted October 1, 2026. The copyright holder for this preprint (which was not certified by peer review) is the author/funder, who has granted medRxiv a license to display the preprint in perpetuity.
This article is a US Government work. It is not subject to copyright under 17 USC 105 and is also made available for use under a CC0 license.

Gene label	Entrez ID	Gene name	Number of pathways	Sources	Average rank	Average tags (%)	Average list (%)	Average signal (%)	Maximum normalized enrichment score	Minimum q-value
ACE	1636	angiotensin I converting enzyme	11	GO:BP; GO:CC; GO:MF	7713	36	25.4	27.1	1.36	1.20e-04
KCNJ8	3764	potassium inwardly rectifying channel subfamily J member 8	11	GO:BP; GO:CC; REAC	7162.8	33.7	23.5	26	1.34	1.20e-04
TRIM32	22954	tripartite motif containing 32	11	GO:BP; GO:CC; GO:MF; REAC	7206.1	33.3	23.7	25.5	1.27	1.51e-04
DAB2	1601	DAB adaptor protein 2	11	GO:BP; GO:CC	7647.3	34.3	25	25.8	1.33	4.37e-04
MSH2	4436	mutS homolog 2	11	GO:BP; GO:MF	6565.1	34.8	21.5	27.3	1.62	6.59e-04
LAMP1	3916	lysosomal associated membrane protein 1	11	GO:BP; GO:CC; KEGG	7236.7	35.5	23.7	27.1	1.41	8.19e-04
CHD7	55636	chromodomain helicase DNA binding protein 7	11	GO:BP; GO:MF	5356.8	29.7	17.6	24.3	1.65	2.32e-03
HMGB1	3146	high mobility group box 1	11	GO:BP; GO:CC	7892.5	37.3	25.9	27.6	1.30	2.48e-03
IL12RB1	3594	interleukin 12 receptor subunit beta 1	11	GO:BP; GO:CC	7730.9	36.6	25.5	27.4	1.30	2.48e-03
COBL	23242	cordon-bleu WH2 repeat protein	10	GO:BP; GO:CC; GO:MF	6233.2	34.1	20.5	27.3	1.51	1.13e-07
IQGAP2	10788	IQ motif containing GTPase activating protein 2	10	GO:BP; GO:CC; GO:MF; REAC	6424.9	35.5	21.1	28.1	1.43	1.13e-07
ITPR2	3709	inositol 1,4,5-trisphosphate receptor type 2	10	GO:BP; GO:CC; GO:MF; KEGG; REAC	6495.6	35.4	21.4	27.8	1.42	1.13e-07
NEDD9	4739	neural precursor cell expressed, developmentally down-regulated 9	10	GO:BP; GO:CC	7417.6	35.5	24.3	27	1.41	1.13e-07
PPFIA1	8500	PTPRF interacting protein alpha 1	10	GO:BP; GO:CC; KEGG; REAC	6284.9	34.4	20.6	27.2	1.43	1.13e-07
PXN	5829	paxillin	10	GO:BP; GO:CC; KEGG	6392.5	32.8	21.1	25.9	1.41	1.13e-07
RIMS2	9699	regulating synaptic membrane exocytosis 2	10	GO:BP; GO:CC; GO:MF	6294.9	33.3	20.5	26.4	1.41	1.13e-07
SPTA1	6708	spectrin alpha, erythrocytic 1	10	GO:BP; GO:CC; GO:MF; REAC	6359	33.4	20.8	26.6	1.41	1.13e-07
ARHGAP24	83478	Rho GTPase activating protein 24	10	GO:BP; GO:CC; GO:MF; REAC	6443.1	35.1	21.2	27.9	1.51	1.21e-07
DOCK1	1793	dedicator of cytokinesis 1	10	GO:BP; GO:MF; REAC	7432.2	36.2	24.4	27.6	1.37	1.21e-07
DOCK2	1794	dedicator of cytokinesis 2	10	GO:BP; GO:MF; REAC	7155	35.7	23.5	27.3	1.38	1.21e-07
DOCK4	9732	dedicator of cytokinesis 4	10	GO:BP; GO:CC; GO:MF; REAC	6769.9	35.4	22.3	27.8	1.36	1.21e-07
SIPA1L1	26037	signal induced proliferation associated 1 like 1	10	GO:BP; GO:CC; GO:MF; REAC	6528.6	35.4	21.5	27.8	1.47	1.21e-07
CHRNA3	1136	cholinergic receptor nicotinic alpha 3 subunit	10	GO:BP; GO:CC; KEGG; REAC	6750.3	32.8	22.2	25.6	1.34	2.98e-07
LRP4	4038	LDL receptor related protein 4	10	GO:BP; GO:CC	6312.3	32.7	20.8	25.9	1.38	2.98e-07
MTMR2	8898	myotubularin related protein 2	10	GO:BP; GO:CC; REAC	6884.4	35.2	22.7	27.4	1.38	2.98e-07
NECTIN1	5818	nectin cell adhesion molecule 1	10	GO:BP; GO:CC; KEGG	6585.1	35.2	21.7	27.7	1.33	2.98e-07
NEURL1	9148	neuralized E3 ubiquitin protein ligase 1	10	GO:BP; GO:CC; GO:MF; REAC	7301.4	35.6	24	27.2	1.38	2.98e-07
PDLIM5	10611	PDZ and LIM domain 5	10	GO:BP; GO:CC; GO:MF; REAC	6714.4	34.4	22.2	27.1	1.47	2.98e-07
PTPRO	5800	protein tyrosine phosphatase receptor type O	10	GO:BP; GO:CC	6670.1	33.4	21.9	26.1	1.38	2.98e-07
SLC4A8	9498	solute carrier family 4 member 8	10	GO:BP; GO:CC	6738.8	33.8	22.1	26.2	1.36	2.98e-07
CSK	1445	C-terminal Src kinase	10	GO:BP; GO:CC; KEGG; REAC	6983.3	38.5	22.9	30.1	1.49	3.66e-07
FGB	2244	fibrinogen beta chain	10	GO:BP; KEGG; REAC	7206.7	34	23.7	26.3	1.33	3.66e-07
FGG	2266	fibrinogen gamma chain	10	GO:BP; KEGG; REAC	7206.7	34	23.7	26.3	1.33	3.66e-07
FOXO3	2309	forkhead box O3	10	GO:BP; REAC	7390.4	35	24.3	26.5	1.30	3.66e-07
LRP5	4041	LDL receptor related protein 5	10	GO:BP; GO:CC; REAC	7811.9	34.9	25.7	26.1	1.30	3.66e-07
MAPKAP1	79109	MAPK associated protein 1	10	GO:BP; GO:MF; REAC	6514.5	35.9	21.5	28.2	1.59	3.66e-07
STAT5B	6777	signal transducer and activator of transcription 5B	10	GO:BP; REAC	7869.1	36.6	25.8	27.3	1.32	3.66e-07
TLN1	7094	talin 1	10	GO:BP; GO:CC; GO:MF; REAC	6539.5	33.8	21.5	26.8	1.43	3.66e-07
CUL3	8452	cullin 3	10	GO:BP; GO:MF; REAC	7474.9	36	24.5	27.4	1.38	7.84e-07
NCK1	4690	NCK adaptor protein 1	10	GO:BP; GO:CC; KEGG; REAC	7241.3	35.2	23.8	27.3	1.37	7.84e-07
NTN1	9423	netrin 1	10	GO:BP; GO:CC; KEGG	6840.9	35	22.5	27.2	1.41	2.37e-06
EPB41L5	57669	erythrocyte membrane protein band 4.1 like 5	10	GO:BP; GO:CC; REAC	7031.3	34.3	23.2	26.7	1.47	4.89e-06
GABBR1	2550	gamma-aminobutyric acid type B receptor subunit 1	10	GO:BP; GO:CC; KEGG; REAC	6693.8	38.3	21.9	30.6	1.56	4.89e-06
ITGB2	3689	integrin subunit beta 2	10	GO:BP; GO:CC; KEGG	6906	34.1	22.7	26.2	1.36	8.20e-06
PRKCQ	5588	protein kinase C theta	10	GO:BP; GO:MF; KEGG; REAC	6727.3	36.4	22.1	28.3	1.43	8.20e-06
ATP6V1A	523	ATPase H+ transporting V1 subunit A	10	GO:BP; GO:CC; KEGG	6421.2	33	21.1	26	1.39	1.17e-05
IL4	3565	interleukin 4	10	GO:BP; KEGG	7669.2	37.2	25.2	27.8	1.43	1.17e-05
JAK1	3716	Janus kinase 1	10	GO:BP; GO:CC; KEGG; REAC	6797.7	34.4	22.3	26.7	1.47	1.17e-05
FLII	2314	FLII actin remodeling protein	10	GO:BP; GO:CC; GO:MF	6539.8	34	21.5	27	1.39	1.62e-05
NLRP3	114548	NLR family pyrin domain containing 3	10	GO:BP; GO:MF	7709.3	37.2	25.3	27.8	1.33	1.62e-05
CACNA1D	776	calcium voltage-gated channel subunit alpha1 D	10	GO:BP; GO:CC; KEGG; REAC	5415.1	33.7	17.9	27.7	1.56	2.71e-05
OXT	5020	oxytocin/neurophysin I prepropeptide	10	GO:BP; KEGG	6579	32.8	21.5	25.7	1.41	2.71e-05
DTNBP1	84062	dystrobrevin binding protein 1	10	GO:BP; GO:CC; KEGG	7826.4	36.2	25.7	27.4	1.36	5.11e-05
RAB11A	8766	RAB11A, member RAS oncogene family	10	GO:BP; GO:CC; GO:MF	6652	34.6	21.9	27.1	1.41	1.20e-04
SMURF1	57154	SMAD specific E3 ubiquitin protein ligase 1	10	GO:BP; GO:MF; REAC	6210.7	31.4	20.3	24.9	1.33	1.20e-04

medRxiv preprint doi: https://doi.org/10.64898/2026.09.29.26364351; this version posted October 1, 2026. The copyright holder for this preprint
(which was not certified by peer review) is the author/funder, who has granted medRxiv a license to display the preprint in perpetuity.
This article is a US Government work. It is not subject to copyright under 17 USC 105 and is also made available
for use under a CC0 license.

Gene label	Entrez ID	Gene name	Number of pathways	Sources	Average rank	Average tags (%)	Average list (%)	Average signal (%)	Maximum normalized enrichment score	Minimum q-value
STAU2	27067	staufen double-stranded RNA binding protein 2	10	GO:BP; GO:CC	7428.4	35.5	24.4	27	1.33	1.96e-04
BMPR1A	657	bone morphogenetic protein receptor type 1A	10	GO:BP; GO:CC; GO:MF; REAC	5418.5	31	17.8	25.4	1.65	2.04e-04
BRD4	23476	bromodomain containing 4	10	GO:BP; GO:CC; GO:MF	7780.5	37.5	25.5	27.8	1.30	2.04e-04
TTN	7273	titin	10	GO:BP; GO:CC; GO:MF	7256.8	38.3	23.9	29.3	1.56	2.04e-04
NRXN2	9379	neurexin 2	10	GO:BP; GO:CC; REAC	6049.8	32.7	19.8	26.2	1.47	2.98e-04
EDN1	1906	endothelin 1	10	GO:BP; GO:CC	8719.9	38.2	28.7	27.6	1.26	4.37e-04
IL1B	3553	interleukin 1 beta	10	GO:BP	8713.5	38.3	28.6	27.7	1.24	2.48e-03
IL23R	149233	interleukin 23 receptor	10	GO:BP; GO:CC	7947.9	37.2	26.2	27.6	1.30	2.48e-03
TNFSF4	7292	TNF superfamily member 4	10	GO:BP	7685.1	38.4	25.3	28.8	1.42	2.48e-03
VPS13A	23230	vacuolar protein sorting 13 homolog A	10	GO:BP; GO:CC; KEGG	6396.2	31.4	20.9	24.8	1.41	2.97e-03

Only genes that appeared in the leading-edge subset of at least 10 collapsed representative pathways are listed.

medRxiv preprint doi: https://doi.org/10.64898/2026.09.29.26364351; this version posted October 1, 2026. The copyright holder for this preprint (which was not certified by peer review) is the author/funder, who has granted medRxiv a license to display the preprint in perpetuity.
This article is a US Government work. It is not subject to copyright under 17 USC 105 and is also made available for use under a CC0 license.

Supplemental Table 7. Nominally Significant Biomarker-Respiratory Outcome Associations

Analysis Timing	Biomarker	Biomarker Type	Outcome	Effect scale	Effect	Beta / OR	SE (Beta only)	P	95% CI
Concurrent (1996 survey)	cg00916310	DMPs	Dyspnea	1-SD higher methylation beta	OR	1.771		1.3500E-02	[1.125, 2.788]
Concurrent (1996 survey)	cg01778030	DMPs	Dyspnea	1-SD higher methylation beta	OR	1.882		1.8253E-02	[1.113, 3.182]
Concurrent (1996 survey)	cg01778030	DMPs	FVC % predicted	1-SD higher methylation beta	Beta	2.006	0.877	2.2590E-02	[0.287, 3.725]
Concurrent (1996 survey)	cg01778030	DMPs	FVC (mL)	1-SD higher methylation beta	Beta	63.956	32.372	4.8768E-02	[0.507, 127.404]
Concurrent (1996 survey)	cg01833052	DMPs	Dyspnea	1-SD higher methylation beta	OR	1.744		4.0467E-02	[1.024, 2.969]
Concurrent (1996 survey)	cg08009902	DMPs	FEV-1/FVC <0.70	1-SD higher methylation beta	OR	0.728		4.2695E-02	[0.536, 0.990]
Concurrent (1996 survey)	cg08129102	DMPs	FEV-1/FVC ratio	1-SD higher methylation beta	Beta	-0.008	0.004	3.6477E-02	[-0.015, -0.001]
Concurrent (1996 survey)	cg11248361	DMPs	FEV-1/FVC ratio	1-SD higher methylation beta	Beta	0.009	0.004	2.7731E-02	[0.001, 0.017]
Concurrent (1996 survey)	cg11795976	DMPs	Dyspnea	1-SD higher methylation beta	OR	1.803		3.0975E-02	[1.055, 3.080]
Concurrent (1996 survey)	cg16290931	DMPs	Dyspnea	1-SD higher methylation beta	OR	1.559		4.2444E-02	[1.015, 2.395]
Concurrent (1996 survey)	cg16371865	DMPs	Chronic cough	1-SD higher methylation beta	OR	2.151		3.8329E-02	[1.042, 4.442]
Concurrent (1996 survey)	cg19055984	DMPs	FEV-1/FVC < GLI LLN	1-SD higher methylation beta	OR	1.457		2.3074E-02	[1.053, 2.015]
Concurrent (1996 survey)	cg19777190	DMPs	Chronic bronchitis	1-SD higher methylation beta	OR	0.414		1.4248E-02	[0.204, 0.838]
Concurrent (1996 survey)	cg19777190	DMPs	Chronic cough	1-SD higher methylation beta	OR	0.454		4.3479E-02	[0.211, 0.977]
Concurrent (1996 survey)	cg22740441	DMPs	Dyspnea	1-SD higher methylation beta	OR	1.724		4.2204E-02	[1.019, 2.915]
Concurrent (1996 survey)	cg26033526	DMPs	Dyspnea	1-SD higher methylation beta	OR	1.984		7.4708E-03	[1.201, 3.277]
Concurrent (1996 survey)	cg26491213	DMPs	FEV-1/FVC <0.70	1-SD higher methylation beta	OR	0.660		5.3918E-03	[0.493, 0.884]
Concurrent (1996 survey)	cg26491213	DMPs	FEV-1/FVC < GLI LLN	1-SD higher methylation beta	OR	0.690		1.9398E-02	[0.506, 0.942]
Concurrent (1996 survey)	cg26491213	DMPs	FEV-1 % predicted	1-SD higher methylation beta	Beta	1.426	0.638	2.5869E-02	[0.176, 2.677]
Concurrent (1996 survey)	cg26947969	DMPs	Dyspnea	1-SD higher methylation beta	OR	1.449		4.3993E-02	[1.010, 2.078]
Concurrent (1996 survey)	IRS$_{\text{(Hillary-BayesPR)}}$	IRS	FEV-1/FVC <0.70	1-SD higher score	OR	1.581		1.6193E-03	[1.189, 2.103]
Concurrent (1996 survey)	IRS$_{\text{(Hillary-BayesPR)}}$	IRS	FEV-1/FVC < GLI LLN	1-SD higher score	OR	1.573		3.1269E-03	[1.165, 2.125]
Concurrent (1996 survey)	IRS$_{\text{(Hillary-BayesPR)}}$	IRS	FEV-1 (mL)	1-SD higher score	Beta	-42.722	18.167	1.9099E-02	[-78.329, -7.115]
Concurrent (1996 survey)	IRS$_{\text{(Hillary-BayesPR)}}$	IRS	FEV-1 % predicted	1-SD higher score	Beta	-1.410	0.623	2.4116E-02	[-2.631, -0.188]
Concurrent (1996 survey)	IRS$_{\text{(Hillary-elastic-net)}}$	IRS	FEV-1/FVC < GLI LLN	1-SD higher score	OR	1.522		7.5943E-03	[1.118, 2.072]
Concurrent (1996 survey)	IRS$_{\text{(Hillary-elastic-net)}}$	IRS	FEV-1/FVC <0.70	1-SD higher score	OR	1.466		9.4640E-03	[1.098, 1.956]
Concurrent (1996 survey)	IRS$_{\text{(Hillary-elastic-net)}}$	IRS	FEV-1 (mL)	1-SD higher score	Beta	-40.087	18.647	3.2077E-02	[-76.634, -3.539]
Concurrent (1996 survey)	IRS$_{\text{(Hillary-elastic-net)}}$	IRS	FEV-1 % predicted	1-SD higher score	Beta	-1.295	0.639	4.3369E-02	[-2.548, -0.042]
Concurrent (1996 survey)	IRS$_{\text{(Stevenson)}}$	IRS	FVC % predicted	1-SD higher score	Beta	2.048	0.770	8.0628E-03	[0.539, 3.557]
Concurrent (1996 survey)	IRS$_{\text{(Stevenson)}}$	IRS	FEV-1/FVC < GLI LLN	1-SD higher score	OR	1.614		3.4772E-02	[1.035, 2.517]
Prospective	cg01778030	DMPs	Chronic cough 2001	1-SD higher methylation beta	OR	1.832		4.5705E-02	[1.012, 3.319]
Prospective	cg04522575	DMPs	Annual FVC change 1996–2006	1-SD higher methylation beta	Beta	-5.137	1.926	7.9654E-03	[-8.913, -1.362]
Prospective	cg04522575	DMPs	Annual FVC change 1996–2011	1-SD higher methylation beta	Beta	-3.225	1.277	1.1962E-02	[-5.728, -0.723]
Prospective	cg05019530	DMPs	Annual FVC change 1996–2011	1-SD higher methylation beta	Beta	-3.461	1.380	1.2541E-02	[-6.165, -0.757]
Prospective	cg05408620	DMPs	Chronic bronchitis 2001	1-SD higher methylation beta	OR	1.978		2.9454E-02	[1.070, 3.657]
Prospective	cg05801388	DMPs	Annual FVC change 1996–2016	1-SD higher methylation beta	Beta	1.841	0.896	4.0818E-02	[0.084, 3.597]
Prospective	cg05801388	DMPs	FEV-1/FVC ratio 2001	1-SD higher methylation beta	Beta	0.007	0.003	4.5907E-02	[0.000, 0.013]
Prospective	cg08009902	DMPs	FEV-1/FVC ratio 2016	1-SD higher methylation beta	Beta	0.010	0.004	8.3516E-03	[0.003, 0.018]
Prospective	cg08009902	DMPs	FEV-1/FVC < GLI LLN 2001	1-SD higher methylation beta	OR	0.535		9.0123E-03	[0.335, 0.856]
Prospective	cg08009902	DMPs	FEV-1/FVC < GLI LLN 2006	1-SD higher methylation beta	OR	0.584		1.8344E-02	[0.374, 0.913]
Prospective	cg08009902	DMPs	FEV-1/FVC < GLI LLN 2011	1-SD higher methylation beta	OR	0.530		2.7134E-02	[0.302, 0.931]
Prospective	cg08009902	DMPs	FEV-1/FVC <0.70 2011	1-SD higher methylation beta	OR	0.658		3.5551E-02	[0.445, 0.972]
Prospective	cg08009902	DMPs	FEV-1/FVC ratio 2006	1-SD higher methylation beta	Beta	0.008	0.004	3.7471E-02	[0.001, 0.016]
Prospective	cg08009902	DMPs	FEV-1/FVC ratio 2011	1-SD higher methylation beta	Beta	0.008	0.004	4.3771E-02	[0.000, 0.015]
Prospective	cg08009902	DMPs	FEV-1/FVC <0.70 2001	1-SD higher methylation beta	OR	0.686		4.9336E-02	[0.471, 0.999]
Prospective	cg08129102	DMPs	FEV-1/FVC ratio 2001	1-SD higher methylation beta	Beta	-0.007	0.003	3.1917E-02	[-0.014, -0.001]
Prospective	cg10264003	DMPs	Annual FVC change 1996–2006	1-SD higher methylation beta	Beta	-5.494	2.352	1.9970E-02	[-10.103, -0.885]
Prospective	cg10793507	DMPs	FVC (mL) 2011	1-SD higher methylation beta	Beta	-88.826	26.702	9.6192E-04	[-141.161, -36.490]
Prospective	cg10793507	DMPs	FVC % predicted 2011	1-SD higher methylation beta	Beta	-2.623	0.799	1.1222E-03	[-4.190, -1.057]
Prospective	cg10793507	DMPs	Annual FVC change 1996–2011	1-SD higher methylation beta	Beta	-3.531	1.135	2.0130E-03	[-5.757, -1.306]
Prospective	cg10793507	DMPs	FEV-1/FVC <0.70 2011	1-SD higher methylation beta	OR	0.587		5.9906E-03	[0.402, 0.858]
Prospective	cg10793507	DMPs	FVC % predicted 2006	1-SD higher methylation beta	Beta	-1.900	0.702	7.0604E-03	[-3.276, -0.524]
Prospective	cg10793507	DMPs	FVC (mL) 2006	1-SD higher methylation beta	Beta	-63.520	24.499	9.8539E-03	[-111.538, -15.501]

medRxiv preprint doi: https://doi.org/10.64898/2026.09.29.26364351; this version posted October 1, 2026. The copyright holder for this preprint
(which was not certified by peer review) is the author/funder, who has granted medRxiv a license to display the preprint in perpetuity.
This article is a US Government work. It is not subject to copyright under 17 USC 105 and is also made available
for use under a CC0 license.

Analysis Timing	Biomarker	Biomarker Type	Outcome	Effect scale	Effect	Beta / OR	SE (Beta only)	P	95% CI
Prospective	cg10793507	DMPs	FVC (mL) 2016	1-SD higher methylation beta	Beta	-67.709	30.346	2.6365E-02	[-127.188, -8.231]
Prospective	cg10793507	DMPs	FEV-1/FVC < GLI LLN 2011	1-SD higher methylation beta	OR	0.559		2.9161E-02	[0.332, 0.943]
Prospective	cg10793507	DMPs	FVC % predicted 2016	1-SD higher methylation beta	Beta	-2.081	1.001	3.8446E-02	[-4.044, -0.119]
Prospective	cg10793507	DMPs	FEV-1/FVC ratio 2011	1-SD higher methylation beta	Beta	0.008	0.004	3.8683E-02	[0.000, 0.016]
Prospective	cg11248361	DMPs	FEV-1/FVC <0.70 2011	1-SD higher methylation beta	OR	0.619		2.4163E-02	[0.408, 0.939]
Prospective	cg11248361	DMPs	FEV-1/FVC ratio 2001	1-SD higher methylation beta	Beta	0.008	0.004	3.3971E-02	[0.001, 0.015]
Prospective	cg11795976	DMPs	FEV-1 % predicted 2016	1-SD higher methylation beta	Beta	3.906	1.537	1.1528E-02	[0.893, 6.920]
Prospective	cg11795976	DMPs	Byssinosis 2001	1-SD higher methylation beta	OR	3.249		1.5412E-02	[1.252, 8.427]
Prospective	cg11795976	DMPs	FVC % predicted 2016	1-SD higher methylation beta	Beta	3.287	1.459	2.4883E-02	[0.429, 6.146]
Prospective	cg12318285	DMPs	FEV-1/FVC < GLI LLN 2006	1-SD higher methylation beta	OR	1.791		3.5171E-02	[1.041, 3.082]
Prospective	cg12318285	DMPs	FVC (mL) 2001	1-SD higher methylation beta	Beta	-50.163	24.525	4.1331E-02	[-98.232, -2.095]
Prospective	cg15499393	DMPs	FEV-1/FVC ratio 2016	1-SD higher methylation beta	Beta	0.012	0.006	4.7981E-02	[0.000, 0.023]
Prospective	cg16290931	DMPs	Annual FEV-1 change 1996–2001	1-SD higher methylation beta	Beta	4.464	1.902	1.9363E-02	[0.736, 8.193]
Prospective	cg16371865	DMPs	FEV-1/FVC ratio 2016	1-SD higher methylation beta	Beta	0.009	0.004	1.8192E-02	[0.002, 0.016]
Prospective	cg16371865	DMPs	FEV-1 (mL) 2016	1-SD higher methylation beta	Beta	52.024	22.740	2.2807E-02	[7.454, 96.594]
Prospective	cg16371865	DMPs	FEV-1 % predicted 2016	1-SD higher methylation beta	Beta	2.019	0.950	3.4329E-02	[0.157, 3.881]
Prospective	cg19055984	DMPs	Chronic bronchitis 2001	1-SD higher methylation beta	OR	0.643		2.5126E-02	[0.436, 0.946]
Prospective	cg19055984	DMPs	FEV-1/FVC ratio 2006	1-SD higher methylation beta	Beta	-0.009	0.004	2.6069E-02	[-0.016, -0.001]
Prospective	cg19055984	DMPs	FEV-1 % predicted 2006	1-SD higher methylation beta	Beta	-1.637	0.746	2.8873E-02	[-3.100, -0.174]
Prospective	cg19055984	DMPs	FEV-1/FVC < GLI LLN 2006	1-SD higher methylation beta	OR	1.580		4.5806E-02	[1.009, 2.475]
Prospective	cg19925755	DMPs	Annual FVC change 1996–2006	1-SD higher methylation beta	Beta	-3.348	1.509	2.7056E-02	[-6.305, -0.391]
Prospective	cg22740441	DMPs	FEV-1 % predicted 2016	1-SD higher methylation beta	Beta	3.033	1.528	4.8060E-02	[0.037, 6.029]
Prospective	cg22766862	DMPs	Annual FVC change 1996–2011	1-SD higher methylation beta	Beta	-2.505	1.157	3.1029E-02	[-4.774, -0.237]
Prospective	cg25022932	DMPs	FEV-1/FVC ratio 2001	1-SD higher methylation beta	Beta	0.007	0.003	3.1120E-02	[0.001, 0.014]
Prospective	cg26277709	DMPs	FEV-1/FVC ratio 2001	1-SD higher methylation beta	Beta	0.007	0.003	2.7240E-02	[0.001, 0.014]
Prospective	cg26277709	DMPs	FEV-1/FVC <0.70 2011	1-SD higher methylation beta	OR	0.669		3.5456E-02	[0.460, 0.973]
Prospective	cg26491213	DMPs	Chronic cough 2001	1-SD higher methylation beta	OR	0.607		1.3478E-02	[0.408, 0.902]
Prospective	cg26491213	DMPs	FEV-1/FVC <0.70 2006	1-SD higher methylation beta	OR	0.677		2.6005E-02	[0.480, 0.954]
Prospective	cg26491213	DMPs	FEV-1/FVC < GLI LLN 2001	1-SD higher methylation beta	OR	0.625		3.8361E-02	[0.400, 0.975]
Prospective	cg26491213	DMPs	Chronic bronchitis 2001	1-SD higher methylation beta	OR	0.667		4.0354E-02	[0.453, 0.982]
Prospective	cg26491213	DMPs	FEV-1 % predicted 2006	1-SD higher methylation beta	Beta	1.468	0.721	4.2494E-02	[0.054, 2.882]
Prospective	cg26491213	DMPs	FVC % predicted 2006	1-SD higher methylation beta	Beta	1.278	0.648	4.9237E-02	[0.008, 2.548]
Prospective	IRS$_{\text{(Hillary-BayesPR)}}$	IRS	FEV-1 (mL) 2001	1-SD higher score	Beta	-56.582	16.994	9.3377E-04	[-89.890, -23.274]
Prospective	IRS$_{\text{(Hillary-BayesPR)}}$	IRS	FEV-1 % predicted 2001	1-SD higher score	Beta	-1.952	0.621	1.7591E-03	[-3.169, -0.735]
Prospective	IRS$_{\text{(Hillary-BayesPR)}}$	IRS	FVC (mL) 2016	1-SD higher score	Beta	-76.760	27.934	6.3421E-03	[-131.511, -22.008]
Prospective	IRS$_{\text{(Hillary-BayesPR)}}$	IRS	Dyspnea 2001	1-SD higher score	OR	1.440		6.4571E-03	[1.108, 1.872]
Prospective	IRS$_{\text{(Hillary-BayesPR)}}$	IRS	FVC (mL) 2001	1-SD higher score	Beta	-51.857	19.388	7.7215E-03	[-89.857, -13.857]
Prospective	IRS$_{\text{(Hillary-BayesPR)}}$	IRS	FEV-1 % predicted 2011	1-SD higher score	Beta	-2.034	0.796	1.0958E-02	[-3.593, -0.474]
Prospective	IRS$_{\text{(Hillary-BayesPR)}}$	IRS	FEV-1 (mL) 2011	1-SD higher score	Beta	-51.360	20.451	1.2426E-02	[-91.444, -11.277]
Prospective	IRS$_{\text{(Hillary-BayesPR)}}$	IRS	FVC (mL) 2011	1-SD higher score	Beta	-60.724	24.307	1.2890E-02	[-108.367, -13.082]
Prospective	IRS$_{\text{(Hillary-BayesPR)}}$	IRS	FVC % predicted 2001	1-SD higher score	Beta	-1.382	0.555	1.3060E-02	[-2.470, -0.295]
Prospective	IRS$_{\text{(Hillary-BayesPR)}}$	IRS	FEV-1 (mL) 2016	1-SD higher score	Beta	-56.910	23.216	1.4773E-02	[-102.415, -11.406]
Prospective	IRS$_{\text{(Hillary-BayesPR)}}$	IRS	FEV-1/FVC < GLI LLN 2001	1-SD higher score	OR	1.654		1.4804E-02	[1.104, 2.480]
Prospective	IRS$_{\text{(Hillary-BayesPR)}}$	IRS	FEV-1 (mL) 2006	1-SD higher score	Beta	-47.408	19.442	1.5166E-02	[-85.515, -9.301]
Prospective	IRS$_{\text{(Hillary-BayesPR)}}$	IRS	FVC % predicted 2016	1-SD higher score	Beta	-2.251	0.926	1.5647E-02	[-4.066, -0.435]
Prospective	IRS$_{\text{(Hillary-BayesPR)}}$	IRS	FVC % predicted 2011	1-SD higher score	Beta	-1.742	0.725	1.6715E-02	[-3.163, -0.321]
Prospective	IRS$_{\text{(Hillary-BayesPR)}}$	IRS	FEV-1 % predicted 2006	1-SD higher score	Beta	-1.659	0.715	2.0870E-02	[-3.061, -0.257]
Prospective	IRS$_{\text{(Hillary-BayesPR)}}$	IRS	FEV-1/FVC < GLI LLN 2006	1-SD higher score	OR	1.656		2.1448E-02	[1.077, 2.544]
Prospective	IRS$_{\text{(Hillary-BayesPR)}}$	IRS	FEV-1/FVC <0.70 2016	1-SD higher score	OR	1.713		2.2921E-02	[1.077, 2.725]
Prospective	IRS$_{\text{(Hillary-BayesPR)}}$	IRS	FEV-1 % predicted 2016	1-SD higher score	Beta	-2.224	0.980	2.3871E-02	[-4.144, -0.304]
Prospective	IRS$_{\text{(Hillary-BayesPR)}}$	IRS	Chronic bronchitis 2001	1-SD higher score	OR	1.486		2.6844E-02	[1.046, 2.109]
Prospective	IRS$_{\text{(Hillary-BayesPR)}}$	IRS	FEV-1/FVC <0.70 2001	1-SD higher score	OR	1.451		2.9322E-02	[1.038, 2.028]
Prospective	IRS$_{\text{(Hillary-elastic-net)}}$	IRS	FVC (mL) 2016	1-SD higher score	Beta	-96.097	28.682	9.0444E-04	[-152.314, -39.880]
Prospective	IRS$_{\text{(Hillary-elastic-net)}}$	IRS	FEV-1 (mL) 2001	1-SD higher score	Beta	-54.698	17.431	1.8008E-03	[-88.863, -20.534]
Prospective	IRS$_{\text{(Hillary-elastic-net)}}$	IRS	FEV-1 (mL) 2016	1-SD higher score	Beta	-74.656	23.833	1.8948E-03	[-121.368, -27.944]

medRxiv preprint doi: https://doi.org/10.64898/2026.09.29.26364351; this version posted October 1, 2026. The copyright holder for this preprint (which was not certified by peer review) is the author/funder, who has granted medRxiv a license to display the preprint in perpetuity.
This article is a US Government work. It is not subject to copyright under 17 USC 105 and is also made available for use under a CC0 license.

Analysis Timing	Biomarker	Biomarker Type	Outcome	Effect scale	Effect	Beta / OR	SE (Beta only)	P	95% CI
Prospective	IRS$_{\text{(Hillary-elastic-net)}}$	IRS	FVC % predicted 2016	1-SD higher score	Beta	-2.873	0.951	2.7320E-03	[-4.738, -1.008]
Prospective	IRS$_{\text{(Hillary-elastic-net)}}$	IRS	FEV-1 % predicted 2001	1-SD higher score	Beta	-1.904	0.636	2.9011E-03	[-3.151, -0.657]
Prospective	IRS$_{\text{(Hillary-elastic-net)}}$	IRS	FEV-1 % predicted 2016	1-SD higher score	Beta	-2.904	1.006	4.1613E-03	[-4.877, -0.932]
Prospective	IRS$_{\text{(Hillary-elastic-net)}}$	IRS	FEV-1 (mL) 2011	1-SD higher score	Beta	-56.211	20.786	7.1440E-03	[-96.952, -15.470]
Prospective	IRS$_{\text{(Hillary-elastic-net)}}$	IRS	FEV-1 % predicted 2011	1-SD higher score	Beta	-2.169	0.805	7.3738E-03	[-3.748, -0.591]
Prospective	IRS$_{\text{(Hillary-elastic-net)}}$	IRS	FVC (mL) 2011	1-SD higher score	Beta	-63.894	24.724	1.0117E-02	[-112.352, -15.436]
Prospective	IRS$_{\text{(Hillary-elastic-net)}}$	IRS	Dyspnea 2001	1-SD higher score	OR	1.415		1.0641E-02	[1.084, 1.848]
Prospective	IRS$_{\text{(Hillary-elastic-net)}}$	IRS	FEV-1 (mL) 2006	1-SD higher score	Beta	-49.754	19.962	1.3069E-02	[-88.879, -10.629]
Prospective	IRS$_{\text{(Hillary-elastic-net)}}$	IRS	FVC % predicted 2011	1-SD higher score	Beta	-1.820	0.734	1.3563E-02	[-3.259, -0.382]
Prospective	IRS$_{\text{(Hillary-elastic-net)}}$	IRS	FVC (mL) 2001	1-SD higher score	Beta	-49.144	19.876	1.3743E-02	[-88.101, -10.188]
Prospective	IRS$_{\text{(Hillary-elastic-net)}}$	IRS	FEV-1/FVC <0.70 2001	1-SD higher score	OR	1.527		1.7140E-02	[1.078, 2.163]
Prospective	IRS$_{\text{(Hillary-elastic-net)}}$	IRS	FVC % predicted 2001	1-SD higher score	Beta	-1.350	0.568	1.7916E-02	[-2.464, -0.236]
Prospective	IRS$_{\text{(Hillary-elastic-net)}}$	IRS	FEV-1 % predicted 2006	1-SD higher score	Beta	-1.709	0.732	2.0124E-02	[-3.144, -0.273]
Prospective	IRS$_{\text{(Hillary-elastic-net)}}$	IRS	FEV-1/FVC <0.70 2016	1-SD higher score	OR	1.748		2.0155E-02	[1.091, 2.800]
Prospective	IRS$_{\text{(Stevenson)}}$	IRS	FEV-1/FVC <0.70 2016	1-SD higher score	OR	2.595		3.9690E-03	[1.356, 4.965]
Prospective	IRS$_{\text{(Stevenson)}}$	IRS	FEV-1/FVC < GLI LLN 2011	1-SD higher score	OR	2.689		1.0943E-02	[1.255, 5.759]
Prospective	IRS$_{\text{(Stevenson)}}$	IRS	FEV-1/FVC <0.70 2011	1-SD higher score	OR	1.983		1.2879E-02	[1.156, 3.402]
Prospective	IRS$_{\text{(Stevenson)}}$	IRS	FEV-1/FVC ratio 2011	1-SD higher score	Beta	-0.013	0.005	1.6166E-02	[-0.023, -0.002]
Prospective	IRS$_{\text{(Stevenson)}}$	IRS	FEV-1/FVC < GLI LLN 2016	1-SD higher score	OR	2.451		3.4900E-02	[1.066, 5.639]
Prospective	IRS$_{\text{(Stevenson)}}$	IRS	Annual FVC change 1996–2001	1-SD higher score	Beta	-7.901	3.777	3.6970E-02	[-15.303, -0.499]
Prospective	IRS$_{\text{(Ligthart)}}$	IRS	FEV-1 (mL) 2016	1-SD higher score	Beta	-68.726	25.781	8.0753E-03	[-119.258, -18.195]
Prospective	IRS$_{\text{(Ligthart)}}$	IRS	FEV-1 % predicted 2016	1-SD higher score	Beta	-2.745	1.074	1.1026E-02	[-4.849, -0.641]
Prospective	IRS$_{\text{(Ligthart)}}$	IRS	Annual FVC change 1996–2016	1-SD higher score	Beta	-2.402	1.003	1.7257E-02	[-4.368, -0.436]
Prospective	IRS$_{\text{(Ligthart)}}$	IRS	FVC (mL) 2016	1-SD higher score	Beta	-73.203	31.171	1.9467E-02	[-134.299, -12.108]
Prospective	IRS$_{\text{(Ligthart)}}$	IRS	FVC % predicted 2016	1-SD higher score	Beta	-2.328	1.018	2.2911E-02	[-4.324, -0.332]
Prospective	IRS$_{\text{(Ligthart)}}$	IRS	Annual FVC change 1996–2001	1-SD higher score	Beta	-6.429	2.851	2.4578E-02	[-12.016, -0.842]
Prospective	IRS$_{\text{(Ligthart)}}$	IRS	FVC % predicted 2001	1-SD higher score	Beta	-1.389	0.625	2.6579E-02	[-2.614, -0.165]
Prospective	IRS$_{\text{(Ligthart)}}$	IRS	FEV-1 % predicted 2001	1-SD higher score	Beta	-1.491	0.706	3.5074E-02	[-2.874, -0.108]
Prospective	IRS$_{\text{(Ligthart)}}$	IRS	Annual FEV-1 change 1996–2016	1-SD higher score	Beta	-1.473	0.725	4.3112E-02	[-2.895, -0.052]
Prospective	IRS$_{\text{(Ligthart)}}$	IRS	FEV-1/FVC <0.70 2016	1-SD higher score	OR	1.571		4.5960E-02	[1.008, 2.449]
Prospective	IRS$_{\text{(Wielscher)}}$	IRS	FEV-1 % predicted 2016	1-SD higher score	Beta	-4.190	1.372	2.4531E-03	[-6.880, -1.500]
Prospective	IRS$_{\text{(Wielscher)}}$	IRS	FEV-1/FVC <0.70 2016	1-SD higher score	OR	2.156		7.7800E-03	[1.224, 3.797]
Prospective	IRS$_{\text{(Wielscher)}}$	IRS	FEV-1 (mL) 2016	1-SD higher score	Beta	-87.994	33.158	8.3611E-03	[-152.984, -23.004]
Prospective	IRS$_{\text{(Wielscher)}}$	IRS	FVC % predicted 2016	1-SD higher score	Beta	-3.428	1.304	8.9714E-03	[-5.984, -0.873]
Prospective	IRS$_{\text{(Wielscher)}}$	IRS	FEV-1 % predicted 2011	1-SD higher score	Beta	-2.844	1.142	1.3146E-02	[-5.082, -0.606]
Prospective	IRS$_{\text{(Wielscher)}}$	IRS	FEV-1 % predicted 2001	1-SD higher score	Beta	-2.193	0.924	1.7987E-02	[-4.004, -0.382]
Prospective	IRS$_{\text{(Wielscher)}}$	IRS	FVC (mL) 2016	1-SD higher score	Beta	-94.495	40.084	1.9008E-02	[-173.059, -15.932]
Prospective	IRS$_{\text{(Wielscher)}}$	IRS	FVC % predicted 2001	1-SD higher score	Beta	-1.831	0.822	2.6433E-02	[-3.442, -0.219]
Prospective	IRS$_{\text{(Wielscher)}}$	IRS	FEV-1 (mL) 2011	1-SD higher score	Beta	-59.885	29.926	4.6070E-02	[-118.540, -1.230]

Separate multivariable linear regression models for continuous outcomes and logistic regression models for binary outcomes were adjusted for log cumulative endotoxin, baseline age, sex, height, years since cessation of endotoxin exposure, smoking pack-years, body mass index, EpiDISH-estimated immune-cell fractions, and Sample Plate. Age, sex, and height were omitted from models of GLI percent-predicted FEV-1 and FVC because these characteristics are incorporated in the reference equations. DNAm biomarkers and covariates were measured in 1996; concurrent outcomes were measured in 1996 and prospective outcomes in 2001, 2006, 2011, or 2016. DMP associations are expressed per 1-SD higher methylation beta value and IRS associations per 1-SD higher score. Continuous-outcome effects are beta coefficients; binary-outcome effects are odds ratios. The table includes nominal associations at $P<0.05$ with 95% model-based confidence intervals. Abbreviations: BMI=body mass index; DMP=differentially methylated position; DNAm=DNA methylation; FEV-1=forced expiratory volume in 1 second; FVC=forced vital capacity; GLI=Global Lung Function Initiative; IRS=inflammation risk score; SD=standard deviation.

medRxiv preprint doi: https://doi.org/10.64898/2026.09.29.26364351; this version posted October 1, 2026. The copyright holder for this preprint (which was not certified by peer review) is the author/funder, who has granted medRxiv a license to display the preprint in perpetuity.
This article is a US Government work. It is not subject to copyright under 17 USC 105 and is also made available for use under a CC0 license.

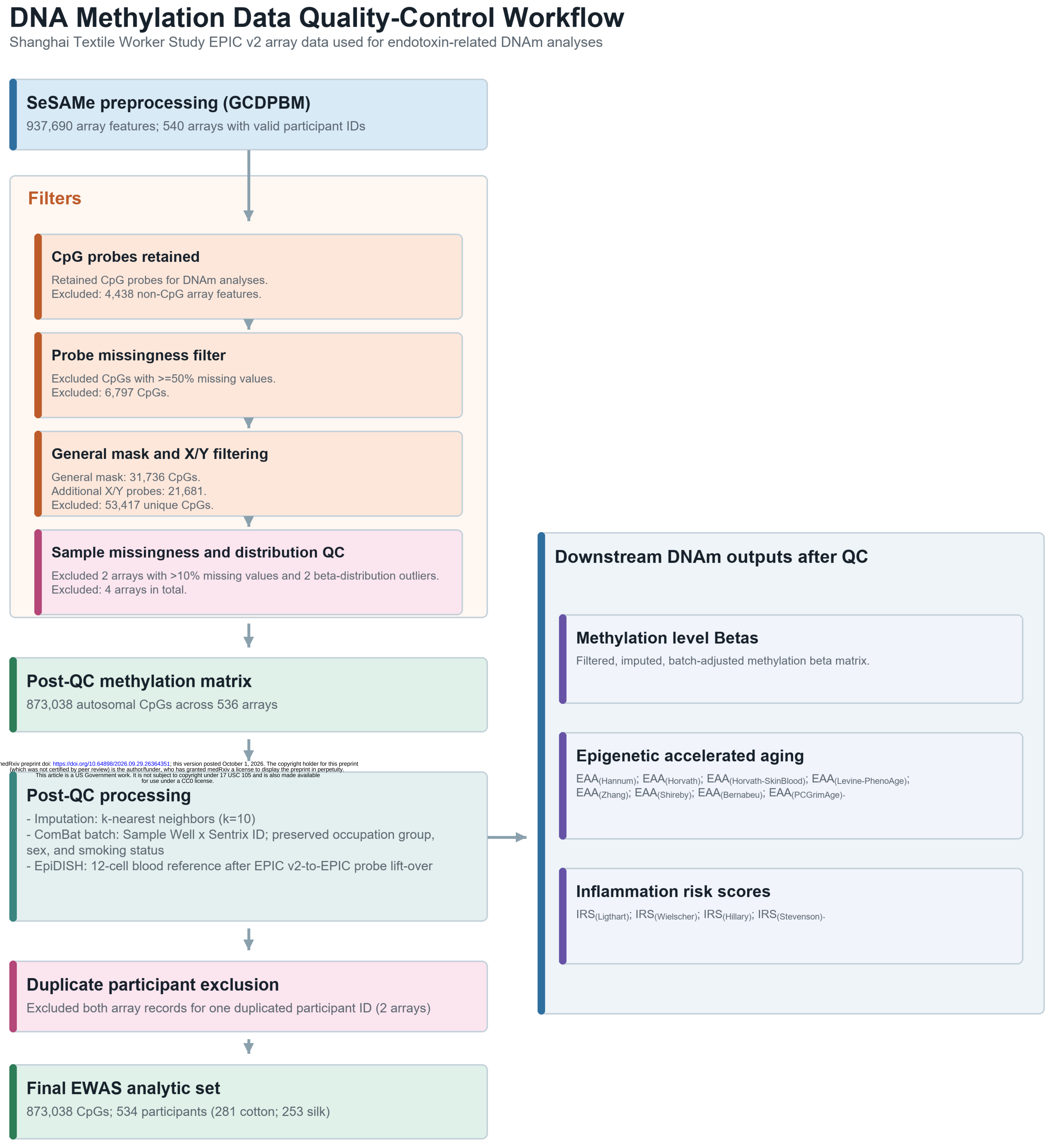

Supplemental Figure 1. DNA methylation data quality-control workflow.

Raw IDATs were processed in SeSAMe using the GCDPBM preprocessing sequence. The STWS EPIC v2 matrix included 937,690 array features across 540 arrays linked to valid participant IDs. Probe filtering removed 4,438 non-CpG features and 6,797 CpGs with >=50% missing values. The EPIC v2 general mask removed 31,736 CpGs, followed by 21,681 additional X/Y probes (53,417 unique exclusions), leaving 873,038 autosomal CpGs. Sample QC removed two arrays with >10% missing values and two beta-distribution outliers, leaving 536 post-QC arrays. Both records for one duplicated participant ID were excluded before the EWAS, leaving 534 participants (281 cotton; 253 silk). Missing values were imputed by k-nearest neighbors (k=10). ComBat used Sample Well x Sentrix ID as the batch variable while preserving occupation group, sex, and smoking status. EpiDISH used a 12-cell blood reference after EPIC v2-to-EPIC probe lift-over. Abbreviations: ComBat=batch-effect correction using empirical Bayes; DNAm=DNA methylation; EAA=epigenetic age acceleration; EpiDISH=Epigenetic Dissection of Intra-Sample Heterogeneity; IRS=inflammation risk score; QC=quality control; STWS=Shanghai Textile Worker Study.

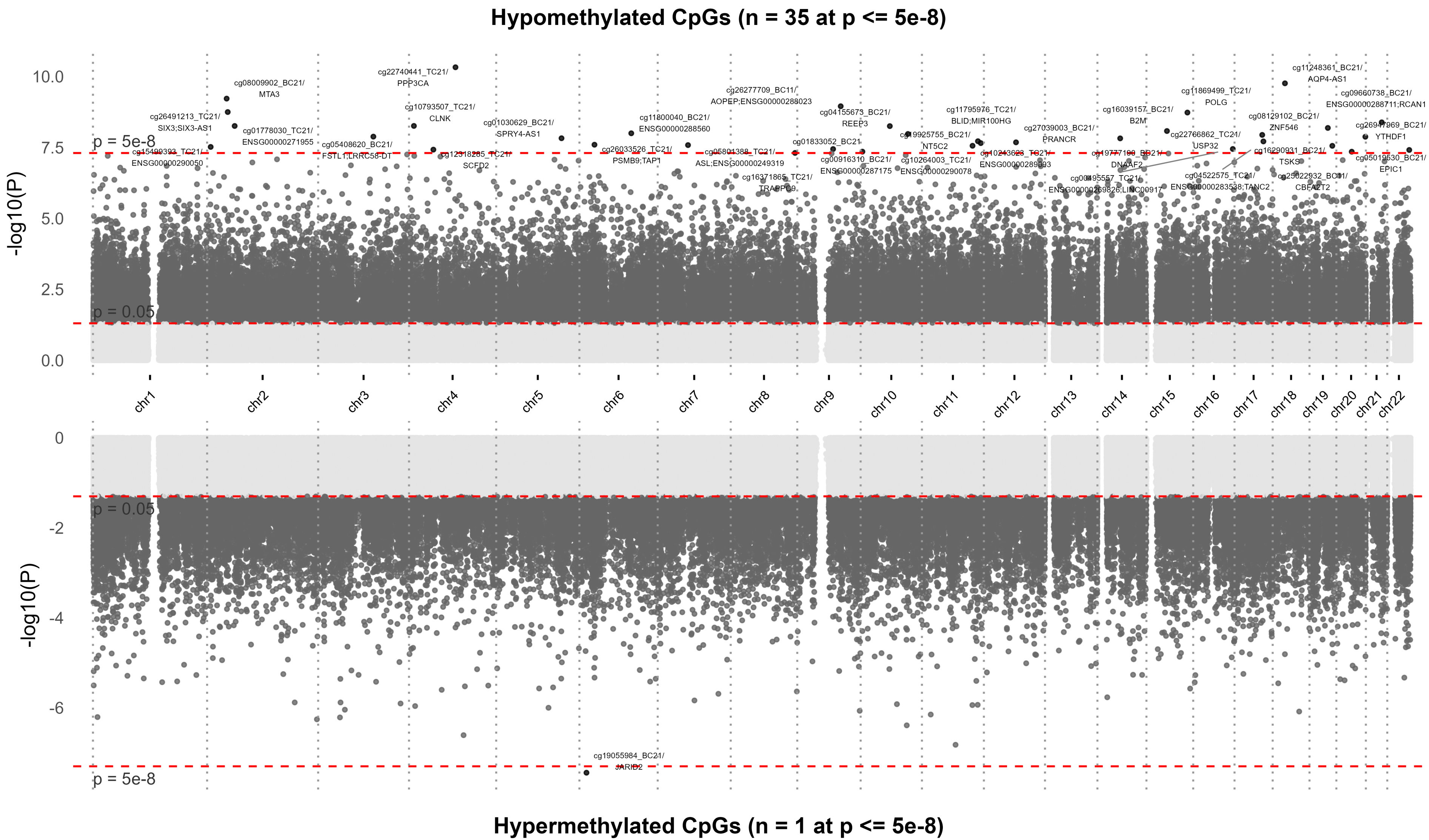

Supplemental Figure 2. Miami plot of statistical significance for endotoxin-related cytosine-phosphate-guanine loci stratified by hypomethylated and hypermethylated associations.
Abbreviations: CpG=cytosine-phosphate-guanine.

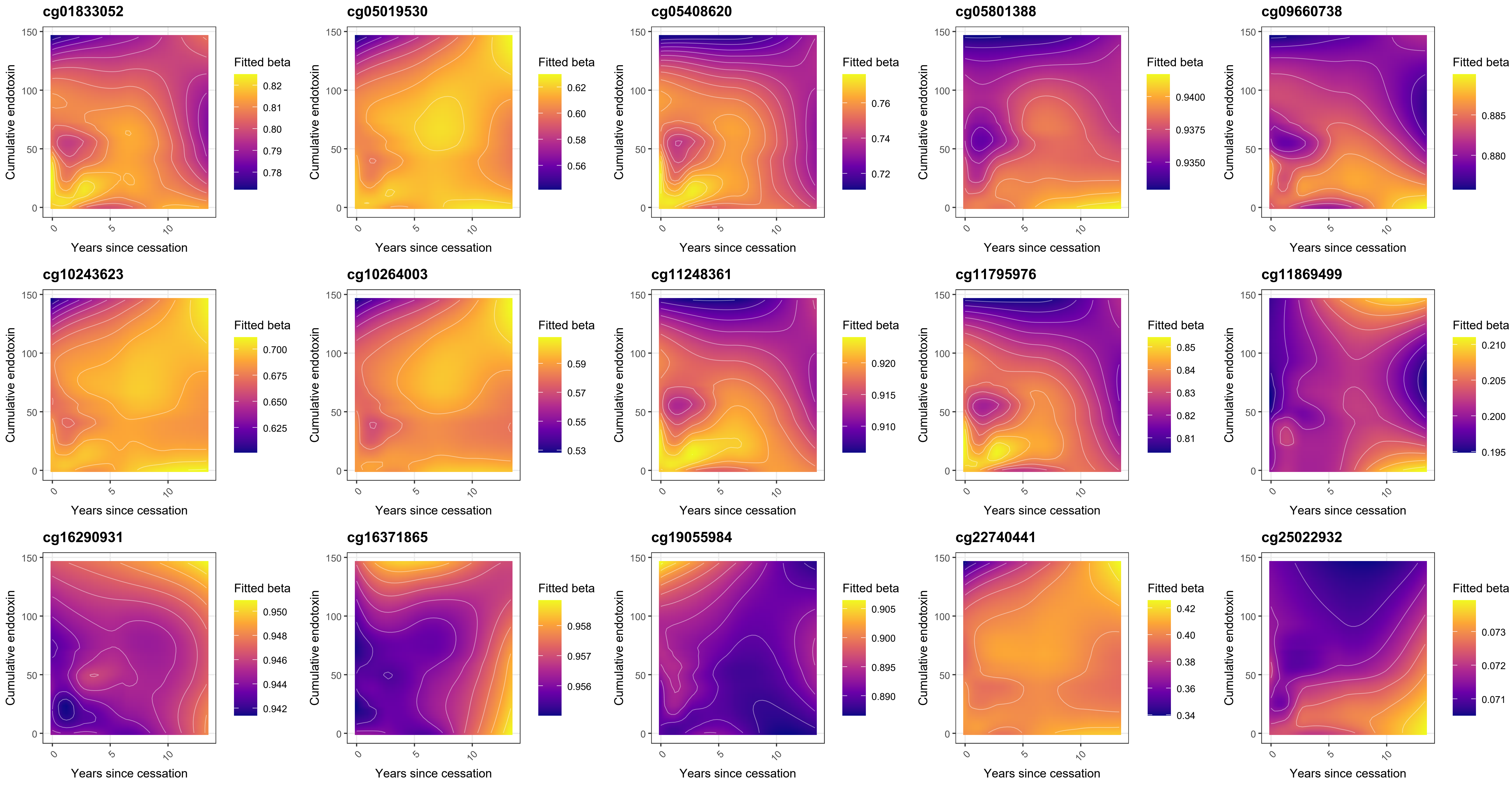

Supplemental Figure 3. Contour plots of 15 differentially methylated positions associated with endotoxin that also showed significant associations with years since cessation from endotoxin exposure.
Cumulative endotoxin is shown on the y-axis, years since cessation are shown on the x-axis, and color represents adjusted methylation beta values. Abbreviations: DMP=differentially methylated position.

medRxiv preprint doi: https://doi.org/10.64898/2026.09.29.26364351; this version posted October 1, 2026. The copyright holder for this preprint (which was not certified by peer review) is the author/funder, who has granted medRxiv a license to display the preprint in perpetuity.
This article is a US Government work. It is not subject to copyright under 17 USC 105 and is also made available for use under a CC0 license.

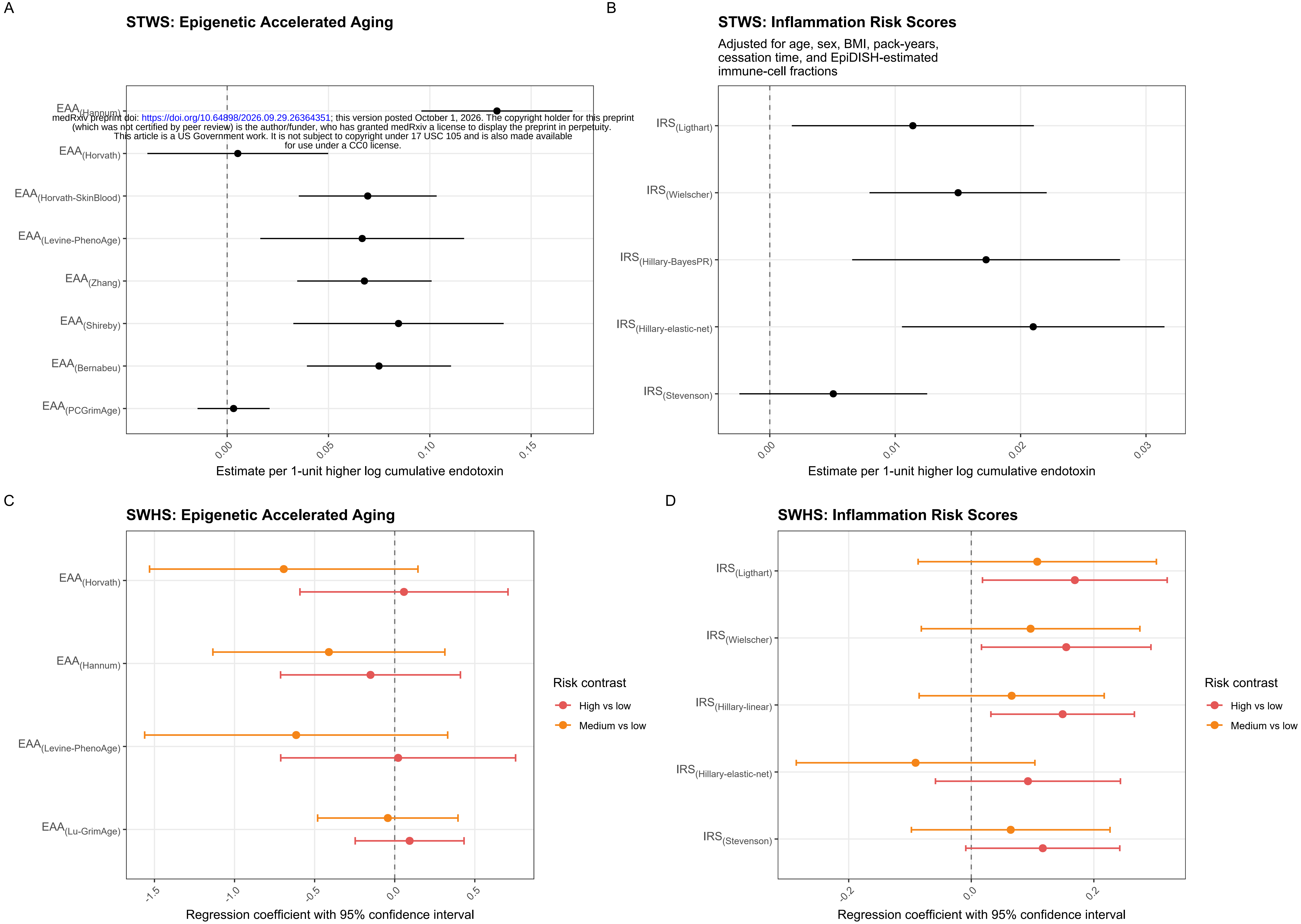

Supplemental Figure 4. Endotoxin associations with inflammation risk scores and epigenetic accelerated aging.
A) In STWS, epigenetic age acceleration was defined as the residual from regression of each epigenetic age measure on chronological age; residuals were modeled with log cumulative endotoxin, age, sex, body mass index, smoking pack-years, years since exposure cessation, and EpiDISH-estimated immune-cell fractions. B) In STWS, standardized inflammation risk scores were modeled with log cumulative endotoxin and the same covariates. C) In SWHS, epigenetic age acceleration was modeled with occupational endotoxin-risk category, age, body mass index, education, case-control status, EpiDISH-estimated immune-cell fractions, and Sample Well, with a random intercept for MatchSet. D) In SWHS, inflammation risk scores were modeled with occupational endotoxin-risk category using the same covariates and random intercept as in panel C. $\text{IRS}_{\text{(Stevenson)}}$ is an interleukin-6-based score; the other inflammation risk scores are C-reactive protein-based scores. Abbreviations: BayesPR=Bayesian penalized regression; EAA=epigenetic age acceleration; EpiDISH=Epigenetic Dissection of Intra-Sample Heterogeneity; IRS=inflammation risk score; STWS=Shanghai Textile Worker Study; SWHS=Shanghai Women's Health Study.

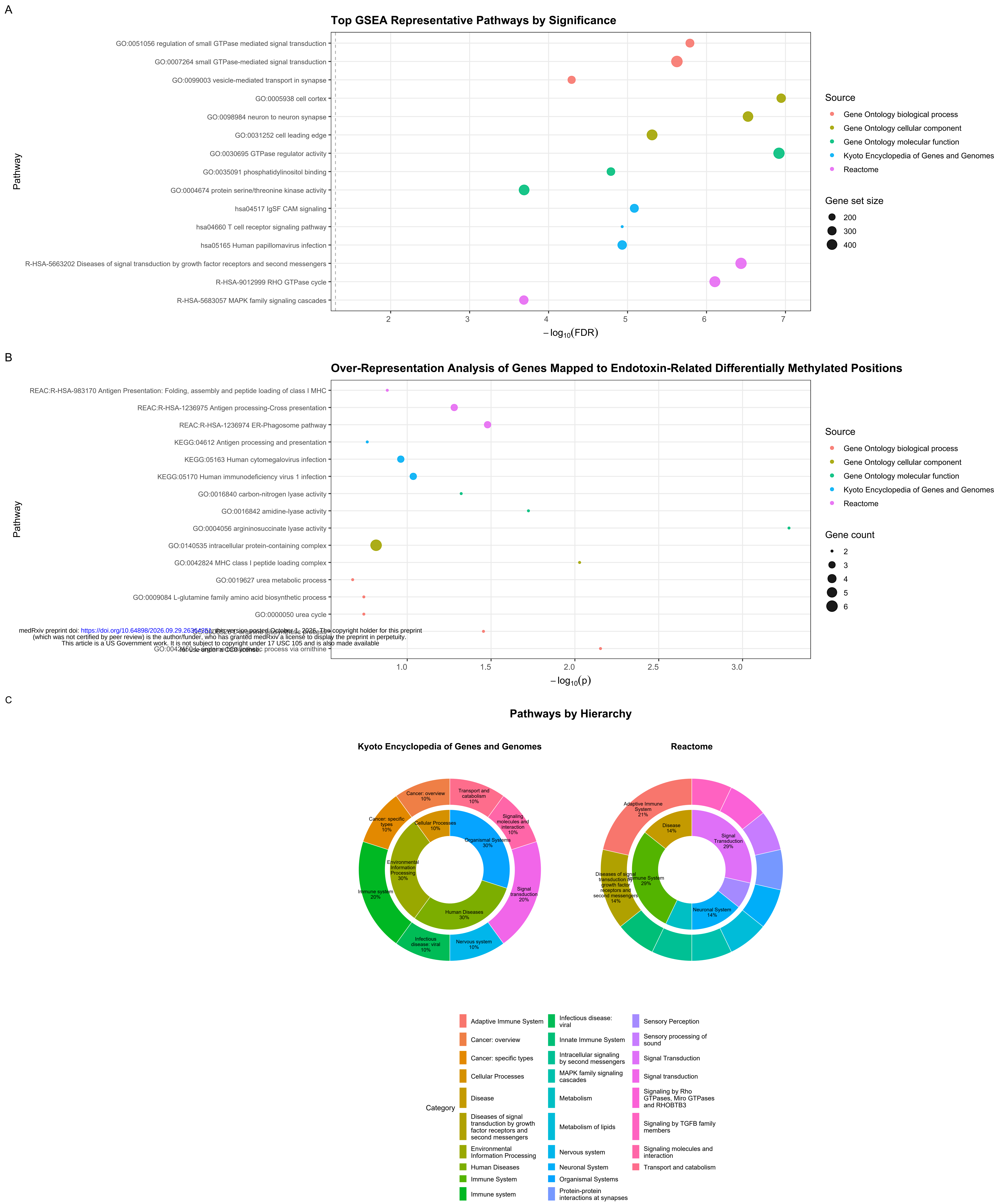

medRxiv preprint doi: https://doi.org/10.64898/2026.09.29.26364351; this version posted October 1, 2026. The copyright holder for this preprint (which was not certified by peer review) is the author/funder, who has granted medRxiv a license to display the preprint in perpetuity.
This article is a US Government work. It is not subject to copyright under 17 USC 105 and is also made available for use under a CC0 license.

Supplemental Figure 5. Pathway enrichment results for endotoxin-related DNA methylation.
A) The three most significant collapsed representative pathways from each Gene Ontology domain, Kyoto Encyclopedia of Genes and Genomes, and Reactome are shown; the x-axis gives -log10(false discovery rate), point size indicates gene-set size, and color indicates source. For GSEA, CpGs were inverse-variance pooled by gene and ranked by unsigned -log10(P). B) Over-Representation Analysis of genes mapped to the 36 differentially methylated positions. C) Broad distribution of Kyoto Encyclopedia of Genes and Genomes and Reactome pathways across two hierarchy levels after pathway collapsing. Abbreviations: CpG=cytosine-phosphate-guanine; DMP=differentially methylated position; FDR=false discovery rate; GO=Gene Ontology; GSEA=Gene Set Enrichment Analysis; KEGG=Kyoto Encyclopedia of Genes and Genomes; ORA=Over-Representation Analysis.

Occupation code	Occupation	Ordinal exposure-intensity rank	Broad group	Broad-group ordinal exposure-intensity rank
S_BL_OCC_3D_EN079	Ever/Never Veterinarians	High	Professional / technical / medical / education / arts	Low
S_BL_OCC_3D_EN433	Ever/Never Recovery of Waste Materials	High	Waste material recovery	High
S_BL_OCC_3D_EN522	Ever/Never Cleaning/Waste Disposal	High	Cleaning / waste disposal	High
S_BL_OCC_3D_EN600	Ever/Never Agric/Forestry/Animal Husbandry	High	Agriculture / forestry / animal husbandry (broad header)	High
S_BL_OCC_3D_EN611	Ever/Never Food Crops Farm Workers	High	Crop agriculture	High
S_BL_OCC_3D_EN612	Ever/Never Cotton Plantation Workers	High	Crop agriculture	High
S_BL_OCC_3D_EN613	Ever/Never Vegetables Farm Workers	High	Crop agriculture	High
S_BL_OCC_3D_EN614	Ever/Never Tea Plantation/Orchard Farmers	High	Crop agriculture	High
S_BL_OCC_3D_EN619	Ever/Never Other Farm Workers	High	Crop agriculture	High
S_BL_OCC_3D_EN631	Ever/Never Large Livestock Raisers	High	Livestock / animal husbandry	High
S_BL_OCC_3D_EN632	Ever/Never Domesticated Birds/Animals Raisers	High	Livestock / animal husbandry	High
S_BL_OCC_3D_EN633	Ever/Never Raisers Special-purposes Animals	High	Livestock / animal husbandry	High
S_BL_OCC_3D_EN634	Ever/Never Dvlpmnt Animal Specs/Body Process	High	Livestock / animal husbandry	High
S_BL_OCC_3D_EN639	Ever/Never Other Animal Husbandry Workers	High	Livestock / animal husbandry	High
S_BL_OCC_3D_EN661	Ever/Never Operators of Tractors	High	Farm machinery / field operations	Medium
S_BL_OCC_3D_EN669	Ever/Never Other Operators of Farm Machinery	High	Farm machinery / field operations	Medium
S_BL_OCC_3D_EN699	Ever/Never Other Agric/Forstry/Animl Husbandry	High	Other agriculture / forestry / animal husbandry	Medium
S_BL_OCC_3D_EN751	Ever/Never Fibers Pre-Treatment Workers	High	Textile / fiber processing	High
S_BL_OCC_3D_EN752	Ever/Never Spinning/Filature/Plying/Twisting	High	Textile / fiber processing	High
S_BL_OCC_3D_EN753	Ever/Never Instal/Maint Mach Weavng/Knitng	High	Textile / fiber processing	High
S_BL_OCC_3D_EN754	Ever/Never Cloth-Weaving Workers	High	Textile / fiber processing	High
S_BL_OCC_3D_EN755	Ever/Never Knitting Workers	High	Textile / fiber processing	High
S_BL_OCC_3D_EN756	Ever/Never Textile Printing & Dyeing Workers	High	Textile / fiber processing	High
S_BL_OCC_3D_EN759	Ever/Never Other Spinng/Weavng/Knitng/Printng	High	Textile / fiber processing	High
S_BL_OCC_3D_EN761	Ever/Never Production of leather/fur	High	Leather / fur / feather processing	Medium
S_BL_OCC_3D_EN764	Ever/Never Pre-Processing Feather & Stuffing	High	Leather / fur / feather processing	Medium
S_BL_OCC_3D_EN781	Ever/Never Rice/Flour Millers & Oil Pressers	High	Raw food / grain / animal-food processing	High

medRxiv preprint doi: https://doi.org/10.64898/2026.09.29.26364351; this version posted October 1, 2026. The copyright holder for this preprint (which was not certified by peer review) is the author/funder, who has granted medRxiv a license to display the preprint in perpetuity. This article is a US Government work. It is not subject to copyright under 17 USC 105 and is also made available for use under a CC0 license.

medRxiv preprint doi: https://doi.org/10.64898/2026.09.29.26364351; this version posted October 1, 2026. The copyright holder for this preprint (which was not certified by peer review) is the author/funder, who has granted medRxiv a license to display the preprint in perpetuity.
This article is a US Government work. It is not subject to copyright under 17 USC 105 and is also made available for use under a CC0 license.

Occupation code	Occupation	Ordinal exposure-intensity rank	Broad group	Broad-group ordinal exposure-intensity rank
S_BL_OCC_3D_EN783	Ever/Never Butchers, Animal-Food Processers	High	Raw food / grain / animal-food processing	High
S_BL_OCC_3D_EN787	Ever/Never Tea Processors	High	Other food / beverage / tobacco processing	Medium
S_BL_OCC_3D_EN790	Ever/Never Tobacco Production Workers	High	Other food / beverage / tobacco processing	Medium
S_BL_OCC_3D_EN801	Ever/Never Timber Pretreatment Workers	High	Wood / paper processing	Medium
S_BL_OCC_3D_EN805	Ever/Never Fin Wood,Bamboo,Jute,Rattan,Palm	High	Wood / paper processing	Medium
S_BL_OCC_3D_EN011	Ever/Never Researchers in Philosophy	Low	Professional / technical / medical / education / arts	Low
S_BL_OCC_3D_EN012	Ever/Never Researchers in Sociology	Low	Professional / technical / medical / education / arts	Low
S_BL_OCC_3D_EN013	Ever/Never Researchers in Economics	Low	Professional / technical / medical / education / arts	Low
S_BL_OCC_3D_EN014	Ever/Never Researchers in Law	Low	Professional / technical / medical / education / arts	Low
S_BL_OCC_3D_EN015	Ever/Never Researchers in Education	Low	Professional / technical / medical / education / arts	Low
S_BL_OCC_3D_EN016	Ever/Never Researchers in Literature & Arts	Low	Professional / technical / medical / education / arts	Low
S_BL_OCC_3D_EN017	Ever/Never Researchers Library Sci/Info Tech	Low	Professional / technical / medical / education / arts	Low
S_BL_OCC_3D_EN018	Ever/Never Researchers in History	Low	Professional / technical / medical / education / arts	Low
S_BL_OCC_3D_EN020	Ever/Never Researchers in Engineering	Low	Professional / technical / medical / education / arts	Low
S_BL_OCC_3D_EN029	Ever/Never Researchers in Other Sciences	Low	Professional / technical / medical / education / arts	Low
S_BL_OCC_3D_EN031	Ever/Never Urban Develop & Planning Persnl	Low	Professional / technical / medical / education / arts	Low
S_BL_OCC_3D_EN032	Ever/Never Civil Engineering Tech Personnel	Low	Professional / technical / medical / education / arts	Low
S_BL_OCC_3D_EN033	Ever/Never Electrical/Electronic Engineering	Low	Professional / technical / medical / education / arts	Low
S_BL_OCC_3D_EN034	Ever/Never Mechanical Engineering Personnel	Low	Professional / technical / medical / education / arts	Low
S_BL_OCC_3D_EN035	Ever/Never Chemical Engineering Personnel	Low	Professional / technical / medical / education / arts	Low
S_BL_OCC_3D_EN036	Ever/Never Metallurgical Engin Personnel	Low	Professional / technical / medical / education / arts	Low
S_BL_OCC_3D_EN037	Ever/Never Mining Engineering Personnel	Low	Professional / technical / medical / education / arts	Low
S_BL_OCC_3D_EN038	Ever/Never Indust Management Tech Personnel	Low	Professional / technical / medical / education / arts	Low
S_BL_OCC_3D_EN039	Ever/Never Mapping/Hydrology Tech Personnel	Low	Professional / technical / medical / education / arts	Low
S_BL_OCC_3D_EN040	Ever/Never Meteorology/Earthquake Tech Persnl	Low	Professional / technical / medical / education / arts	Low
S_BL_OCC_3D_EN041	Ever/Never Geological Engineering Tech Persnl	Low	Professional / technical / medical / education / arts	Low
S_BL_OCC_3D_EN045	Ever/Never Internet/Computer Hardware/Software	Low	Professional / technical / medical / education / arts	Low

medRxiv preprint doi: https://doi.org/10.64898/2026.09.29.26364351; this version posted October 1, 2026. The copyright holder for this preprint (which was not certified by peer review) is the author/funder, who has granted medRxiv a license to display the preprint in perpetuity.
This article is a US Government work. It is not subject to copyright under 17 USC 105 and is also made available for use under a CC0 license.

Occupation code	Occupation	Ordinal exposure-intensity rank	Broad group	Broad-group ordinal exposure-intensity rank
S_BL_OCC_3D_EN051	Ever/Never Science/Tech Administration Persnl	Low	Professional / technical / medical / education / arts	Low
S_BL_OCC_3D_EN052	Ever/Never Science/Tech Supporting Persnl	Low	Professional / technical / medical / education / arts	Low
S_BL_OCC_3D_EN061	Ever/Never Aircraft Pilots and Navigators	Low	Professional / technical / medical / education / arts	Low
S_BL_OCC_3D_EN062	Ever/Never Aircraft Mechanics	Low	Professional / technical / medical / education / arts	Low
S_BL_OCC_3D_EN063	Ever/Never Ship Commanders and Navigators	Low	Professional / technical / medical / education / arts	Low
S_BL_OCC_3D_EN064	Ever/Never Ship's Turbine Technology Persnl	Low	Professional / technical / medical / education / arts	Low
S_BL_OCC_3D_EN069	Ever/Never Tech Persnl in Aircraft & Ships	Low	Professional / technical / medical / education / arts	Low
S_BL_OCC_3D_EN071	Ever/Never Practitioners Chinese Trad Med	Low	Professional / technical / medical / education / arts	Low
S_BL_OCC_3D_EN072	Ever/Never Practitioners of Western Med	Low	Professional / technical / medical / education / arts	Low
S_BL_OCC_3D_EN073	Ever/Never Practit Chinese Trad Med & West Med	Low	Professional / technical / medical / education / arts	Low
S_BL_OCC_3D_EN074	Ever/Never Pharmacists	Low	Professional / technical / medical / education / arts	Low
S_BL_OCC_3D_EN075	Ever/Never Nurses	Low	Professional / technical / medical / education / arts	Low
S_BL_OCC_3D_EN076	Ever/Never Technical Experts	Low	Professional / technical / medical / education / arts	Low
S_BL_OCC_3D_EN078	Ever/Never Urban Health Technology Persnl	Low	Professional / technical / medical / education / arts	Low
S_BL_OCC_3D_EN089	Ever/Never Other Med/Health Tech Persnl	Low	Professional / technical / medical / education / arts	Low
S_BL_OCC_3D_EN091	Ever/Never Economy Planners	Low	Professional / technical / medical / education / arts	Low
S_BL_OCC_3D_EN092	Ever/Never Statisticians	Low	Professional / technical / medical / education / arts	Low
S_BL_OCC_3D_EN093	Ever/Never Accounting Personnel	Low	Professional / technical / medical / education / arts	Low
S_BL_OCC_3D_EN094	Ever/Never Personnel Workers	Low	Professional / technical / medical / education / arts	Low
S_BL_OCC_3D_EN095	Ever/Never Dispatchers	Low	Professional / technical / medical / education / arts	Low
S_BL_OCC_3D_EN096	Ever/Never Tax Collectrs/Admin Indust/Commerce	Low	Professional / technical / medical / education / arts	Low
S_BL_OCC_3D_EN097	Ever/Never Finance Workers	Low	Professional / technical / medical / education / arts	Low
S_BL_OCC_3D_EN098	Ever/Never Customs Inspectors	Low	Professional / technical / medical / education / arts	Low
S_BL_OCC_3D_EN099	Ever/Never Other Persnl Economics/Finance	Low	Professional / technical / medical / education / arts	Low
S_BL_OCC_3D_EN101	Ever/Never Judges	Low	Professional / technical / medical / education / arts	Low
S_BL_OCC_3D_EN102	Ever/Never Prosecutors	Low	Professional / technical / medical / education / arts	Low
S_BL_OCC_3D_EN103	Ever/Never Lawyers	Low	Professional / technical / medical / education / arts	Low

medRxiv preprint doi: https://doi.org/10.64898/2026.09.29.26364351; this version posted October 1, 2026. The copyright holder for this preprint (which was not certified by peer review) is the author/funder, who has granted medRxiv a license to display the preprint in perpetuity.
This article is a US Government work. It is not subject to copyright under 17 USC 105 and is also made available for use under a CC0 license.

Occupation code	Occupation	Ordinal exposure-intensity rank	Broad group	Broad-group ordinal exposure-intensity rank
S_BL_OCC_3D_EN109	Ever/Never Other Personnel Engaged in Law	Low	Professional / technical / medical / education / arts	Low
S_BL_OCC_3D_EN111	Ever/Never University-level Teachers	Low	Professional / technical / medical / education / arts	Low
S_BL_OCC_3D_EN112	Ever/Never Middle-level Teachers	Low	Professional / technical / medical / education / arts	Low
S_BL_OCC_3D_EN113	Ever/Never Elementary-level Teachers	Low	Professional / technical / medical / education / arts	Low
S_BL_OCC_3D_EN114	Ever/Never Nursery School Teachers	Low	Professional / technical / medical / education / arts	Low
S_BL_OCC_3D_EN115	Ever/Never Teachers of Blind, Deaf &/or Mute	Low	Professional / technical / medical / education / arts	Low
S_BL_OCC_3D_EN116	Ever/Never Teachers' Aids	Low	Professional / technical / medical / education / arts	Low
S_BL_OCC_3D_EN119	Ever/Never Other Teachers	Low	Professional / technical / medical / education / arts	Low
S_BL_OCC_3D_EN121	Ever/Never Lit/Arts Creation/Critics/Reviews	Low	Professional / technical / medical / education / arts	Low
S_BL_OCC_3D_EN122	Ever/Never Editors/Directrs in Perform Arts	Low	Professional / technical / medical / education / arts	Low
S_BL_OCC_3D_EN123	Ever/Never Performers	Low	Professional / technical / medical / education / arts	Low
S_BL_OCC_3D_EN124	Ever/Never Musical Instruments Performers	Low	Professional / technical / medical / education / arts	Low
S_BL_OCC_3D_EN125	Ever/Never Photographers	Low	Professional / technical / medical / education / arts	Low
S_BL_OCC_3D_EN126	Ever/Never Movies/TV Cameramen	Low	Professional / technical / medical / education / arts	Low
S_BL_OCC_3D_EN127	Ever/Never Fine Artists	Low	Professional / technical / medical / education / arts	Low
S_BL_OCC_3D_EN128	Ever/Never Stage Workers	Low	Professional / technical / medical / education / arts	Low
S_BL_OCC_3D_EN129	Ever/Never Sports Coaches and Umpires	Low	Professional / technical / medical / education / arts	Low
S_BL_OCC_3D_EN130	Ever/Never Athletes	Low	Professional / technical / medical / education / arts	Low
S_BL_OCC_3D_EN139	Ever/Never Other Literary/Arts/Sports Persnl	Low	Professional / technical / medical / education / arts	Low
S_BL_OCC_3D_EN141	Ever/Never Reporters	Low	Professional / technical / medical / education / arts	Low
S_BL_OCC_3D_EN142	Ever/Never Editors	Low	Professional / technical / medical / education / arts	Low
S_BL_OCC_3D_EN143	Ever/Never Broadcasters	Low	Professional / technical / medical / education / arts	Low
S_BL_OCC_3D_EN144	Ever/Never Translators & Interpreters	Low	Professional / technical / medical / education / arts	Low
S_BL_OCC_3D_EN145	Ever/Never Librarians & Library Workers	Low	Professional / technical / medical / education / arts	Low
S_BL_OCC_3D_EN146	Ever/Never Files/Archives Management Workrs	Low	Professional / technical / medical / education / arts	Low
S_BL_OCC_3D_EN147	Ever/Never Archeol/Cultural Relics Workrs	Low	Professional / technical / medical / education / arts	Low
S_BL_OCC_3D_EN148	Ever/Never Exhibition Tour Guides	Low	Professional / technical / medical / education / arts	Low

medRxiv preprint doi: https://doi.org/10.64898/2026.09.29.26364351; this version posted October 1, 2026. The copyright holder for this preprint (which was not certified by peer review) is the author/funder, who has granted medRxiv a license to display the preprint in perpetuity.
This article is a US Government work. It is not subject to copyright under 17 USC 105 and is also made available for use under a CC0 license.

Occupation code	Occupation	Ordinal exposure-intensity rank	Broad group	Broad-group ordinal exposure-intensity rank
S_BL_OCC_3D_EN149	Ever/Never Other Cultural Workers	Low	Professional / technical / medical / education / arts	Low
S_BL_OCC_3D_EN150	Ever/Never Professional Religious Workers	Low	Professional / technical / medical / education / arts	Low
S_BL_OCC_3D_EN211	Ever/Never In Charge Central Gov Bodies	Low	Administrative / managerial	Low
S_BL_OCC_3D_EN212	Ever/Never In Charge Prov-Level State Bodies	Low	Administrative / managerial	Low
S_BL_OCC_3D_EN213	Ever/Never In Charge Reg-/City-/Coun-Level St	Low	Administrative / managerial	Low
S_BL_OCC_3D_EN221	Ever/Never In Charge Chinese Communist Party	Low	Administrative / managerial	Low
S_BL_OCC_3D_EN222	Ever/Never Charge Chinese Communist Youth Lgue	Low	Administrative / managerial	Low
S_BL_OCC_3D_EN223	Ever/Never In Charge Chinese Democracy Party	Low	Administrative / managerial	Low
S_BL_OCC_3D_EN231	Ever/Never In Charge Urban Neighborhoods	Low	Administrative / managerial	Low
S_BL_OCC_3D_EN232	Ever/Never In Charge of Res Committees	Low	Administrative / managerial	Low
S_BL_OCC_3D_EN233	Ever/Never In Charge of Rural Communes	Low	Administrative / managerial	Low
S_BL_OCC_3D_EN241	Ever/Never In Charge Enterprise/Instit Units	Low	Administrative / managerial	Low
S_BL_OCC_3D_EN242	Ever/Never In Charge Wrk Orgs/Enterprise Units	Low	Administrative / managerial	Low
S_BL_OCC_3D_EN311	Ever/Never Office Workers in Administration	Low	Clerical / security / postal / telecom	Low
S_BL_OCC_3D_EN312	Ever/Never Secretaries	Low	Clerical / security / postal / telecom	Low
S_BL_OCC_3D_EN313	Ever/Never Mail Clerks & Communic Workers	Low	Clerical / security / postal / telecom	Low
S_BL_OCC_3D_EN314	Ever/Never Typists, Mimeographers	Low	Clerical / security / postal / telecom	Low
S_BL_OCC_3D_EN319	Ever/Never Other Admin Office Workers	Low	Clerical / security / postal / telecom	Low
S_BL_OCC_3D_EN320	Ever/Never Armymen	Low	Clerical / security / postal / telecom	Low
S_BL_OCC_3D_EN321	Ever/Never Political Workers	Low	Clerical / security / postal / telecom	Low
S_BL_OCC_3D_EN322	Ever/Never Persnl [cadre] Management Workrs	Low	Clerical / security / postal / telecom	Low
S_BL_OCC_3D_EN323	Ever/Never Policemen/Pub Security Workers	Low	Clerical / security / postal / telecom	Low
S_BL_OCC_3D_EN324	Ever/Never Firemen	Low	Clerical / security / postal / telecom	Low
S_BL_OCC_3D_EN329	Ever/Never Other Polit & Security Workers	Low	Clerical / security / postal / telecom	Low
S_BL_OCC_3D_EN331	Ever/Never Postal Service Workers	Low	Clerical / security / postal / telecom	Low
S_BL_OCC_3D_EN332	Ever/Never Telecommunication Service Workrs	Low	Clerical / security / postal / telecom	Low
S_BL_OCC_3D_EN339	Ever/Never Other Postal/Telecomm Workers	Low	Clerical / security / postal / telecom	Low

medRxiv preprint doi: https://doi.org/10.64898/2026.09.29.26364351; this version posted October 1, 2026. The copyright holder for this preprint (which was not certified by peer review) is the author/funder, who has granted medRxiv a license to display the preprint in perpetuity.
This article is a US Government work. It is not subject to copyright under 17 USC 105 and is also made available for use under a CC0 license.

Occupation code	Occupation	Ordinal exposure-intensity rank	Broad group	Broad-group ordinal exposure-intensity rank
S_BL_OCC_3D_EN391	Ever/Never Non-Technical Managing Personnel	Low	Clerical / security / postal / telecom	Low
S_BL_OCC_3D_EN399	Ever/Never Other Affairs Handler Workers	Low	Clerical / security / postal / telecom	Low
S_BL_OCC_3D_EN411	Ever/Never Business Operators, Sales Persons	Low	Commerce / sales / purchasing	Low
S_BL_OCC_3D_EN419	Ever/Never Other Persons Engaged in Sales	Low	Commerce / sales / purchasing	Low
S_BL_OCC_3D_EN421	Ever/Never Purchasing Agents	Low	Commerce / sales / purchasing	Low
S_BL_OCC_3D_EN422	Ever/Never Persons in Supplying/Marketing	Low	Commerce / sales / purchasing	Low
S_BL_OCC_3D_EN432	Ever/Never Buyers on CONSIGNMENTS	Low	Commerce / sales / purchasing	Low
S_BL_OCC_3D_EN499	Ever/Never Other Persons in Business	Low	Commerce / sales / purchasing	Low
S_BL_OCC_3D_EN511	Ever/Never Service Persnl in Restaurants	Low	General service work	Low
S_BL_OCC_3D_EN512	Ever/Never Service Hotels/Guest Houses/etc	Low	General service work	Low
S_BL_OCC_3D_EN513	Ever/Never Service Theaters/Pub Displays	Low	General service work	Low
S_BL_OCC_3D_EN514	Ever/Never Service Persnl in Bath Houses	Low	General service work	Low
S_BL_OCC_3D_EN515	Ever/Never Service Vehicles/Ships/Airplanes	Low	General service work	Low
S_BL_OCC_3D_EN516	Ever/Never Ticket Sellers	Low	General service work	Low
S_BL_OCC_3D_EN517	Ever/Never Nursery Child Care Workers	Low	General service work	Low
S_BL_OCC_3D_EN518	Ever/Never Nannies	Low	General service work	Low
S_BL_OCC_3D_EN519	Ever/Never Barbers & Hairdressers	Low	General service work	Low
S_BL_OCC_3D_EN520	Ever/Never Laundering/Dyeing/Repair Garments	Low	General service work	Low
S_BL_OCC_3D_EN521	Ever/Never Suppliers of Fuels	Low	General service work	Low
S_BL_OCC_3D_EN523	Ever/Never Odds-and-Ends Workers	Low	General service work	Low
S_BL_OCC_3D_EN529	Ever/Never Other Service Personnel	Low	General service work	Low
S_BL_OCC_3D_EN530	Ever/Never Chefs and Cooks	Low	General service work	Low
S_BL_OCC_3D_EN540	Ever/Never Tour Guides	Low	General service work	Low
S_BL_OCC_3D_EN551	Ever/Never Repairers Mech/Elec Consumer Prods	Low	General service work	Low
S_BL_OCC_3D_EN559	Ever/Never Other Repairers Everyday Articles	Low	General service work	Low
S_BL_OCC_3D_EN599	Ever/Never Other Service Personnel	Low	General service work	Low
S_BL_OCC_3D_EN623	Ever/Never Forest Protection Personnel	Low	Forestry	Medium

medRxiv preprint doi: https://doi.org/10.64898/2026.09.29.26364351; this version posted October 1, 2026. The copyright holder for this preprint (which was not certified by peer review) is the author/funder, who has granted medRxiv a license to display the preprint in perpetuity. This article is a US Government work. It is not subject to copyright under 17 USC 105 and is also made available for use under a CC0 license.

Occupation code	Occupation	Ordinal exposure-intensity rank	Broad group	Broad-group ordinal exposure-intensity rank
S_BL_OCC_3D_EN650	Ever/Never Hunters	Low	Hunting	Low
S_BL_OCC_3D_EN711	Ever/Never Mining, Quarrying	Low	Mining / metals / chemicals / rubber / plastics	Low
S_BL_OCC_3D_EN712	Ever/Never Ores and Rocks Processing	Low	Mining / metals / chemicals / rubber / plastics	Low
S_BL_OCC_3D_EN713	Ever/Never Extraction of Oil, Natural Gas	Low	Mining / metals / chemicals / rubber / plastics	Low
S_BL_OCC_3D_EN714	Ever/Never Geological Exploration	Low	Mining / metals / chemicals / rubber / plastics	Low
S_BL_OCC_3D_EN715	Ever/Never Salt-Mining	Low	Mining / metals / chemicals / rubber / plastics	Low
S_BL_OCC_3D_EN719	Ever/Never Other Mining/Quarrying/Exploration	Low	Mining / metals / chemicals / rubber / plastics	Low
S_BL_OCC_3D_EN721	Ever/Never Smelters	Low	Mining / metals / chemicals / rubber / plastics	Low
S_BL_OCC_3D_EN722	Ever/Never Rolling Mill/Machinery Operators	Low	Mining / metals / chemicals / rubber / plastics	Low
S_BL_OCC_3D_EN723	Ever/Never Foundry Workers	Low	Mining / metals / chemicals / rubber / plastics	Low
S_BL_OCC_3D_EN724	Ever/Never Heat Treatment of Metals	Low	Mining / metals / chemicals / rubber / plastics	Low
S_BL_OCC_3D_EN725	Ever/Never Drawing & Extruding of Metals	Low	Mining / metals / chemicals / rubber / plastics	Low
S_BL_OCC_3D_EN726	Ever/Never Surface Treatment of Metals	Low	Mining / metals / chemicals / rubber / plastics	Low
S_BL_OCC_3D_EN729	Ever/Never Other Ore Smelting/Metal Process	Low	Mining / metals / chemicals / rubber / plastics	Low
S_BL_OCC_3D_EN731	Ever/Never Basic Chem Raw Materials Productn	Low	Mining / metals / chemicals / rubber / plastics	Low
S_BL_OCC_3D_EN732	Ever/Never Chemical Fertilizers Production	Low	Mining / metals / chemicals / rubber / plastics	Low
S_BL_OCC_3D_EN733	Ever/Never Farm Chemicals Production Workers	Low	Mining / metals / chemicals / rubber / plastics	Low
S_BL_OCC_3D_EN734	Ever/Never Everyday Use Chemicals Production	Low	Mining / metals / chemicals / rubber / plastics	Low
S_BL_OCC_3D_EN735	Ever/Never Chemical Fibers Production Workers	Low	Mining / metals / chemicals / rubber / plastics	Low
S_BL_OCC_3D_EN736	Ever/Never Oil Refinery Workers	Low	Mining / metals / chemicals / rubber / plastics	Low
S_BL_OCC_3D_EN737	Ever/Never Coking Workers	Low	Mining / metals / chemicals / rubber / plastics	Low
S_BL_OCC_3D_EN738	Ever/Never Pharmaceutical Workers	Low	Mining / metals / chemicals / rubber / plastics	Low
S_BL_OCC_3D_EN739	Ever/Never Other Chemicals Workers	Low	Mining / metals / chemicals / rubber / plastics	Low
S_BL_OCC_3D_EN741	Ever/Never Rubber Process Machine Operators	Low	Mining / metals / chemicals / rubber / plastics	Low
S_BL_OCC_3D_EN742	Ever/Never Plastic Process Machine Operators	Low	Mining / metals / chemicals / rubber / plastics	Low
S_BL_OCC_3D_EN743	Ever/Never Tire Production/Vulcanization	Low	Mining / metals / chemicals / rubber / plastics	Low
S_BL_OCC_3D_EN749	Ever/Never Other Rubber/Plastic Production	Low	Mining / metals / chemicals / rubber / plastics	Low

medRxiv preprint doi: https://doi.org/10.64898/2026.09.29.26364351; this version posted October 1, 2026. The copyright holder for this preprint (which was not certified by peer review) is the author/funder, who has granted medRxiv a license to display the preprint in perpetuity. This article is a US Government work. It is not subject to copyright under 17 USC 105 and is also made available for use under a CC0 license.

Occupation code	Occupation	Ordinal exposure-intensity rank	Broad group	Broad-group ordinal exposure-intensity rank
S_BL_OCC_3D_EN803	Ever/Never Wood Furniture Workers	Low	Wood / paper processing	Medium
S_BL_OCC_3D_EN804	Ever/Never Construction Carpenters	Low	Wood / paper processing	Medium
S_BL_OCC_3D_EN821	Ever/Never Character Selection & Typesetting	Low	Printing / plate making	Low
S_BL_OCC_3D_EN822	Ever/Never Plate Makers	Low	Printing / plate making	Low
S_BL_OCC_3D_EN823	Ever/Never Printing Workers	Low	Printing / plate making	Low
S_BL_OCC_3D_EN824	Ever/Never Binders	Low	Printing / plate making	Low
S_BL_OCC_3D_EN825	Ever/Never Dark Room Photography Workers	Low	Printing / plate making	Low
S_BL_OCC_3D_EN829	Ever/Never Other Printers & Related Workers	Low	Printing / plate making	Low
S_BL_OCC_3D_EN831	Ever/Never Stonecutters	Low	Stone / glass / ceramics	Low
S_BL_OCC_3D_EN832	Ever/Never Stone Craftsmen	Low	Stone / glass / ceramics	Low
S_BL_OCC_3D_EN839	Ever/Never Other Stone Workers	Low	Stone / glass / ceramics	Low
S_BL_OCC_3D_EN841	Ever/Never Forgers & Forging Press Operators	Low	Machinery / electrical / electronic installation & maintenance	Low
S_BL_OCC_3D_EN842	Ever/Never Fitters	Low	Machinery / electrical / electronic installation & maintenance	Low
S_BL_OCC_3D_EN843	Ever/Never Machine Tool Installers	Low	Machinery / electrical / electronic installation & maintenance	Low
S_BL_OCC_3D_EN844	Ever/Never Metal-cut Mach Tools/Blade Sharpen	Low	Machinery / electrical / electronic installation & maintenance	Low
S_BL_OCC_3D_EN849	Ever/Never Other Forge/Tool/Mach Tool Install	Low	Machinery / electrical / electronic installation & maintenance	Low
S_BL_OCC_3D_EN851	Ever/Never Machinery Installation & Assembly	Low	Machinery / electrical / electronic installation & maintenance	Low
S_BL_OCC_3D_EN852	Ever/Never Machinery Maintenance Workers	Low	Machinery / electrical / electronic installation & maintenance	Low
S_BL_OCC_3D_EN853	Ever/Never Clocks & Watches Instrument Prod	Low	Machinery / electrical / electronic installation & maintenance	Low
S_BL_OCC_3D_EN859	Ever/Never Other Instal/Assembly Mach Product	Low	Machinery / electrical / electronic installation & maintenance	Low
S_BL_OCC_3D_EN861	Ever/Never Install/Maint Electric Equip	Low	Machinery / electrical / electronic installation & maintenance	Low
S_BL_OCC_3D_EN862	Ever/Never Install/Maint Electronic Equip	Low	Machinery / electrical / electronic installation & maintenance	Low
S_BL_OCC_3D_EN863	Ever/Never Assembly Electric/Electron Equip	Low	Machinery / electrical / electronic installation & maintenance	Low
S_BL_OCC_3D_EN864	Ever/Never Electricians	Low	Machinery / electrical / electronic installation & maintenance	Low
S_BL_OCC_3D_EN865	Ever/Never Instal/Maint Phone/Telegraph Equip	Low	Machinery / electrical / electronic installation & maintenance	Low
S_BL_OCC_3D_EN869	Ever/Never Oth Elect Equip Instal/Maint	Low	Machinery / electrical / electronic installation & maintenance	Low
S_BL_OCC_3D_EN871	Ever/Never Transmission Equipment Operators	Low	Equipment operation / media / tape production	Low

medRxiv preprint doi: https://doi.org/10.64898/2026.09.29.26364351; this version posted October 1, 2026. The copyright holder for this preprint (which was not certified by peer review) is the author/funder, who has granted medRxiv a license to display the preprint in perpetuity.
This article is a US Government work. It is not subject to copyright under 17 USC 105 and is also made available for use under a CC0 license.

Occupation code	Occupation	Ordinal exposure-intensity rank	Broad group	Broad-group ordinal exposure-intensity rank
S_BL_OCC_3D_EN872	Ever/Never Sound Recording Equipment Operators	Low	Equipment operation / media / tape production	Low
S_BL_OCC_3D_EN873	Ever/Never Motion Picture Projector Operators	Low	Equipment operation / media / tape production	Low
S_BL_OCC_3D_EN874	Ever/Never Tape Making Workers	Low	Equipment operation / media / tape production	Low
S_BL_OCC_3D_EN881	Ever/Never Workers in Ductworks and Pipelines	Low	Metal fabrication / welding / ductwork	Low
S_BL_OCC_3D_EN882	Ever/Never Welders	Low	Metal fabrication / welding / ductwork	Low
S_BL_OCC_3D_EN883	Ever/Never Cold Working Workers	Low	Metal fabrication / welding / ductwork	Low
S_BL_OCC_3D_EN884	Ever/Never Makers/Installers Metal Components	Low	Metal fabrication / welding / ductwork	Low
S_BL_OCC_3D_EN891	Ever/Never Glass Workers	Low	Glass / pottery / enamel	Low
S_BL_OCC_3D_EN892	Ever/Never Pottery and Porcelain Workers	Low	Glass / pottery / enamel	Low
S_BL_OCC_3D_EN893	Ever/Never Enamel Workers	Low	Glass / pottery / enamel	Low
S_BL_OCC_3D_EN901	Ever/Never Painters	Low	Painting / non-metal mineral / other production	Low
S_BL_OCC_3D_EN909	Ever/Never Other Painters	Low	Painting / non-metal mineral / other production	Low
S_BL_OCC_3D_EN911	Ever/Never Production Cement & Cement Products	Low	Painting / non-metal mineral / other production	Low
S_BL_OCC_3D_EN912	Ever/Never Prod Asbestos & Asbestos Products	Low	Painting / non-metal mineral / other production	Low
S_BL_OCC_3D_EN913	Ever/Never Production Bricks/Tiles/Lime	Low	Painting / non-metal mineral / other production	Low
S_BL_OCC_3D_EN914	Ever/Never Production Refractory Materials	Low	Painting / non-metal mineral / other production	Low
S_BL_OCC_3D_EN915	Ever/Never Prod Other Non-Metal Mineral Prods	Low	Painting / non-metal mineral / other production	Low
S_BL_OCC_3D_EN916	Ever/Never Prod Cultural/Educ/Sports Articles	Low	Painting / non-metal mineral / other production	Low
S_BL_OCC_3D_EN917	Ever/Never Jewelers	Low	Painting / non-metal mineral / other production	Low
S_BL_OCC_3D_EN918	Ever/Never Engravers of Artistic Works	Low	Painting / non-metal mineral / other production	Low
S_BL_OCC_3D_EN919	Ever/Never Other Production & Related Workers	Low	Painting / non-metal mineral / other production	Low
S_BL_OCC_3D_EN921	Ever/Never Tile Workers, Plasterers	Low	Construction	Low
S_BL_OCC_3D_EN922	Ever/Never Concrete Workers	Low	Construction	Low
S_BL_OCC_3D_EN923	Ever/Never Steel Benders	Low	Construction	Low
S_BL_OCC_3D_EN924	Ever/Never Scaffolders	Low	Construction	Low
S_BL_OCC_3D_EN925	Ever/Never Laborers Engaged in Heavy Work	Low	Construction	Low
S_BL_OCC_3D_EN929	Ever/Never Other Construction Workers	Low	Construction	Low

medRxiv preprint doi: https://doi.org/10.64898/2026.09.29.26364351; this version posted October 1, 2026. The copyright holder for this preprint (which was not certified by peer review) is the author/funder, who has granted medRxiv a license to display the preprint in perpetuity.
This article is a US Government work. It is not subject to copyright under 17 USC 105 and is also made available for use under a CC0 license.

Occupation code	Occupation	Ordinal exposure-intensity rank	Broad group	Broad-group ordinal exposure-intensity rank
S_BL_OCC_3D_EN931	Ever/Never Electricity Generation Equipment	Low	Power generation / boiler operations	Low
S_BL_OCC_3D_EN932	Ever/Never Around-the-Clock Power Networks	Low	Power generation / boiler operations	Low
S_BL_OCC_3D_EN933	Ever/Never Boiler Workers	Low	Power generation / boiler operations	Low
S_BL_OCC_3D_EN939	Ever/Never Other Power Generation Equipment	Low	Power generation / boiler operations	Low
S_BL_OCC_3D_EN941	Ever/Never Loading/Unloading Workers & Movers	Low	Loading / transport / shipping	Low
S_BL_OCC_3D_EN942	Ever/Never Crane Operators	Low	Loading / transport / shipping	Low
S_BL_OCC_3D_EN943	Ever/Never Earth-moving Mach & Related Equip	Low	Loading / transport / shipping	Low
S_BL_OCC_3D_EN949	Ever/Never Other Loading/Unloading Workers	Low	Loading / transport / shipping	Low
S_BL_OCC_3D_EN951	Ever/Never Ship Crew	Low	Loading / transport / shipping	Low
S_BL_OCC_3D_EN952	Ever/Never Locomotive Drivers & Stokers	Low	Loading / transport / shipping	Low
S_BL_OCC_3D_EN953	Ever/Never Railroad Transportation Workers	Low	Loading / transport / shipping	Low
S_BL_OCC_3D_EN954	Ever/Never Motor Vehicle Drivers	Low	Loading / transport / shipping	Low
S_BL_OCC_3D_EN955	Ever/Never Power-driven Vehicle Drivers	Low	Loading / transport / shipping	Low
S_BL_OCC_3D_EN957	Ever/Never Man-powered Vehicle Workers	Low	Loading / transport / shipping	Low
S_BL_OCC_3D_EN959	Ever/Never Other Equipment Used in Transport	Low	Loading / transport / shipping	Low
S_BL_OCC_3D_EN961	Ever/Never Examiners/Inspectors	Low	Inspection / testing / laboratory analysis	Low
S_BL_OCC_3D_EN962	Ever/Never Measurers	Low	Inspection / testing / laboratory analysis	Low
S_BL_OCC_3D_EN966	Ever/Never Other Examiners	Low	Inspection / testing / laboratory analysis	Low
S_BL_OCC_3D_EN967	Ever/Never Chemical Testers	Low	Inspection / testing / laboratory analysis	Low
S_BL_OCC_3D_EN991	Ever/Never Workers in Underwater Operations	Low	Miscellaneous / warehouse / underwater	Low
S_BL_OCC_3D_EN992	Ever/Never Mapping Workers	Low	Miscellaneous / warehouse / underwater	Low
S_BL_OCC_3D_EN993	Ever/Never Workers in Packaging/Baling	Low	Miscellaneous / warehouse / underwater	Low
S_BL_OCC_3D_EN994	Ever/Never Warehouse Workers	Low		
S_BL_OCC_3D_EN997	Ever/Never Other Production/Transport Workers	Low		
S_BL_OCC_3D_EN998	Ever/Never Miscellaneous other workers	Low		
S_BL_OCC_3D_EN019	Ever/Never Researchers in Science	Medium	Professional / technical / medical / education / arts	Low
S_BL_OCC_3D_EN021	Ever/Never Researchers in Agriculture	Medium	Professional / technical / medical / education / arts	Low

medRxiv preprint doi: https://doi.org/10.64898/2026.09.29.26364351; this version posted October 1, 2026. The copyright holder for this preprint (which was not certified by peer review) is the author/funder, who has granted medRxiv a license to display the preprint in perpetuity. This article is a US Government work. It is not subject to copyright under 17 USC 105 and is also made available for use under a CC0 license.

Occupation code	Occupation	Ordinal exposure-intensity rank	Broad group	Broad-group ordinal exposure-intensity rank
S_BL_OCC_3D_EN022	Ever/Never Researchers in Medicine	Medium	Professional / technical / medical / education / arts	Low
S_BL_OCC_3D_EN042	Ever/Never Light Indust & Textile Tech Persnl	Medium	Professional / technical / medical / education / arts	Low
S_BL_OCC_3D_EN043	Ever/Never Agriculture Technology Personnel	Medium	Professional / technical / medical / education / arts	Low
S_BL_OCC_3D_EN044	Ever/Never Forestry Technology Personnel	Medium	Professional / technical / medical / education / arts	Low
S_BL_OCC_3D_EN049	Ever/Never Other Tech Persnl Eng/Agric/Forstry	Medium	Professional / technical / medical / education / arts	Low
S_BL_OCC_3D_EN077	Ever/Never Persnl Hygiene/Anti-Epidemic Work	Medium	Professional / technical / medical / education / arts	Low
S_BL_OCC_3D_EN431	Ever/Never Buyers of Farm/Sideline Products	Medium	Commerce / sales / purchasing	Low
S_BL_OCC_3D_EN524	Ever/Never Funeral and Burial Workers	Medium	General service work	Low
S_BL_OCC_3D_EN621	Ever/Never Forest Raising/Planting Persnl	Medium	Forestry	Medium
S_BL_OCC_3D_EN622	Ever/Never Tree Felling Personnel	Medium	Forestry	Medium
S_BL_OCC_3D_EN624	Ever/Never Water Transport of Timber	Medium	Forestry	Medium
S_BL_OCC_3D_EN629	Ever/Never Other Forestry Workers	Medium	Forestry	Medium
S_BL_OCC_3D_EN641	Ever/Never Aquatic Plants/Animals Raisers	Medium	Fishery / aquatic products	Medium
S_BL_OCC_3D_EN642	Ever/Never Fishing/Capturing Aquatic Animals	Medium	Fishery / aquatic products	Medium
S_BL_OCC_3D_EN643	Ever/Never Collect/Harvest Natural Aqua Prod	Medium	Fishery / aquatic products	Medium
S_BL_OCC_3D_EN644	Ever/Never Operators Motorized Fishing Boats	Medium	Fishery / aquatic products	Medium
S_BL_OCC_3D_EN649	Ever/Never Other Fish/Captre/Harvst Aqua Prods	Medium	Fishery / aquatic products	Medium
S_BL_OCC_3D_EN762	Ever/Never Production finished leather goods	Medium	Leather / fur / feather processing	Medium
S_BL_OCC_3D_EN763	Ever/Never Production finished fur goods	Medium	Leather / fur / feather processing	Medium
S_BL_OCC_3D_EN769	Ever/Never Other Leathers/Furs/Finished Prod	Medium	Leather / fur / feather processing	Medium
S_BL_OCC_3D_EN771	Ever/Never Pattern Makers, Tailoring Workers	Medium	Tailoring / sewing	Medium
S_BL_OCC_3D_EN772	Ever/Never Sewing Workers	Medium	Tailoring / sewing	Medium
S_BL_OCC_3D_EN773	Ever/Never Shoemakers, Hat-Makers	Medium	Tailoring / sewing	Medium
S_BL_OCC_3D_EN774	Ever/Never Embroidery Workers	Medium	Tailoring / sewing	Medium
S_BL_OCC_3D_EN779	Ever/Never Other Tailoring and Sewing Workers	Medium	Tailoring / sewing	Medium
S_BL_OCC_3D_EN782	Ever/Never Sugar Production Workers	Medium	Raw food / grain / animal-food processing	High
S_BL_OCC_3D_EN784	Ever/Never Pickling/Canning/Preserv Food	Medium	Raw food / grain / animal-food processing	High

medRxiv preprint doi: https://doi.org/10.64898/2026.09.29.26364351; this version posted October 1, 2026. The copyright holder for this preprint (which was not certified by peer review) is the author/funder, who has granted medRxiv a license to display the preprint in perpetuity.
This article is a US Government work. It is not subject to copyright under 17 USC 105 and is also made available for use under a CC0 license.

Occupation code	Occupation	Ordinal exposure-intensity rank	Broad group	Broad-group ordinal exposure-intensity rank
S_BL_OCC_3D_EN785	Ever/Never Processing Milk Products	Medium	Raw food / grain / animal-food processing	High
S_BL_OCC_3D_EN786	Ever/Never Bakers, Confectioners	Medium	Other food / beverage / tobacco processing	Medium
S_BL_OCC_3D_EN788	Ever/Never Wine-Makers/Brewers/Beverage Prod	Medium	Other food / beverage / tobacco processing	Medium
S_BL_OCC_3D_EN789	Ever/Never Other Food/Beverage Production	Medium	Other food / beverage / tobacco processing	Medium
S_BL_OCC_3D_EN802	Ever/Never Process Timber into Construct Prod	Medium	Wood / paper processing	Medium
S_BL_OCC_3D_EN809	Ever/Never Other Timber/Finished Prods	Medium	Wood / paper processing	Medium
S_BL_OCC_3D_EN811	Ever/Never Paper-making Workers	Medium	Wood / paper processing	Medium
S_BL_OCC_3D_EN812	Ever/Never Paper Products Workers	Medium	Wood / paper processing	Medium
S_BL_OCC_3D_EN819	Ever/Never Other Paper-making & Paper Prod	Medium	Wood / paper processing	Medium
S_BL_OCC_3D_EN956	Ever/Never Drivers of Animal-Powered Vehicles	Medium	Loading / transport / shipping	Low
S_BL_OCC_3D_EN963	Ever/Never Testers/Experimenters	Medium	Inspection / testing / laboratory analysis	Low
S_BL_OCC_3D_EN964	Ever/Never Lab Technicians/Analyzers	Medium	Inspection / testing / laboratory analysis	Low
S_BL_OCC_3D_EN965	Ever/Never Chemical Examiners	Medium	Inspection / testing / laboratory analysis	Low
